# Fluorogenic *In Situ* Thioacetalization: Expanding the Chemical Space of Fluorescent Probes, Including Unorthodox, Bifurcated, and Mechanosensitive Chalcogen Bonds

**DOI:** 10.1021/jacsau.3c00364

**Published:** 2023-08-21

**Authors:** Xiao-Xiao Chen, Rosa M. Gomila, Juan Manuel García-Arcos, Maxime Vonesch, Nerea Gonzalez-Sanchis, Aurelien Roux, Antonio Frontera, Naomi Sakai, Stefan Matile

**Affiliations:** † Department of Organic Chemistry, University of Geneva, 1211 Geneva, Switzerland; ‡ Departament de Química, Universitat de les Illes Balears, SP-07122 Palma de Mallorca, Spain; § Department of Biochemistry, University of Geneva, 1211 Geneva, Switzerland

**Keywords:** fluorescent probes, chalcogen bonds, thioacetals, bioimaging, membrane tension, mechanosensitivity, turn-on donors

## Abstract

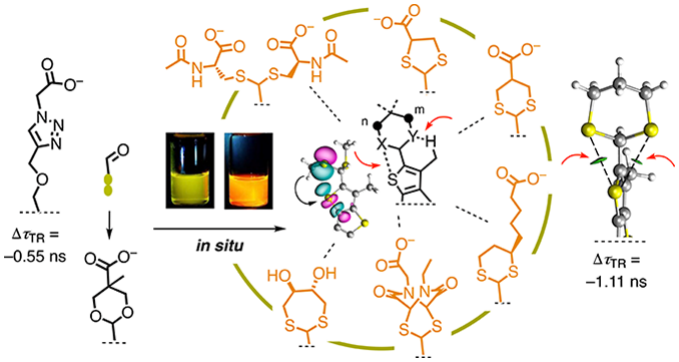

Progress with fluorescent flippers, small-molecule probes to image membrane tension in living systems, has been limited by the effort needed to synthesize the twisted push–pull mechanophore. Here, we move to a higher oxidation level to introduce a new design paradigm that allows the screening of flipper probes rapidly, at best *in situ*. Late-stage clicking of thioacetals and acetals allows simultaneous attachment of targeting units and interfacers and exploration of the critical chalcogen-bonding donor at the same time. Initial studies focus on plasma membrane targeting and develop the chemical space of acetals and thioacetals, from acyclic amino acids to cyclic 1,3-heterocycles covering dioxanes as well as dithiolanes, dithianes, and dithiepanes, derived also from classics in biology like cysteine, lipoic acid, asparagusic acid, DTT, and epidithiodiketopiperazines. From the functional point of view, the sensitivity of membrane tension imaging in living cells could be doubled, with lifetime differences in FLIM images increasing from 0.55 to 1.11 ns. From a theoretical point of view, the complexity of mechanically coupled chalcogen bonding is explored, revealing, among others, intriguing bifurcated chalcogen bonds.

## Introduction

Thio/acetals are popular functional groups in fluorescent probes. Thioacetals have been used for mercury sensing,^1−6^ quenching,^7−11^ and the detection of reactive oxygen species,^12−15^ while acetals have been used to sense pH changes,^16−20^ detect metal ions,^21−23^ visualize and modulate enzyme activity,^24−27^ and for photochromic switching^28−31^ and super-resolution microscopy.^32^ Usually, the conversion of aldehydes into thio/acetals is designed to turn off fluorescence, or *vice versa*, for different reasons. In this study, thio/acetalization is designed to turn on, rather than turn off, the fluorescence of flipper probes easily, at best *in situ*, to simultaneously install variable targeting groups and screen for new chalcogen-bonding donors.

**Figure 1:**
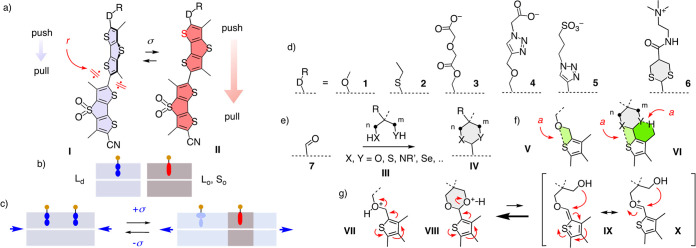
(a) Flipper design as planarizable push–pull probes (*r*, repulsion; σ, tension), responding to increasing membrane (b) order and (c) tension with increasing red-shift, lifetime, and intensity. (d) Realized donors D and targeting units R (**1**–**5**) and new junctions (e.g., **6**). (e) Simultaneously installing D (X, Y) and R *in situ* (**III**, **IV**) to (f) screen for chalcogen-bonding donors X (Y) and support from CH···Y (X) bonds (**V**, **VI**, *a*, attraction) and to (g) prevent spontaneous elimination (**VII**) with tethered leaving groups (**VIII**–**X**).

Fluorescent flippers have been introduced as small-molecule fluorescent probes to image membrane tension in living systems (Figure 1a–c).^33^ For the imaging of biomembrane function,^34−47^ membrane tension^48−50^ is particularly interesting but also particularly demanding because physical forces are detectable only through the suprastructural changes they cause.^33^ Inspired by nature,^51^ we have considered the concept of planarizable push–pull probes to tackle this challenge.^52^ The resulting flipper probes are built around two dithienothiophenes,^53,54^ one electron rich and one electron poor. In the relaxed ground state **I**, they are twisted out of coplanarity by chalcogen-bonding repulsion (Figure 1a). Planarization by mechanical compression in the ground state brings donors and acceptors into conjugation. The resulting push–pull system causes a large red-shift of the absorption maximum and increases fluorescence lifetime, intensity, and quantum yield. This mode of action is contrary to other membrane probes that report off equilibrium in the excited state.^34−47^ In lipid bilayer membranes, these changes report on an increasing order from liquid-disordered (L_d_) to liquid-ordered (L_o_) and solid-ordered (S_o_) membranes (Figure 1b). Tension applied to biomembranes increases fluorescence lifetimes because the probe response is dominated by membrane reorganization, particularly tension-induced phase separation (Figure 1c).

Progress with flipper probes has been limited by the synthetic effort required for their preparation. Particularly challenging is the donor terminus. Located at the membrane surface, variable targeting units R have to be attached to this terminus (Figure 1).^33,55−57^ Moreover, donors D are needed in planarized flippers **II** to generate a strong push–pull system accounting for large red-shifts, but they are incompatible with twisted flippers **I** because the decoupled donor side oxidizes.^33^ Flippers such as **1** with simple alkoxy donors are not stable for this reason (Figure 1d). Sulfide donors, like in **2**, that turn on only when attached to electron-deficient aromatics obtained by the planarization of flippers, did not afford the spectroscopic properties needed for bioimaging. The first functional flipper **3** contains a thenyl ester as a donor and a carboxylate to target plasma membranes. However, flipper **3** was unstable because the thenyl esters and even ethers were easily eliminated (Figure 1g). This problem was not solved but suppressed in flipper **4** by a proximal triazole as a proton scavenger, which works well as a tension reporter (TR) in cells and has been made available for the community as Flipper-TR.

Deletion of the fragile thenyl ether and direct use of triazole as donor, as in **5**, increased stability, but the added aromatic system perturbed spectroscopic properties.^54,57^ Both thenyl ether and triazole have been proposed to act as noncovalent turn-on donors, forming a chalcogen bond^58−63^ as soon as probe planarization deepens the σ hole on the endocyclic sulfur (Figure 1f). Considering the decisive importance of these hypothetical chalcogen-bonding turn-on donors^64^ on the one hand and the limitations of thenyl ethers with regard to stability and variability on the other hand, we decided to move one oxidation level higher and explore thio/acetals as clickable donor junctions in fluorescent flipper probes as exemplified in **6**. They are shown to provide facile access to rich chemical space, intriguing chalcogen-bonding donor motifs, and more than doubled sensitivity for the imaging of membrane tension in living cells.

## Results and Discussion

### Design

Thio/acetal donor junctions could be installed by late-stage modification of common aldehyde precursor **7** with the respective thiols and alcohols **III** (Figure 1e). Saturated 1,3-heterocycles **IV** (Figure 1e) were particularly inviting because classics like cysteines, lipoic acid, asparagusic acid,^65,66^ DTT,^67,68^ epidithiodiketopiperazines (ETPs),^69^ and so on promise access to rich chemical space. This flipper diversity will allow for the screening for improvements of the original chalcogen-bonding donors **V** together with an additional, less powerful noncovalent CH···X bonding donor for a refined push–pull system **VI** (Figure 1f). Moreover, saturated 1,3-heterocycles **IV** were expected to prevent the thenyl ether elimination **VII** by intramolecular tethering of the leaving group to enable reverse ring closure (Figure 1g, **VIII**–**X**). Overall, donor junctions could thus be expected to secure facile synthetic access to a broad variety of turn-on donors with attached targeting units of free choice, all in one single final step, at best possible *in situ*.

### Molecular Modeling

**Figure 2:**
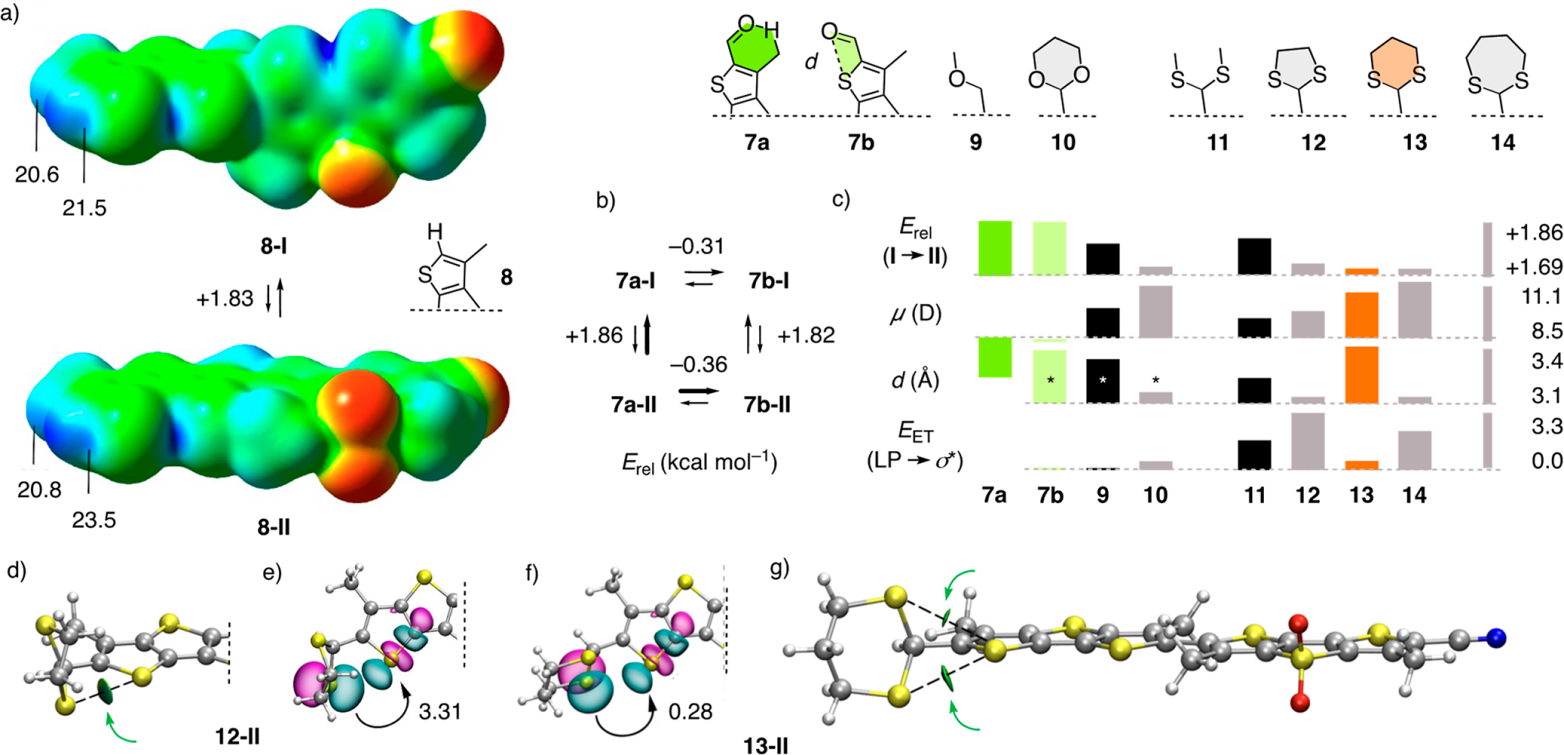
(a) MEP surface of **8-I** (90° twisted) and **8-II** (planar), with positive maxima (blue) accounting for chalcogen and CH···X bonding (isodensity 0.001 au). (b) Computed data for cascade switching with **7** and (c) for the planar conformer **II** of **7**, **9**-**14**, covering energy *E*_rel_ compared to twisted conformer **I**, macrodipole μ, chalcogen-bonding S–X (O, S) distance *d* (*: 2.8–3.0 Å), and LP-σ* NBO electron-transfer stabilization energy *E*_ET_. (d, g) NCIplot analysis with isosurfaces in green (arrows) and (e, f) NBOs involved in chalcogen bonding for the planar conformer **II** of (d, e) **12** and (f, g) **13**, with indication of *E*_ET_. All energies are in kcal mol^–1^.

Computational evaluation of potential chalcogen-bonding donors was performed at the PBE0-D3/def2-TZVP level of theory. The virtual flipper **8** was of interest *in silico* because the view on the relevant σ hole is not obstructed by chalcogen bonds. Upon planarization, the maximum of the molecular electrostatic potential (MEP) surface on the sulfur increased from *E*_pot_ = 21.5 to *E*_pot_ = 23.5 kcal mol^–1^, while the C–H donor increased only by *E*_pot_ = +0.2 kcal mol^–1^ (Figure 2a). The conclusion that chalcogen-bonding donors turn on upon planarization was confirmed by the switching cycle^64^ of the pseudo-pull–pull flipper **7** with a misplaced aldehyde acceptor in place of the exocyclic push–pull donor of flipper probes. A macrodipole doubling from μ = 3.2 D for the planar conformer **7a-II** to μ = 6.5 D for **7b-II** showed how chalcogen bonding in **7b-II** but not CH···O bonding in **7a-II** weakens the aldehyde acceptor by reinjection of withdrawn electron density (Figure 2c and Table 1, entries 1, 2). The twice as large push–pull dipole stabilized **7b-II** by *E*_rel_ = −0.36 kcal mol^–1^ compared to **7a-II**, while the twisted **7b-I** with weaker dipole and chalcogen bonds was only *E*_rel_ = −0.31 kcal mol^–1^ more stable than **7a-I** (Figure 2b). The uphill planarization of the twisted **7b-I** (*E*_rel_ = +1.86 kcal mol^–1^) was also facilitated by the doubled push–pull dipole of **7b-II** (*E*_rel_ = +1.82 kcal mol^–1^).

**Table 1:** Computational Data for the Flipper Probes

entry	Cps^a^	*E*_rel_ (kcal mol^–1^)^b^	μ (D)^c^	*d* (Å)^d^	*E*_ET_ (kcal mol^–1^)^e^
1	**7a**	+1.86	3.2		
2	**7b**	+1.82	6.5	2.99	<0.05
3	**9**	+1.75	9.9	2.97	<0.05
4	**10**	+1.69	10.9	2.80	0.14
5	**11**	+1.78	9.2	3.23	1.53
6	**12**	+1.70	9.4	3.13	3.31
7	**13**	+1.69	10.5	3.39, 3.46	0.28
8	**14**	+1.69	11.1	3.14	2.33

a Compounds, see Figure 2.

b Relative energy cost for planarization from conformer **I** (90° torsion angle) to conformer **II** (180°, Figure 1a).

c Macrodipole of planar conformer **II**.

d Chalcogen-bonding distance S–X (X = O, S) in planar conformer **II**.

e NBO electron-transfer component of chalcogen bond in conformer **II**.

Conversion of aldehyde **7** into minimalist thenyl ether **9** and acetal **10** increased the push–pull dipole from μ = 6.5 D over μ = 9.9 to μ = 10.9 D, which in turn stabilized planar high-energy conformer **II** from *E*_rel_ = +1.82 kcal mol^–1^ over *E*_rel_ = +1.75 kcal mol^–1^ to *E*_rel_ = +1.69 kcal mol^–1^ relative to the twisted conformer **I** (Figure 2c and Table 1, entries 2–4). The origin of these changes from stronger chalcogen-bonding donors was supported by decreasing S–O distances from *d* = 2.99 Å for **7a** over *d* = 2.97 Å for **9** to *d* = 2.80 Å for **10**, all much shorter than the sum of the van der Waals (vdW) radii (*d* = 3.32 Å). Moreover, NBO analysis indicated that electron transfer from the oxygen lone pair (LP) to the antibonding σ* orbital of the sulfur starts to contribute significantly to chalcogen bonding only with the 1,3-dioxane donor **10**.

In the sulfur series, dipole and stabilization of planar conformer **II** increased from acyclic to cyclic thioacetals. With cyclic thioacetals, dipoles increased with the ring size from μ = 9.4 D for 1,3-dithiolane **12** over μ = 10.5 D for dithiane **13** to record μ = 11.1 D for dithiepane **14** (Figure 2c and Table 1, entries 6–8). This was reflected in the chalcogen bond length decreasing from acyclic **11** with *d* = 3.23 Å to cyclic **12** and **14** with *d* ∼ 3.14 Å, all well below vdW radii of *d* = 3.60 Å (Figure 2c and Table 1, entries 5, 6, 8). The longest chalcogen bond *d* = 3.38 Å of **13** coincided with the weakest NBO electron transfer contribution of *E*_ET_ = 0.28 kcal mol^–1^, while the push–pull dipole remained very high at μ = 10.5 D and planarization with *E*_rel_ = +1.69 kcal mol^–1^ was least disfavored (Figure 2c and Table 1, entry 7). This apparent contradiction could be understood with the highest conformational rigidity of the 1,3-dithiane chair, which positions both sulfur atoms at almost equal distance for a formal bifurcated^70−72^ three-center chalcogen bond,^73^ where long distances are compensated by doubled interactions (*d*_1_ = 3.39 Å and *d*_2_ = 3.46 Å, Figure 2f,g). The existence and attractive nature of a bifurcated chalcogen bond were confirmed by NCIplot analysis of **13**, which showed the presence of two reduced density gradient disk shape green isosurfaces (Figure 2g).

This unorthodox bifurcated chalcogen bond occurred with dithiane **13** but not with the ring-contracted dithiolane **12**, the ring-expanded dithiepane **14**, and dioxane **10** with oxygen instead of sulfur donors in the chair. In dithiolane **12**, the second heteroatom was oriented toward the CH···X bond acceptor, allowing the chalcogen-bonding heteroatom to position best for minimal bond length (*d* = 3.13 Å) and maximal NBO electron transfer (*E*_ET_ = 3.31 kcal mol^–1^, Figure 2c–e and Table 1, entry 6). The same was true in the oxygen series with dioxane **10**, with the NBO electron transfer being naturally smaller (Figure 2c and Table 1, entry 4). Increasing conformational flexibility in dithiepane **14** presumably accounted for the best balance of all parameters, resulting in a record dipole together with a short, nonbifurcated chalcogen bond and substantial NBO electron transfer (*E*_ET_ = 2.33 kcal mol^–1^, Figure 2c and Table 1, entry 8).

Almost equal stabilization (*E*_rel_ = +1.69 kcal mol^–1^) and polarization (μ = 10.5 D) of planar high-energy conformer **13** compared to ring-contracted **12** implied that unorthodox bifurcated chalcogen bonds can be at least as powerful as optimized conventional chalcogen single bonds. The significance of these bifurcated chalcogen bonds could only be identified and appreciated in the context of the coupled processes in planarizable push–pull probes. They will be of interest in future design strategies in general.

The overall trends identified by molecular modeling reflect the complexity of the system. Also, without inclusion of charge transfer and excited-state structures, they should thus be considered with due caution in interpreting flipper performance.

### *In Situ* Thioacetalization

**Figure 3:**
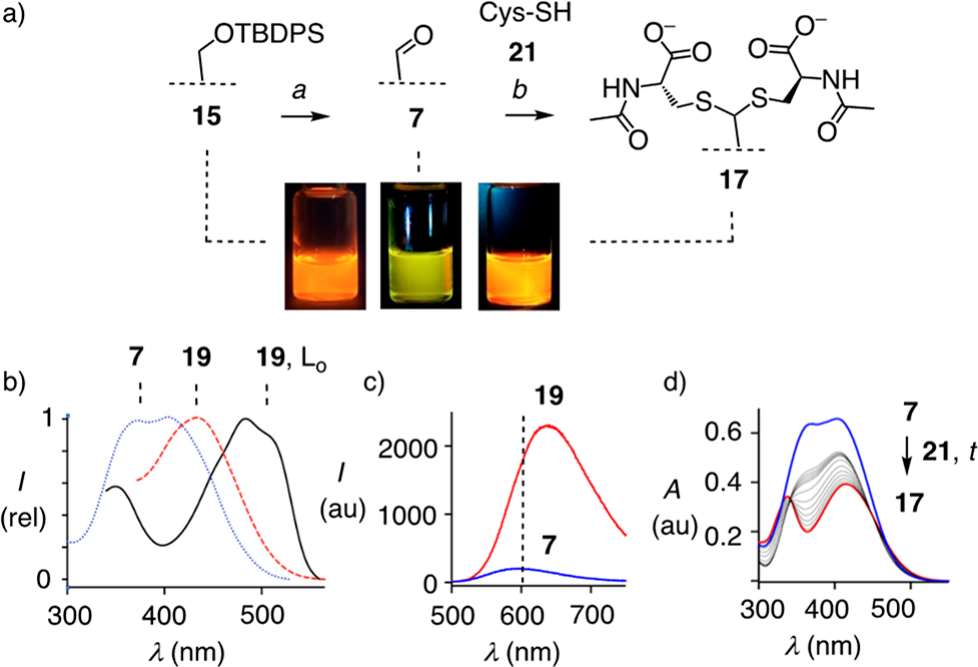
(a) Fluorescence change during the synthesis of aldehyde **7** from **15** and the conversion into thioacetal **17** (λ_ex_ = 365 nm, CH_2_Cl_2_; *a*, 1. DDQ, CH_2_Cl_2_/H_2_O, 2 h, rt, *b*, **21**, 4 M HCl in dioxane, CH_2_Cl_2_, 40 min, rt. (b) Normalized excitation spectra of **7** (blue) and **19** (red) in chloroform and **19** in SM/CL 7:3 LUVs (black, λ_em_ = 630 nm). (c) Not normalized emission spectra of **7** (blue, λ_ex_ = 412 nm) and **19** (red, λ_ex_ = 420 nm) in DMSO. (d) Absorption spectra of **7** (180 μM) in CH_2_Cl_2_ with 4 M HCl in dioxane with time after addition of 100 equiv of Ac-Cys **21** (0 (blue), 5–60 (black), and 80 min (red)).

**Figure 4:**
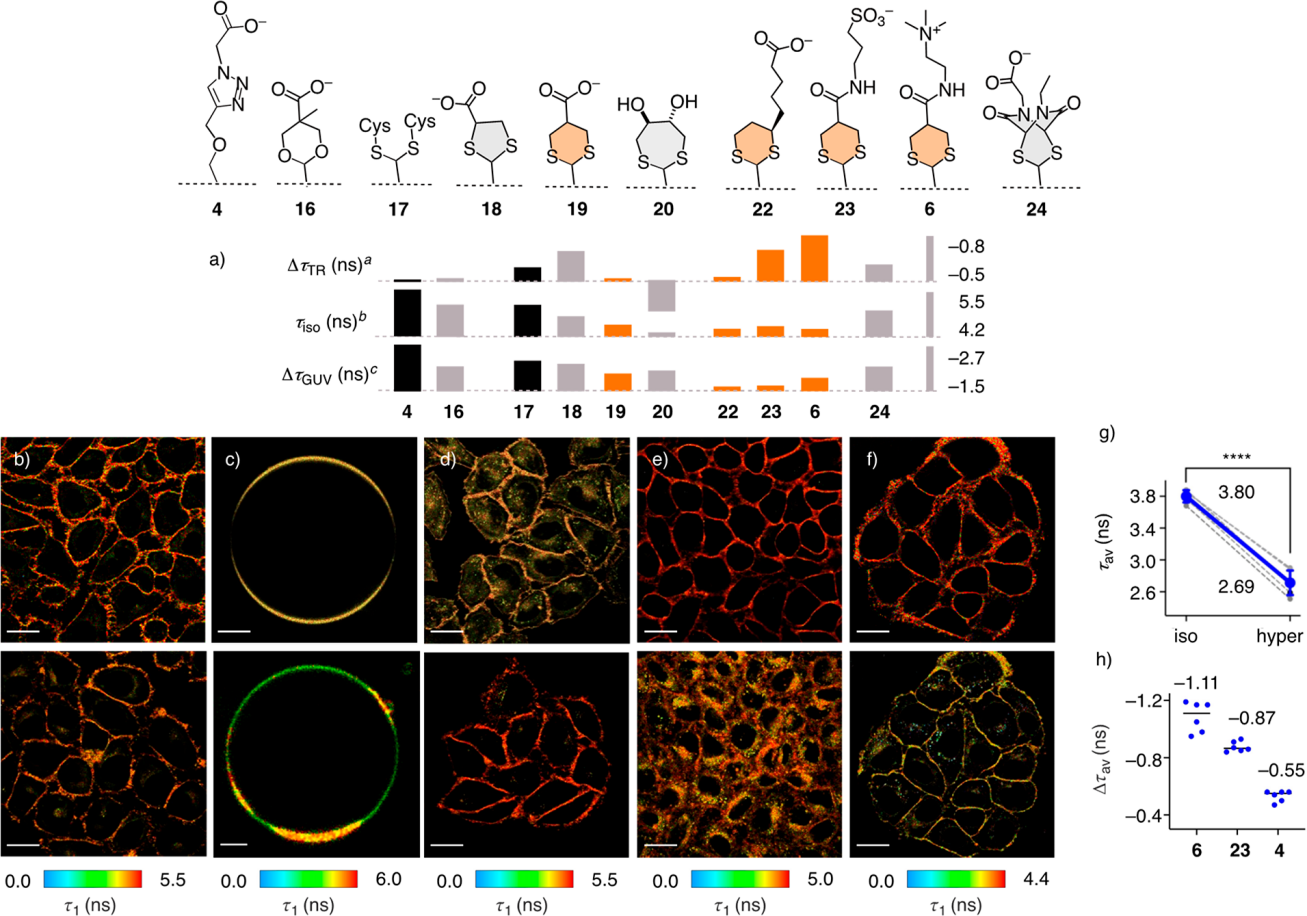
(a) Experimental data for thio/acetal flippers **6**, **16**–**20**, and **22**–**24** compared to original **4**, covering ^*a*^difference in fluorescence lifetime τ_1_ between isoosmotic and hyperosmotic HK cells, ^*b*^fluorescence lifetime τ_1_ in plasma membrane of isoosmotic HK cells, and ^*c*^difference in fluorescence lifetime τ_1_ between L_o_ SM/CL 7:3 GUVs and L_d_ DOPC GUVs; fully charged structures shown, without counterions. (b–h) FLIM images of (b) HK cells labeled with pure (top) and *in situ* prepared **19** (bottom), (c) GUVs composed of L_o_ SM/CL 7:3 (top) and L_o_ + L_d_ DOPC/SM/CL 58:25:17 with **19**, (d) HK cells with **20** (top) and **17** (bottom), and with (e) **18** and (f) **6** before (top) and after hyperosmotic shock (bottom, **18**: 0.5 M sucrose, **6**: 0.25 M sucrose). Scale bars: cells, 20 μm; GUVs, 3 μm. (g) Analysis of τ_av_ changes in FLIM images of **6** in the plasma membrane of HK cells under isoosmotic (long) or hyperosmotic (short τ_av_, 0.5 M sucrose) conditions; 1 circle = 1 measurement; solid thick blue line, mean value; whiskers, standard deviation; dashed thin gray lines, measurements with the same cell; statistical significance determined with the one tailed paired students *t* test, ****p < 0.0001. (h) Lifetime changes of **4**, **6** and **23** in HK cells with hyperosmotic shock (0.5 M sucrose), reporting Δτ_av_ instead of Δτ_TR_ (= Δτ_1_).

Aldehyde **7** was obtained from previously reported ether **15** by simultaneous deprotection and oxidation with DDQ (Figure 3a and Scheme S1). Flipper **15** was synthesized in 12 steps from commercially available starting materials following reported procedures.^33^ Weakening of the push–pull system in **15** by the aldehyde acceptor in **7** was correctly reflected by the emission changing from orange to green. As initial targets for donor junctions, 1,3-dioxolane **16** and thioacetals **17**–**20** were selected to probe for accessibility of the central motifs from popular starting materials like reduced asparagusic acid^65,66^ or dithiothreitol^67,68^ (DTT, Figures 3 and 4a). Thio/acetalization of the pseudo-pull−pull fluorophore **7** was visible by the naked eye by a change from green back to orange fluorescence (Figure 3a). As expected from the installation of a push–pull system, this red-shift coincided with a de facto turn-on increase in fluorescence (Figure 3c).

In the twisted form **I** of substrate and product in solution, changes in the absorption and excitation spectra were naturally less spectacular. The spectrum of substrate **7** in chloroform showed two maxima at 367 and 406 nm (Figure 3b, blue). Upon thioacetalization, the two maxima moved apart to longer and shorter wavelengths (Figure 3b,d, red). Direct detection of thioacetalization was realized in the absorption spectra (Figure 3d). The formation of the acyclic thioacetal **17** occurred with a *t*_50_ ∼ 6 min (Figure S10). The *in situ* synthesis of 1,3-dithiolane **18** showed similar kinetics, while the formation of dithiane **19** was faster (*t*_50_ ∼ 2 min) and dithiepane **20** formed instantaneously (*t*_50_ < 2 min). Thioacetal formation was confirmed by LC-MS (Figures S11–S16) and NMR analyses, performed after purification of some compounds. Added to HeLa Kyoto (HK) cells with or without short work-up, *in situ* produced thioacetals were not toxic and afforded FLIM images that were indistinguishable from images obtained from isolated and purified flippers (Figure 4b).

Thioacetal flipper **19** was stable in buffer at pH = 7 and pH = 5 for more than 15 h (Figure S22a,b). The complementary acetal flipper **16** showed identical stability at pH = 7 but hydrolyzed at pH = 5 with *t*_50_ = 4 h (Figure S22c,d). Usually, the replacement of oxygen by sulfur in functional groups decreases stability and enables dynamic covalent chemistry because the larger size gives weaker bonds, stronger acids, and better leaving groups. The exception with thioacetals, long known, originates from the different mechanism at work, with C=S^+^ intermediates that are harder to access than C=O^+^ intermediates, for the same reason.

The spectroscopic properties in L_d_ LUVs (large unilamellar vesicles) of all new flippers were roughly the same as for the original Flipper-TR **4**, characterized by a broad excitation maximum at λ_ex_ ∼ 440 nm (Figure S17). In L_o_ LUVs, the excitation maxima red-shifted around +80 nm to a 0–0 transition at λ_ex_ ∼ 520 nm, with distinct differences between the different flippers, particularly with regard to vibrational fine structure (*vide infra*, Figures 3b and S18–S21). These results indicated that, remarkably, all new donor junctions produced operational, mechanosensitive flippers.

### Fluorescence Lifetime Imaging Microscopy

The full flipper collection was analyzed by FLIM (fluorescence lifetime imaging microscopy) of GUVs (giant unilamellar vesicles) and HK cells (Figures 4 and S23–S29, Table 2). Lifetimes τ_av_ or τ_1_ for the longer component were extracted from FLIM images by biexponential fitting of decay curves. Reported from GUVs are τ_1_ in L_o_ membranes, termed τ_Lo_ (SM/CL 7:3, Figure 4c, top), their difference Δτ_GUV_ to τ_1_ in L_d_ membranes (DOPC), and τ_Lo_^m^, τ_1_ in L_o_ domains of mixed membranes containing L_o_ and L_d_ domains (DOPC/SM/CL 58:25:17, Figure 4c, bottom, and 4a, Table 2). From HK cells, reported are τ_iso_ for τ_1_ of plasma membranes under isoosmotic conditions (Figures 4a and 4e,f, top) and their difference Δτ_TR_ to τ_1_ under hyperosmotic conditions (Figures 4e,f, bottom, and 4a, Table 2).

**Table 2:** Fluorescence Lifetimes of Flipper Probes

entry	Cps^a^	τ_Lo_ (ns)^b^	Δτ_GUV_ (ns)^c^	τ_Lo_^m^ (ns)^d^	τ_iso_ (ns)^e^	Δτ_TR_ (ns)^f^
1	**4**	6.0	–2.7	5.5	5.5	–0.5
2	**16**	5.7	–2.1	4.9	5.1	–0.5
3	**17**	5.4	–2.3	5.2	5.1	–0.6
4	**18**	5.4	–2.2	4.8	4.7	–0.7
5	**19**	5.2	–1.9	4.9	4.5	–0.5
6	**20**	5.1	–2.0	4.6	4.3	–0.2
7	**22**	4.9	–1.6	4.4	4.4	–0.5
8	**23**	5.0	–1.6	4.1	4.5	–0.7
9	**6**	5.2	–1.8	4.2	4.4	–0.8
10	**24**	5.4	–2.1	4.9	4.9	–0.6

a Compounds, see Figures 1 and 4.

b Fluorescence lifetime τ_1_ in L_o_ SM/CL 7:3 GUVs.

c Difference in fluorescence lifetime τ_1_ between L_o_ SM/CL 7:3 GUVs and L_d_ DOPC GUVs.

d Fluorescence lifetime τ_1_ in L_o_ domains of mixed L_o_ + L_d_ DOPC/SM/CL 58:25:17 GUVs.

e Fluorescence lifetime τ_1_ in plasma membrane of isoosmotic HK cells.

f Difference in fluorescence lifetime τ_1_ between isoosmotic and hyperosmotic HK cells.

Within the series **16**–**20**, selected to implement computational guidelines with readily accessible reagents, fluorescence lifetimes were generally higher for S–O chalcogen bonds, that is, ether **4** and acetal **16**, than for thioacetals **17**–**20** (Figure 4a and Table 2, entries 1–6). In the thioacetal series, lifetimes generally decreased with an increasing flipper macrodipole. This trend was presumably caused by increasing flipper mispositioning rather than differences in chalcogen bonding. Under isoosmotic conditions, the selectivity of plasma membrane labeling was best for the most hydrophilic, dianionic acyclic thioacetal **17** (Figure 4d, bottom). The worst selectivity coincided with the shortest lifetimes for flipper **20** with a DTT dithiepane junction that misses the charge for plasma membrane targeting (Figure 4d, top). Plasma membrane labeling with the intermediate anionic dithiolane **18** (Figure 4e, top) and dithiane **19** (Figure 4b) was good under isoosmotic conditions. However, under hyperosmotic conditions, all thioacetal flippers were internalized rapidly (Figures 4e, bottom, and S29), demonstrating that there is much room for improvement for membrane targeting, which should improve fluorescent lifetimes at the same time.

To image changes in membrane tension, large differences in lifetime under iso- and hyperosmotic conditions in HK cells are most important. Together with differences in intensity, this Δτ_TR_ defines the responsiveness to changes in membrane tension. Under the present conditions, the original Flipper-TR **4** had Δτ_TR_ = −0.5 ns (Figure 4a and Table 2, entry 1). With acetal **16**, this did not change, but thioacetals **17** and **18** had higher sensitivity up to Δτ_TR_ = −0.7 ns, while the poor interfacing of dithiepane **20** was not only reflected by internalization and lowest τ_iso_ = 4.3 ns but also in a drop of sensitivity down to less relevant Δτ_TR_ = −0.2 ns (Figure 4a and Table 2, entries 2–4, 6). Within the structurally comparable dithiolane **18** and dithiane **19**, stronger conventional chalcogen bonding (**12**, **13**, Figure 2c) increased the sensitivity of tension imaging from Δτ_TR_ = −0.5 to Δτ_TR_ = −0.7 ns (Figure 4a and Table 2, entries 4, 5).

New records in sensitivity despite unoptimized interfacing implied much room to improve on thioacetal flippers. Dithiane **19** from asparagusic acid was selected as the starting point, also to assess the potential of the unusual bifurcated chalcogen bonds (Figure 2f). Dithiane flipper **22** obtained from reduced lipoic acid did not improve Δτ_TR_ = −0.5 ns, presumably because the longer linker is too hydrophobic to match the membrane interface well (Figure 4a and Table 2, entry 7).

The specifically designed dithiane flippers **23** and particularly **6** with negative and positive charges placed at a distance as in the original Flipper-TR **4** and with better matching linkers increased sensitivity to tension changes up to Δτ_TR_ = −0.8, counted exclusively for the plasma membrane (Figure 4a and Table 2, entries 8, 9). These maximized Δτ_TR_ sensitivity coincided with nearly suppressed internalization also under hyperosmotic conditions (Figure 4f vs 4e, bottom).

Determination of tension sensitivity Δτ_TR_ from alternative τ_av_ gave even larger differences (Figure 4g,h). Compared to the original Flipper-TR **4** at Δτ_av_ = −0.55 ± 0.04 ns, already anionic dithiane flipper **23** increased to Δτ_av_ = −0.87 ± 0.03 ns. With the cationic dithiane flipper **6**, the sensitivity of membrane tension imaging in living cells doubled beyond 1 ns, i.e., Δτ_av_ = −1.11 ± 0.11 ns (Figure 4h). The significant increases in responsiveness from **19** over **23** to **6** were obtained with the same mechanophore. Therefore, they originated from interactions with the surrounding membranes. An ongoing systematic study suggests that in deconvoluted vibrational fine structures of excitation spectra, an intensity ratio of the second, formally 0–1 transition divided by the third transition of *I*_1/2_ > 1 is indicative of excellent matching and partitioning into ordered domains (unpublished). In agreement with this emerging understanding, *I*_1/2_ values did indeed increase significantly with increasing responsiveness from dithianes **19** to **23** and **6** (Figure S20; to some extent, also S_o_/L_d_ intensity ratios, Figure S19).

Increased sensitivity to image membrane tension with dithianes was particularly impressive because this is perhaps the most intriguing but presumably not the most promising chalcogen-bonding donor junction, and only three analogues were tested to improve (Figure 4a). To illustrate the vast chemical space accessible, ETPs^66,68,69^ were considered as clickable donor junctions. This natural-product-derived motif yields disulfides at maximal tension that are of interest to penetrate cells.^66,68,69^ The parent ETP was reduced prior to *in situ* thioacetalization with aldehyde 7 (Scheme S9). The resulting thioacetal junction **24** features a seven-membered ring like the dysfunctional dithiepane **20** but performed much better, more like dithiolane **17**, including high Δτ_TR_ = −0.6 at similarly impressive τ_Lo_, τ_Lo_^m^, τ_iso_, and Δτ_GUV_ (Figure 4a and Table 2).

## Conclusions

This study introduces a new strategy to facilitate synthetic access to fluorescent flipper probes. The solution of long-standing problems is found by moving one oxidation level higher, from ethers to thio/acetals. Clickable chalcogen-bonding donor junctions enable late-stage modifications to screen for noncovalent donors and targeting units in one step, at best *in situ*. Computational exploration of the opened structural space is attractive to dissect different modes of coupled chalcogen bonding including intriguing bifurcated chalcogen bonds. All of the explored acetal and thioacetal junctions provided operational tension probes. Fluorescence properties in cells are overall dominated by interfacing with the cellular environment rather than the nature of the chalcogen-bonding donor. Already introductory examples for late-stage screening of donor junctions suffice to provide fluorescent probes that double the sensitivity to changes in membrane tension in living cells.

Based on these results, *in situ* thioacetalization will be of practical use to easily access intracellular targeting in the broadest sense. However, the most promising is the disclosed access to a rich structural space on a new oxidation level. Late-stage clicking is not limited to thio/acetals, which promises access to intriguing chalcogen-bonding donor motifs and more (Figure 1e). While nitrogen or selenium may be less attractive in this context (quenching, acidity, etc.), perspectives with sulfur and oxygen beyond ring contraction and expansion, interfacing, and targeting include catechols and thiocatechols, for instance, or the integration into larger systems, from oligosaccharide to peptide chemistry, including α-helix stapling. These perspectives are valid and inspiring for fluorescent probes in general.

## Methods

### Thioacetal Flippers Made *In Situ*

Flippers **17**, **18**, **19**, and **20** were prepared *in situ* by adding HCl (24 μL of 4 M in dioxane, 96 μmol) and a solution of the respective dithiol/thiol in DMF (6 μL of 1 M, 6 μmol, *N*-acetyl-l-Cys **21**, 2,3-dimercaptopropanoic acid, reduced asparagusic acid, DTT) to a solution of **7** (300 μL of 0.2 mM, 0.06 μmol) in CH_2_Cl_2_ at 25 °C (Scheme S8). Absorption spectra (*l* = 0.1 cm) of the reaction mixture were recorded every 1–5 min, and the corresponding mixture without **7** was used as the background (Figures 3d and S9). Based on the time-dependent absorption spectra, the kinetics of conversion from **7** to **17**, **18**, **19**, and **20** were determined (Figure S10). The complete consumption of **7** and the generation of the desired products were confirmed by LC-MS of the reaction mixtures (Figures S11–S14). For direct use in FLIM imaging of GUVs and living cells, the obtained product mixtures were extracted with brine and CH_2_Cl_2_, and the organic phase was dried over Na_2_SO_4_ and filtered through a cotton plug in a pipet. The filtrate was concentrated, and the residue was dissolved in DMSO (0.5 mL for **17**, **19**, and **20** and 1.0 mL for **18**).

For flipper **24**, solutions of the parent ETP^69^ (1 M, 30 μL) and tris(hydroxypropyl)phosphine (THPP, 1 M, 60 μL) in DMF were combined and stirred for 10 min (Scheme S9). The resulting solution of reduced ETP and HCl (4 M, 120 μL in dioxane) was added to a solution of **7** (1.0 mM, 300 μL) in CH_2_Cl_2_. LC-MS confirmed the formation of **24***in situ* (Figure S15) and showed the presence of residual **7** (Figure S16). For direct use in the FLIM imaging of GUVs and living cells, the reaction mixture was stirred for 48 h. Then, the product mixture was extracted with brine and CH_2_Cl_2_, dried over Na_2_SO_4_, filtered through a cotton plug in a pipet, concentrated *in vacuo*, and dissolved in DMSO (0.5 mL).

### FLIM Imaging of Thioacetal Flippers Made *In Situ*

For FLIM imaging of GUVs, 10 μL of stock solutions of GUVs and 0.2 to 0.4 μL of stock solutions of flippers in DMSO (0.6 mM **17**, **19**, **20**, 0.3 mM **18**, 0.5 mM **24**) were added to 190 μL of Tris buffer (10 mM Tris/Tris·HCl, 100 mM NaCl, pH 7.4). The obtained suspensions were placed on a 35 mm glass bottom dish (Mattek Corporation, P35G-1.5-14-C) and left for 15 min at room temperature before imaging (Figure S25). Leica Application Suite Software LASX FLIM 4.5.0 Stellaris or SymPhoTime 64 software from PicoQuant was used to analyze the FLIM images. The lifetimes τ_1_ were calculated from a biexponential fit of the signal coming from GUVs (selected as the ROI by “painting”).

For FLIM measurements in HeLa Kyoto cells, the cells (8 × 10^4^ cells mL^–1^) were seeded in FluoroBrite DMEM (high d-glucose, without phenol red) medium containing 10% fetal bovine serum (FBS), 1% penicillin/streptomycin (PS), and 1% glutamine and kept at 37 °C at 5% CO_2_ overnight. Then, the cells were washed (3 × 1 mL) with PBS buffer and incubated with DMEM medium containing the corresponding probe (0.6 μM for **18**, 1 μM for other probes, 1 mL) for 10 min at 37 °C at 5% CO_2_. The images were acquired without exchanging the incubation medium or additional washing. The hypertonic shock was achieved by adding 1 mL of 1 M sucrose medium containing the corresponding probe (0.6 μM for **18**, 1 μM for other probes) in the dish containing 1 mL of isotonic medium for 30 min (Figures 4e and S29). FLIM data were analyzed by using SymPhoTime 64 software (PicoQuant) that fit fluorescent decay data (at least 6 cells per picture, 4 cells for **23**) to a biexponential deconvolution model from the plasma membrane only.

## Supporting Information Available

Detailed experimental procedures, materials and methods, compound synthesis and characterization, thioacetalization kinetics, pH-dependent probe stability, fluorescence spectroscopy in solution and LUVs, FLIM in GUVs and HK cells, theoretical methods, Cartesian coordinates, NBO analysis, NCIplot analysis, original NMR spectra (PDF)
