# Supplementary material for: Fluorogenic In Situ Thioacetalization: Expanding the Chemical Space of Fluorescent Probes, Including Unorthodox, Bifurcated, and Mechanosensitive Chalcogen Bonds
Source: JACS Au. 2023 Aug 21;3(9):2557–65. doi: 10.1021/jacsau.3c00364 (PMC10523495; doi:10.1021/jacsau.3c00364)
Supplement: Supplementary file 1 — au3c00364_si_001.pdf [file au3c00364_si_001.pdf]

Supporting Information

Fluorogenic In Situ Thioacetalization: Expanding the Chemical Space of Fluorescent Probes, Including Unorthodox, Bifurcated and Mechanosensitive Chalcogen Bonds

Xiao-Xiao Chen,[‡] Rosa M. Gomila,[§] Juan Manuel García-Arcos,[†] Maxime Vonesch,[‡] Nerea Gonzalez-Sanchis,[‡] Aurelien Roux,[†] Antonio Frontera,[§] Naomi Sakai,[‡] and Stefan Matile*[‡]

[‡]Department of Organic Chemistry, University of Geneva, 1211 Geneva, Switzerland

[§]Departament de Química, Universitat de les Illes Balears, SP-07122 Palma de Mallorca, Spain

[†]Department of Biochemistry, University of Geneva, 1211 Geneva, Switzerland

*E-mail: stefan.matile@unige.ch

Table of Contents

1.	Materials and Methods	S3
2.	Flipper Synthesis	S5
3.	Theoretical Methods	S14
	3.1. NBO Analysis	S15
	3.2. Bifurcated Nature of the Chalcogen Bonds in Compound **13-II**	S16
	3.3. NCIplot Analysis	S16
	3.4. Rotational Profiles	S18
	3.5. Cartesian Coordinates	S22
4.	Thioacetal Flippers Made *in situ*	S34
5.	Fluorescence Spectroscopy in LUVs	S40
6.	Stability of Thio/Acetal Flippers	S46
7.	Fluorescence Lifetime Imaging Microscopy (FLIM)	S47
8.	Supporting References	S54
9.	NMR Spectra	S57

1. Materials and Methods

As in ref. S1, briefly, reagents for synthesis were purchased from Fluka, Sigma-Aldrich, TCI, and Alfa Aesar. Egg sphingomyelin (SM), 1,2-Dioleoyl-*sn*-glycero-3-phosphocholine (DOPC), 1,2-Dipalmitoyl-*sn*-glycero-3-phospho-choline (DPPC) and mini-extruder were purchased from Avanti Polar Lipids. Cholesterol (CL) was purchased from Sigma-Aldrich. Phosphate buffered saline (PBS, pH = 7.4), FluoroBrite DMEM (high D-Glucose, 3.7 g/L Sodium Bicarbonate, without phenol red) medium, Penicillin-Streptomycin, Fetal Bovine Serum, Glutamine and TrypLE Express Enzyme were obtained from Thermo Fisher Scientific. Hoechst 33342 (10 mg/mL solution in water) and propidium iodide (PI, 1.0 mg/mL solution in water) were obtained from Invitrogen by Thermo Fisher Scientific.

Analytical thin layer chromatography (TLC) and preparative thin layer chromatography (PTLC) was performed on silica gel 60 F254 (Merck, 0.2 mm) and silica gel GF (SiliCycle, 1 or 0.25 mm), respectively. Column chromatography was carried out on silica gel 60 (SilicaFlash® P60, SILICYCLE, 230-400 mesh) and basic aluminum oxide (Brockmann activity, 50-200 µm). Reverse phase flash chromatography was performed on a Biotage Isolera™ Spektra using pre-packed Biotage® SNAP Ultra C_{18} cartridges. Melting points (Mp) were measured on a Melting Point M-565 (BUCHI). IR spectra were recorded on a Perkin Elmer Spectrum 100 FT-IR spectrometer (ATR, Golden Gate) and are reported as wavenumbers v in cm^{-1} with band intensities indicated as br (broad), s (strong), m (medium), w (weak). All ^{1}H and ^{13}C NMR spectra were recorded (as indicated) on a Bruker 300 MHz, 400 MHz, or 500 MHz spectrometer at room temperature (25 °C) and are reported as chemical shifts (δ) in parts per million (ppm) relatives to TMS (δ = 0). Spin multiplicities are reported as singlet (s), doublet (d), and triplet (t) with coupling constants (J) given in Hz, or multiplet (m). Broad peaks are marked as br. ^{1}H and ^{13}C resonances were assigned with the aid of additional information from 1D and 2D NMR spectra (H,H-NOESY, H,H-COSY, DEPT 135, HSQC and

HMBC). Stereoisomeric peaks are described as, for example, *mm*/*nn*. LCMS were recorded using a Thermo Scientific Accela HPLC equipped with a Thermo C18 Hypersil GOLD column (50 × 2.1 mm, 1.9 µm particles size, 0.75 mL/min, gradient elution H_2O + 0.01% TFA / CH_3CN + 0.01% TFA 4:6 to 0:10 in 4.0 min) coupled with an LCQ Fleet three-dimensional ion trap mass spectrometer (ESI, Thermo Scientific). ESI-MS for the characterization of new compounds was performed on an ESI API 150EX and ESI-HRMS was measured on Xevo G2-S Tof (Waters). All mass data are reported as mass-per-charge ratio *m/z* (intensity in %, [assignment]).

Fluorescence measurements were performed on a FluoroMax-4 spectrofluorometer (Horiba Scientific) and Duetta spectrometer, equipped with a stirrer and temperature control. Fluorescence spectra were background subtracted and corrected with factors supplied by the manufacturer. Absorbance measurements were performed on a Jasco V-650 spectrophotometer. Fluorescence lifetime imaging microscopy (FLIM) was performed either on the Leica Stellaris FALCON, at 20 MHz, with λ_{ex} = 480 nm (white light laser) and collecting the fluorescence between 550 and 650 nm, or on a Nikon Eclipse Ti A1R microscope upgraded with a FLIM kit from PicoQuant, equipped with a laser at λ_{ex} = 489 nm at 20 MHz and a 100x oil immersion objective lens.

Abbreviations. CL: Cholesterol; DOPC: 1,2-Dioleoyl-*sn*-glycero-3-phosphocholine; DPPC: 1,2-Dipalmitoyl-*sn*-glycero-3-phosphocholine; DMEM: Dulbecco's modified eagle medium; DDQ: 2,3-Dichloro-5,6-dicyano-1,4-benzoquinone; DIPEA: *N*,*N*-Diisopropylethylamine; DTT: Dithienothiophene; EDC·HCl: *N*-(3-Dimethylaminopropyl)-*N′*-ethylcarbodiimide hydrochloride; ETPs: Epidithiodiketopiperazines; FBS: Fetal bovine serum; FLIM: Fluorescence lifetime imaging microscopy; GUVs: Giant unilamellar vesicles; HK: HeLa Kyoto; LUVs: Large unilamellar vesicles; MS: Molecular Sieves; Mp: Melting point; PS: Penicillin/Streptomycin; PBS: Phosphate-buffered saline; ROI: Region of interest; rt: Room temperature; SM: Egg sphingomyelin; THPP: Tris(hydroxypropyl)phosphine; TBA: Tetrabutylammonium.

2. Flipper Synthesis

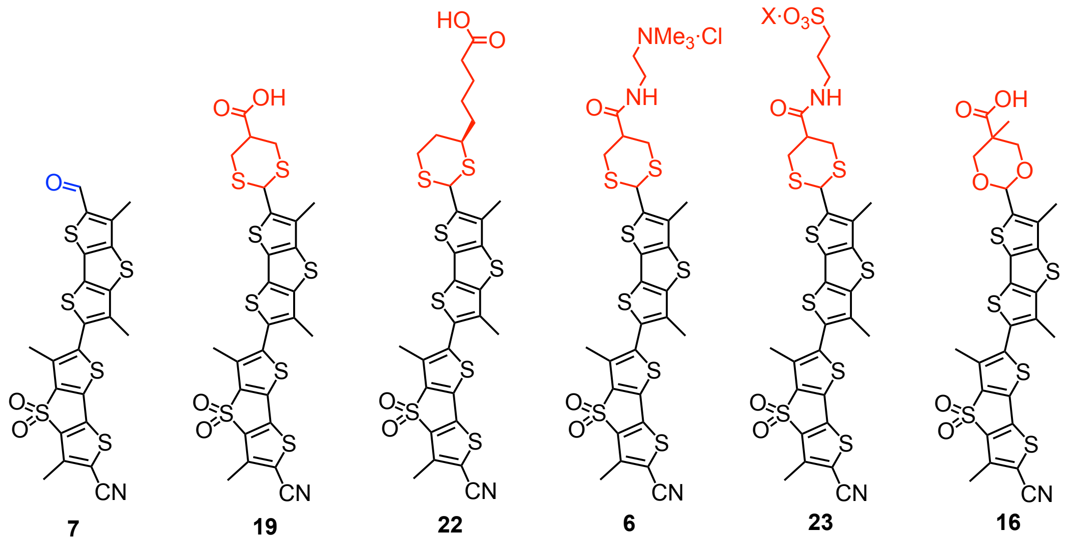

Figure S1. Structure of the flipper probes used in this study.

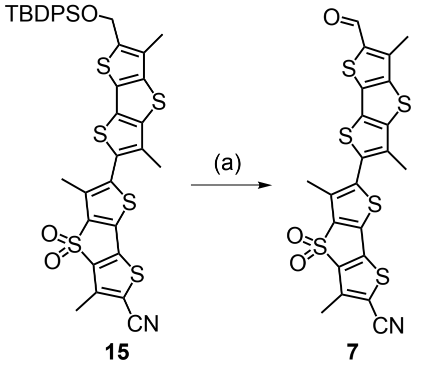

Scheme S1. (a) DDQ, CH_2Cl_2/H_2O 3:1, rt, 2 h, 87%.

Compound 15 was synthesized and purified according to the procedures reported in ref. S2 and S3.

Compound 7. To a stirred solution of **15** (201 mg, 0.260 mmol) in CH_2Cl_2/H_2O (100 mL, 3:1) was added DDQ (201 mg, 0.885 mmol). After 2 h at rt, the crude mixture was extracted with CH_2Cl_2, washed with saturated $NaHCO_3$ (3×50 mL), dried with Na_2SO_4, and concentrated *in vacuo*. The residue was purified by column chromatography (SiO_2, CH_2Cl_2) to afford **2** (120 mg, 87%) as an orange solid. R_f (CH_2Cl_2): 0.48; Mp: 338-340 °C; IR (neat): 2921 (br, w), 2212 (s, C≡N), 1644 (s,

C=O), 1410 (w), 1397 (w), 1378 (w), 1308 (s, SO_2), 1227 (s), 1139 (s, SO_2), 1089 (w), 666 (s); ^{1}H NMR (500 MHz, CD_2Cl_2): 10.12 (s, 1H), 2.73 (s, 3H), 2.59 (s, 3H), 2.41 (s, 3H), 2.38 (s, 3H); ^{13}C NMR (126 MHz, CD_2Cl_2): 182.5 (C=O), 147.0 (C), 145.6 (C), 144.0 (C), 143.9 (C), 141.8 (C), 141.3 (C), 140.4 (C), 138.8 (C), 137.4 (C), 136.4 (C), 133.6 (C), 133.2 (C), 132.7 (C), 131.1 (C), 130.0 (C), 112.8 (C), 109.8 (C), 14.2 (CH_3), 13.6 (CH_3), 13.3 (CH_3), 12.6 (CH_3); MS (ESI): 532 ($[M+H]^+$).

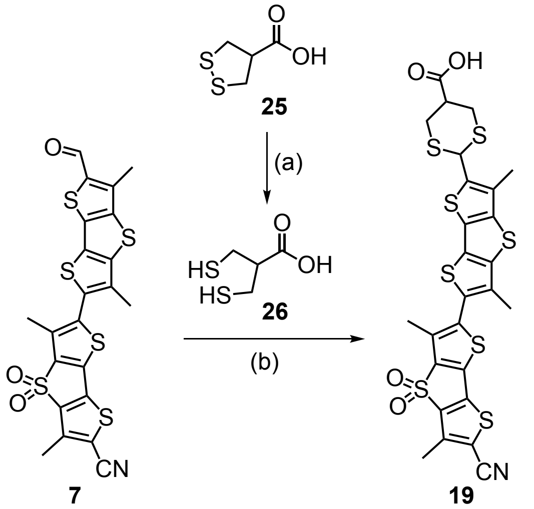

Scheme S2. (a) THPP, anhydrous THF, N_2, rt, 2 h, 66%, (b) $BF_3{\cdot}Et_2O$, anhydrous CH_2Cl_2, N_2, rt, 12 h, 83%.

Compound 25 was synthesized and purified according to the procedures reported in ref. S4 and S5.

Compound 26. To a stirred solution of **25** (60.0 mg, 0.399 mmol) in anhydrous THF (6 mL) was added THPP (83.2 mg, 0.399 mmol) under N_2 atmosphere. The reaction mixture was stirred at room temperature for 2 h. The precipitate generated in the reaction was removed by filtration, and the filtrate was concentrated *in vacuo* at 25 °C. The residue was purified by column chromatography (SiO_2, CH_2Cl_2/MeOH 20:1, R_f 0.21 with CH_2Cl_2/MeOH/CH_3COOH 10:1:0.001) to afford **26** (40.0 mg, 66%) as a colorless oil. NMR spectroscopy data are consistent with that reported in the ref. S6.

Compound 19. To a stirred solution of $BF_3{\cdot}Et_2O$ (3.5 µL, 0.028 mmol) and **7** (10.0 mg, 0.019 mmol) in anhydrous CH_2Cl_2 (8 mL), a solution of **26** (40.0 mg, 0.263 mmol) in anhydrous CH_2Cl_2 (2

mL) was added dropwise under N_2 atmosphere. The reaction mixture was stirred at room temperature for 12 h and the solvent was removed under reduced pressure. The residue was suspended in diethyl ether (3×5 mL) and the orange precipitate was collected by centrifugation to afford **19** (10.0 mg, 83%, mixture of 90:10 diastereomers) as an orange solid. IR (neat): 2919 (br, w, COOH), 2209 (m, C≡N), 1695 (s, C=O), 1417 (s), 1305 (s, SO_2), 1189 (w), 1138 (s, SO_2), 1092 (w), 1051 (w), 1014 (w), 954 (w), 755 (w), 738 (m); ^{1}H NMR (500 MHz, DMSO-d_6, mm/nn = 90:10 isomeric peaks, some isomeric peaks overlapped with solvent peak): 6.05/5.99 (s, 1H), 3.33-3.26 (2H, overlapped with H_2O peak, assigned by 2D NMR), 3.12 (dd, $^{2}J_{H\text{-}H}$ = 14.1, $^{3}J_{H\text{-}H}$ = 2.8 Hz, 2H), 2.74 – 2.69 (m, 1H), 2.49 (s, 3H, overlapped with DMSO peak, assigned by 2D NMR), 2.40 (s, 3H), 2.35 (s, 3H), 2.33 (s, 3H); ^{13}C NMR (126 MHz, DMSO-d_6, mm/nn = 90:10 isomeric peaks): 173.9 (C=O), 143.9 (C), 142.20 (C), 142.15 (C), 141.7 (C), 140.8 (C), 139.9 (C), 138.0 (C), 136.7 (C), 133.3 (C), 131.3 (C), 130.5 (C), 130.3 (C), 128.4 (C), 127.90/127.85 (C), 126.5/126.4 (C), 113.0 (C), 109.9 (C), 42.8/42.4 (CH), 41.0 (CH), 32.7/31.5 (2CH_2), 13.9 (CH_3), 12.8 (CH_3), 12.6 (CH_3), 12.0 (CH_3); HRMS (ESI+) calcd. for $C_{26}H_{19}NO_4S_8$ $[M+H]^+$: 665.9153, found: 665.9164.

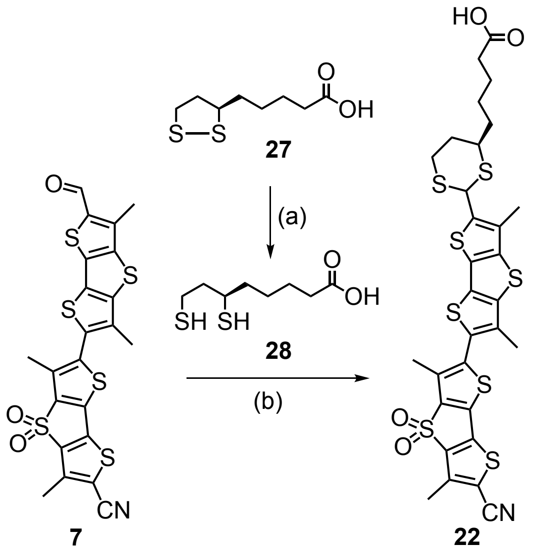

Scheme S3. (a) $NaHCO_3$, $NaBH_4$, 0 °C, 40%; (b) $BF_3·Et_2O$, anhydrous CH_2Cl_2, N_2, rt, 12 h, 60%.

Compound 28 was prepared according to the procedures reported in ref. S7.

Compound 22. To a stirred solution of $BF_3·Et_2O$ (5.2 μL, 0.042 mmol) and **7** (15.0 mg, 0.028 mmol) in anhydrous CH_2Cl_2 (8 mL), a solution of **28** (29.1 mg, 0.141 mmol) in anhydrous CH_2Cl_2 (2 mL) was added dropwise under N_2 atmosphere. The reaction mixture was stirred at room temperature for 12 h and the solvent was removed under reduced pressure. The reaction residue was suspended in diethyl ether (3×5 mL) and the orange precipitate was collected by centrifugation to afford **22** (12 mg, 60%, mixture of 4:96 diastereomers). IR (neat): 2921 (br, w, COOH), 2212 (m, C≡N), 1704 (s, C=O), 1506 (w), 1409 (m), 1313 (m, SO_2), 1142 (s, SO_2), 1090 (w), 1048 (w), 933 (w), 805 (w), 763 (w), 663 (w); ^{1}H NMR (500 MHz, CD_2Cl_2, mm/nn = 4:96 isomeric peaks): 5.76/5.64 (s, 1H), 3.13 – 2.98 (m, 3H), 2.58 (s, 3H), 2.41 (s, 3H), 2.39 (s, 3H), 2.36 (t, $^3J_{H\text{-}H}$ = 7.5 Hz), 2.35 (s, 3H), 2.23 – 2.18 (m, 1H), 1.68 – 1.53 (m, 7H); ^{13}C NMR (126 MHz, CD_2Cl_2, mm/nn = 4:96 isomeric peaks): 176.5 (C=O), 145.5 (C), 143.9 (C), 143.03 (C), 142.95 (C), 141.6 (C), 140.7 (C), 138.4 (C), 137.3 (C), 133.1 (C), 132.8 (C), 132.5 (C), 131.5 (C), 128.9 (C), 128.8 (C), 126.4 (C), 112.9 (C), 109.5 (C), 46.7/42.1 (CH_2), 46.0/38.9 (CH), 36.1 (CH), 33.6 (CH_2), 33.1 (CH_2), 32.7 (CH_2), 26.0 (CH_2), 24.9 (CH_2), 14.2

(CH_3), 13.4 (CH_3), 13.3 (CH_3), 12.5 (CH_3); HRMS (ESI+) calcd. for $C_{30}H_{27}NO_4S_8$ $[M+H]^+$: 721.9779, found: 721.9723.

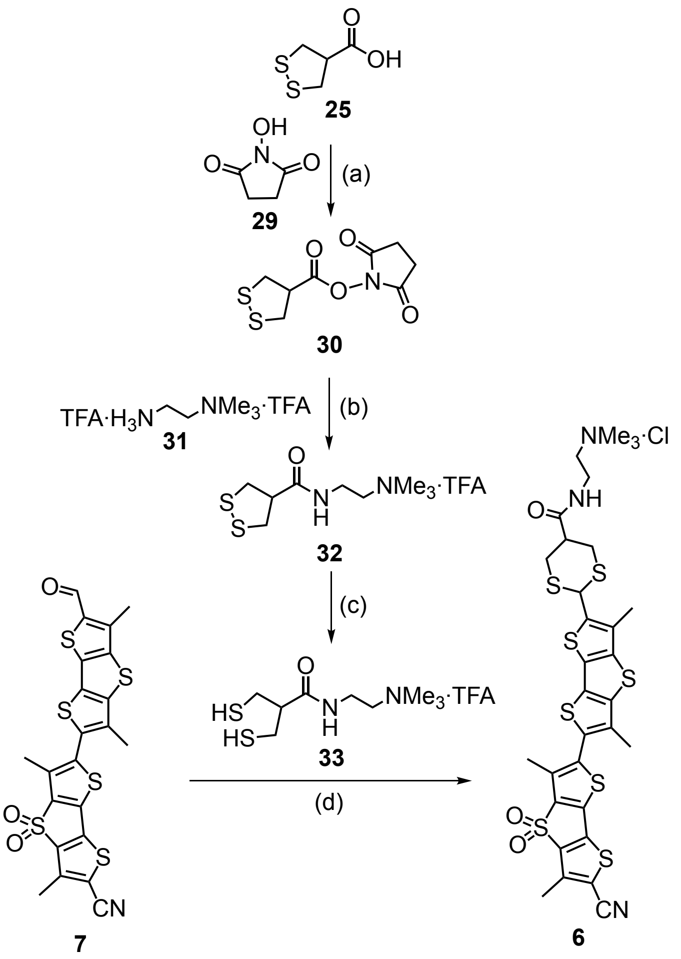

Scheme S4. (a) EDC·HCl, dry CH_2Cl_2, 0 °C to rt, 30 min, 42%; (b) DIPEA, dry DMF, rt, 2 h; (c) THPP, dry DMF, rt, 30 min; (d) HCl (4 M) in dioxane, MS (3 Å), dry DMF, rt, 24 h, 63%.

Compound 30 was prepared from **25** according to the procedures reported in ref. S8.

Compound 31 was prepared according to the procedures reported in ref. S9.

Compound 32. To a solution of **31** (60.0 mg, 0.184 mmol) and DIPEA (100 µL, 0.46 mmol) in dry DMF (4.0 mL), a solution of **30** (50.0 mg, 0.202 mmol) in dry DMF (1.0 mL) was added dropwise under N_2 atmosphere. The reaction mixture was stirred at room temperature for 2 h. The solvent was removed under reduced pressure. The residue was triturated with diethyl ether (3×5 mL), and CH_2Cl_2 (3×5 mL), and dried under vacuum to afford **32** as a colorless solid, which was used for

the next step without further purification. ^{1}H NMR (400 MHz, D_2O) 3.73 (t, $^3J_{H-H}$ = 6.8 Hz, 2H), 3.51 (t, $^3J_{H-H}$ = 6.7 Hz, 2H), 3.48 – 3.31 (m, 5H), 3.20 (s, 9H); ^{13}C NMR (101 MHz, D_2O) 175.7 (C=O), 64.8 (CH_2), 54.0 ($3CH_3$), 51.6 (CH), 42.6 ($2CH_2$), 34.4 (CH_2); MS (ESI): 235 ([M - TFA]$^+$).

Compound 6. To a solution of THPP (93.3 mg, 0.448 mmol) in dry DMF (2.5 mL), a solution of **32** (121 mg, 0.348 mmol) in dry DMF (2.5 mL) was added dropwise under N_2 atmosphere. The reaction mixture was stirred at room temperature for 30 min and the resulting solution containing **33** was added to **7** (45.0 mg, 0.085 mmol), followed by dry DMF (4.0 mL), HCl (4 M in dioxane, 1.5 mL), and molecular sieves (oven-dried, 3 Å, 45.0 mg). The reaction mixture was stirred at room temperature for 24 h. Molecular sieves were filtered off, and the filtrate was concentrated *in vacuo*. The residue was purified by column chromatography (basic Al_2O_3, CH_2Cl_2 for eluting **7**, then MeOH for eluting the mixture containing **6**), **7** (8 mg) was recovered. The fractions containing **6** were concentrated, and then the residue was triturated with CH_2Cl_2 (3×10 mL), and the orange precipitate was collected by centrifugation to afford **6** (40 mg, 63%, mixture of 70:30 diastereomers) as an orange solid. IR (neat): 3367 (br, w, N-H), 2208 (m, C≡N), 1650 (br, m, C=O), 1550 (w), 1478 (w), 1417 (m), 1305 (s, SO_2), 1200 (w), 1144 (s, SO_2), 1089 (w), 956 (w), 758 (w); ^{1}H NMR (500 MHz, DMSO-d_6, mm/nn = 70:30 isomeric peaks, some isomeric peaks overlapped with solvent peak): 8.53/8.32 (t, $^3J_{H-H}$ = 5.0 Hz, 1H), 6.10/5.94 (s, 1H), 3.61 – 3.30 (6H, overlapped with H_2O peak, assigned by 2D NMR), 3.17/3.10 (s, 9H), 2.96 (dd, $^2J_{H-H}$ = 14.1,$^3J_{H-H}$ = 2.6 Hz, 2H), 2.78 – 2.72 (m, 1H), 2.48 (s, 3H), 2.40/2.37 (s, 3H), 2.34 (s, 3H), 2.32 (s, 3H); ^{13}C NMR (126 MHz, DMSO-d_6, mm/nn = 70:30 isomeric peaks): 173.5/171.3 (C=O), 144.0 (C), 142.33 (C), 142.27/142.1 (C), 141.8/142.0 (C), 140.8 (C), 139.9 (C), 136.8/139.0 (C), 138.1 (C), 133.4 (C), 131.48/131.54 (C), 130.7 (C), 130.3/130.2 (C), 128.6/127.7 (C), 128.0/127.8 (C), 126.56/126.35 (C), 113.1 (C), 110.0 (C), 64.0/61.5 (CH_2), 53.0/52.7 ($3CH_3$), 42.9/41.4 (CH), 41.8 (CH), 33.2/34.0 (CH_2), 33.5/30.1 ($2CH_2$), 13.9 (CH_3), 12.8/12.9 (CH_3), 12.7 (CH_3), 12.1 (CH_3); HRMS (ESI+) calcd for $C_{31}H_{32}N_3O_3S_8^+$ [M]$^+$: 750.0204,

found: 750.0174.

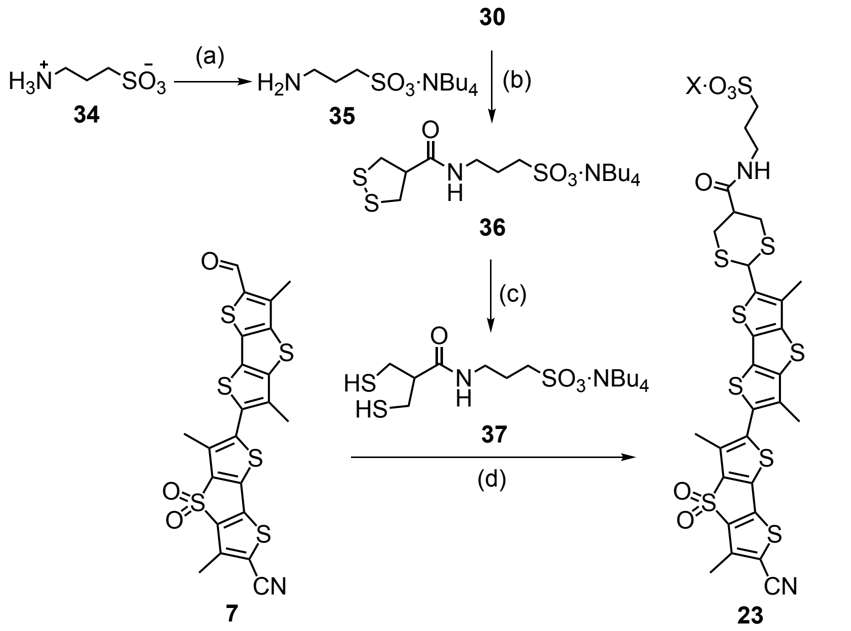

Scheme S5. (a) Bu_4NOH, MeOH, rt; (b) dry CH_2Cl_2/dry DMF 5:1, rt, 30 min; (c) THPP, dry DMF, rt, 30 min; (d) HCl (4 M) in dioxane, MS (3 Å), dry CH_2Cl_2/dry DMF 3:1, rt, 24 h, 8%.

Compound 35 was prepared according to the procedures reported in ref. S9.

Compound 36. To a solution of **35** (70.0 mg, 0.184 mmol) in dry CH_2Cl_2 (3.0 mL), a solution of **30** (50.0 mg, 0.202 mmol) in dry CH_2Cl_2/dry DMF (2:1, 3.0 mL) was added dropwise under N_2 atmosphere. The reaction mixture was stirred at room temperature for 30 min. The solvent was removed under reduced pressure. The reaction residue was triturated with diethyl ether (3×5 mL), then with pentane (3×5 mL) and dried under vacuum to afford **36** (+ 0.6 eq TBA) as a yellow oil, which was used for the next step without further purification. ^1H NMR (400 MHz, D_2O) 3.47 – 3.29 (m, 7H), 2.97 – 2.90 (m, 2H), 2.01 – 1.92 (m, 3H); ^{13}C NMR (101 MHz, D_2O) 174.5 (C=O), 51.4 (CH), 48.5 (CH_2), 42.2 ($2CH_2$), 38.2 (CH_2), 24.0 (CH_2). MS (ESI): 270 ($[M - Bu_4N]^-$).

Compound 23. To a solution of THPP (129 mg, 0.619 mmol) in dry DMF (5.0 mL), a solution of **36** (222 mg, 0.433 mmol) in dry CH_2Cl_2 (5.0 mL) was added. The reaction mixture was stirred at room temperature for 30 min and the resulting solution containing **37** was added to a solution

of **7** (35.0 mg, 0.066 mmol) in dry CH_2Cl_2 (10 mL). To the mixture were added dry DMF (3.0 mL), HCl (4 M in dioxane, 3.0 mL), and molecular sieves (oven-dried, 3 Å, 60.0 mg). The reaction mixture was stirred at room temperature for 24 h. The molecular sieves were filtered off, and the filtrate was concentrated under reduced pressure. The residue was purified by column chromatography (SiO_2, CH_2Cl_2/MeOH 50:1 to 10:1), and then, by reverse phase flash column chromatography (C18 20 g, CH_3CN + 0.1% TFA / H_2O + 0.1% TFA 1:9 to 4:6) to afford **23** (4.0 mg, 8%, mixture of 40:60 diastereomers) as an orange solid. R_f (**23** with X = TBA, CH_2Cl_2/MeOH 10:1, 0.40). IR (neat): 3294 (br, w, N-H), 2922 (br, w), 2212 (m, C≡N), 1634 (m, C=O), 1548 (w), 1409 (w), 1310 (m, SO_2), 1141 (s, SO_2), 1041 (m), 903 (w), 557 (s); ^{1}H NMR (500 MHz, DMSO-d_6, mm/nn = 40:60 isomeric peaks, some isomeric peaks overlapped with solvent peak): 8.15/7.97 (t, $^3J_{H-H}$ = 5.5 Hz, 1H), 6.06/5.81 (s, 1H), 3.29 – 3.27 (m, 1H, overlapped with H_2O peak, assigned by 2D NMR), 3.19 – 3.08 (m, 4H), 2.92 (dd, $^2J_{H-H}$ = 14.2, $^3J_{H-H}$ = 2.6 Hz, 1H), 2.78/2.68 (m, 1H), 2.48 (s, 3H, overlapped with DMSO peak, assigned by 2D NMR), 2.46 – 2.42 (m, 2H), 2.40/2.36 (s, 3H), 2.35/2.34 (s, 3H), 2.33 (s, 3H), 1.78 – 1.69 (m, 2H); ^{13}C NMR (126 MHz, DMSO-d_6, mm/nn = 40:60 isomeric peaks): 172.8/170.8 (C=O), 144.0 (C), 142.3 (C), 142.20/142.15 (C), 141.8/141.7 (C), 140.87/140.90 (C), 137.03/140.29 (C), 139.9 (C), 138.1/138.2 (C), 133.4/133.3 (C), 131.5/131.4 (C), 130.64/130.58 (C), 130.4/130.5 (C), 128.5/127.3 (C), 128.0/127.5 (C), 126.5/126.3 (C), 113.1 (C), 109.92/109.88 (C), 49.3/49.4 (CH_2), 42.9 (CH), 37.5/41.7 (CH), 38.2/38.6 (CH_2), 33.8/30.1 (2CH_2), 25.4/25.3 (CH_2), 13.9/13.6 (CH_3), 12.8/13.1 (CH_3), 12.7 (CH_3), 12.1 (CH_3); HRMS (ESI-) calcd for $C_{29}H_{25}N_2O_6S_9^-$ $[M – X]^-$: 784.9204, found: 784.9205.

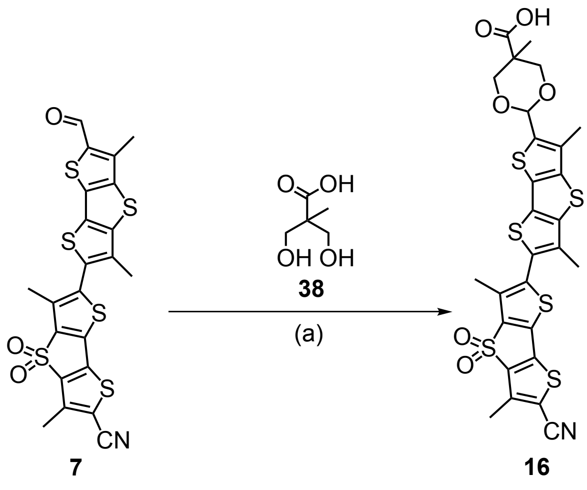

Scheme S6. (a) $TsOH{\cdot}H_2O$, trimethyl orthoformate, dry CH_2Cl_2, rt, 48 h, 16%.

Compound 16. To a stirred solution of $TsOH{\cdot}H_2O$ (26.8 mg, 0.141 mmol) and **7** (50.0 mg, 0.094 mmol) in anhydrous CH_2Cl_2 (43 mL) at rt under N_2 atmosphere, a solution of **38** (126 mg, 0.940 mmol) and trimethyl orthoformate (310 μL, 2.82 mmol) in anhydrous CH_2Cl_2 (3 mL) was added. The reaction mixture was stirred at room temperature for 48 h. The mixture was extracted with CH_2Cl_2/brine, and the organic phase was washed with saturated $NaHCO_3$ (3×50 mL), dried with Na_2SO_4, and concentrated under reduced pressure. The residue was purified by PTLC (CH_2Cl_2, R_f = 0) and triturated with diethyl ether (3×5 mL) to afford **16** (10 mg, 16%, mixture of 90:10 diastereomers) as an orange solid together with unreacted **7** (36 mg). R_f (CH_2Cl_2/MeOH/CH_3COOH 20:1:0.001): 0.28; IR (neat): 2853 (br, w, COO-H), 2214 (m, C≡N), 1706 (s, C=O), 1413 (m), 1370 (m), 1313 (s, SO_2), 1139 (s, SO_2), 1090 (s), 1027 (m), 946 (w), 660 (w); ^{1}H NMR (500 MHz, DMSO-d_6, mm/nn = 90:10 isomeric peaks): 5.94/5.92 (s, 1H), 4.41/4.13 (d, $^2J_{H\text{-}H}$ = 11.0 Hz, 2H), 3.76/3.90 (d, $^2J_{H\text{-}H}$ = 11.0 Hz, 2H), 2.48 (s, 3H), 2.34 (s, 6H), 2.33 (s, 3H), 0.95 (s, 3H); ^{13}C NMR (126 MHz, DMSO-d_6, mm/nn = 90:10 isomeric peaks): 175.4 (C=O), 143.9 (C), 142.3 (C), 142.2 (C), 141.9 (C), 140.8 (C), 139.9 (C), 138.0 (C), 136.6 (C), 133.3 (C), 131.5 (C), 130.6 (C), 130.2 (C), 128.5 (C), 128.4 (C), 126.3 (C), 113.1 (C), 109.9 (C), 96.2 (CH), 72.7 ($2CH_2$), 41.5 (C), 17.6/19.1 (CH_3), 13.9 (CH_3), 12.8 (CH_3), 12.7 (CH_3), 12.1 (CH_3); HRMS (ESI+) calcd for $C_{27}H_{21}NO_6S_6$ $[M+H]^+$: 647.9766,

found: 647.9756.

3. Theoretical Methods

The geometry optimization and energy calculations have been performed using the PBE0 functional[S10] adding Grimme's D3 dispersion correction[S11] and combined with the def2-TZVP basis set.[S12] The type I geometries have been fully optimized at the PBE0-D3/ def2-TZVP level of theory without symmetry constrains and the minimum nature of the complexes confirmed by frequency calculations. The type-II geometries were optimized imposing the planarity between the electron poor and electron rich dithienothiophene rings. The program used for the geometric and energetic calculations was Turbomole 7.2.[S13] The MEP surfaces were computed using the 0.001 a.u. isovalue. The natural bond orbital (NBO) analysis,[S14] that is convenient to analyze charge transfer effects, has been performed at the same level using the NBO7.0 program.[S15] The NCI (Non-Covalent Interaction) plot[S16] is a visualization index based on the density and its derivatives. It enables identification of non-covalent interactions. It is based on the peaks that appear in the reduced density gradient (RDG) at low densities.[S17] The interactions revealed by NCI correspond to both favorable and unfavorable interactions. In order to differentiate between them, the sign of the second density Hessian eigenvalue times the density is implemented. This value is able to characterize the strength of the interaction by means of the density, and its curvature thanks to the sign of the second eigenvalue. The following settings have been used in this work: RDG isosurface 0.6 a.u., density cut-off 0.04 a.u., color range –0.04 a.u. $\leq (\mathrm{sign}\lambda_2)\rho \leq$ 0.04 a.u. The NBO and NCIplot representations were performed using the VMD software.[S18]

3.1. NBO Analysis

The existence and estimated strength of the chalcogen bond were confirmed by using the NBO analysis and the concomitant stabilization energy of the systems due to the lone pair LP(O,S)→σ*(S–C) electron transfer. A very small orbital contribution was found in compounds **10–14** (Figure S2) and a negligible contribution <0.05 kcal/mol was found in compounds **7a** and **9**, which suggest that in the latter the ChB is insignificant from an orbital point of view. The contributions are larger in compounds **11**, **12** and **14** than in **10** and **13**. The most important contribution is observed in compound **12** followed by **14**. The plots of the orbitals involved in the ChB interaction are given in Figure S2, showing that the LP located at the O/S-atoms points to the antibonding σ*(S–C) orbital.

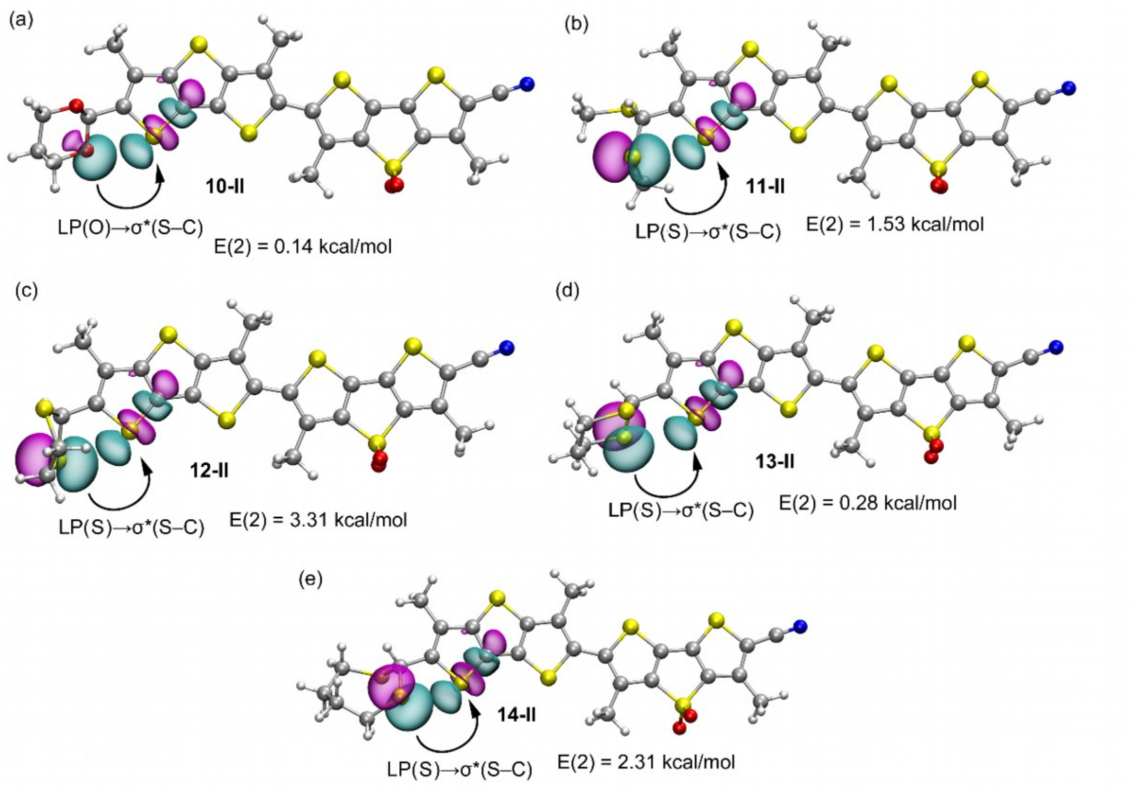

Figure S2. NBOs involved in the ChB interactions in compounds **10-II** (a), **11-II** (b), **12-II** (c), **13-II** (d) and **14-II** (e). The stabilization energies due to the orbital interactions are indicated.

3.2. Bifurcated Nature of The Chalcogen Bonds in Compound 13-II

As commented in the main text and indicated in Figure S2, the small NBO energy and longer S···S distance in compound **13-II** compared to **12-II** and **14-II** compounds is likely due to the bifurcated nature of the interaction. In Figure S3, the optimized geometry is shown where it can be observed that both S···S distances are similar (3.46 and 3.39 Å) and shorter than $\Sigma R_{vdw}(S+S) = 3.6$ Å.

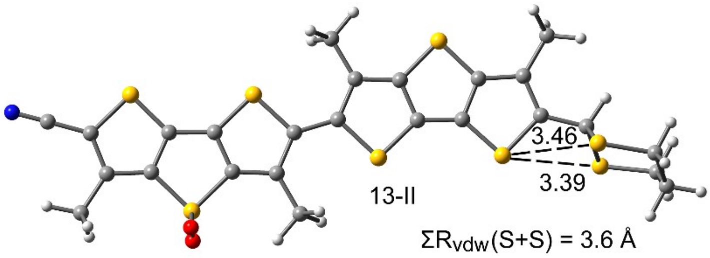

Figure S3. Geometries of the chalcogen bonding minimum obtained for compound **13-II**. Distances in Å.

3.3. NCIplot Analysis

In order to confirm the existence, attractive and bifurcated nature of the chalcogen bonding in **13**, noncovalent interaction plots (NCIPlot) was used. This method is very convenient to reveal noncovalent interactions and represent them in real space. The NCIplot of compound **13-II** along with those of **12-II** and **14-II** for comparison purposes are represented in Figure S4. Interestingly, in case of compounds **12-II** and **14-II** only one RDG isosurface (disk shape green isosurface) is observed between one S-atom of the 5 or 7 membered cyclic thioacetals and the S-atom of the dithienothiophene ring, evidencing the existence of a single S···S chalcogen bonding contact. The dark-green colour of the RDG isosurfaces agrees with the existence of a moderate (attractive) interaction. In contrast, for compound **13-II** the NCIplot analysis reveals the existence of two RDG isosurfaces of different sizes, the largest one corresponds to the shortest distance (Figure S3). This

analysis confirms the bifurcated nature of the interaction in **13-II** and its different binding mode with respect to **12-II** and **14-II**.

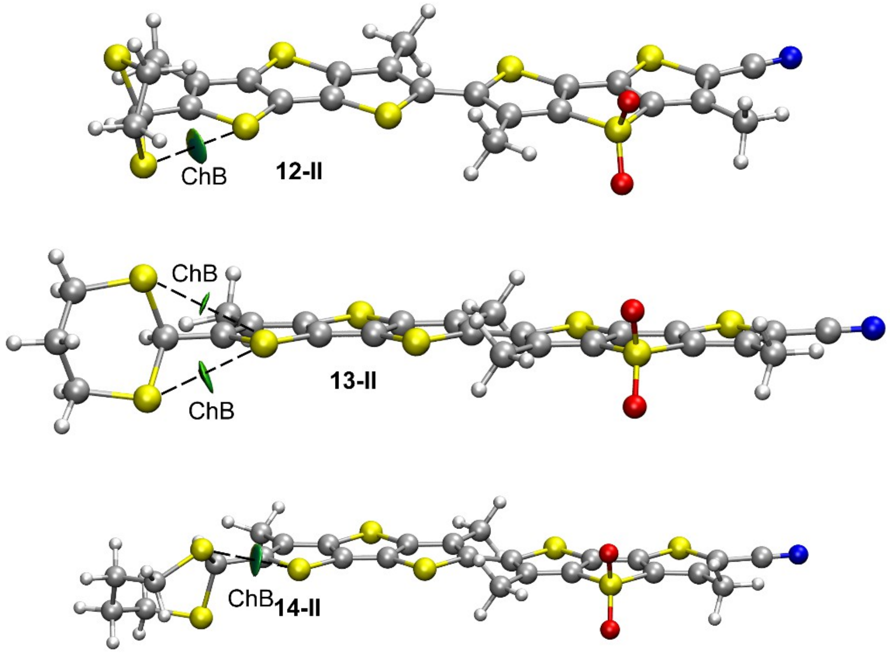

Figure S4. NCIplot of compounds **12-II**, **13-II** and **14-II**. Isosurface 0.6 a.u.

3.4. Rotational Profiles

The dependence of E_{rel} for angle β was analyzed by considering the planar (α = 180°) and rotated conformations (α = 90°) of the arenes (Scheme S7). As shown in Figure S5, the energy profiles varying β for both α rotamers separately and overlapped in the same representation. In these plots, we have also included both S···O distances. It can be observed the existence of three minima, which are very close in energy (within 0.2 kcal/mol). The three minima are represented in Figure S6, where it can be observed that one minimum exhibit two H-bonds (120°) and the other two minima present one HB and one ChB. In fact, the energy minimum at 250° approximately coincides with the minimum of one of the S···O distances (orange line) and the minimum at 350° with the minimum of the other S···O distance (blue line). The barrier that connects the latter two minima is smaller compared to the barriers that connect the H-bonded minimum with the other two. In any case the energetic barriers are small (< 2.5 kcal/mol), so all minima are accessible at room temperature. The overlapped plot demonstrates negligible differences between both profiles (α = 90° and 180°). Only the minima at 250°and 350° are slightly disfavored with α = 90° with respect to those with α = 180°

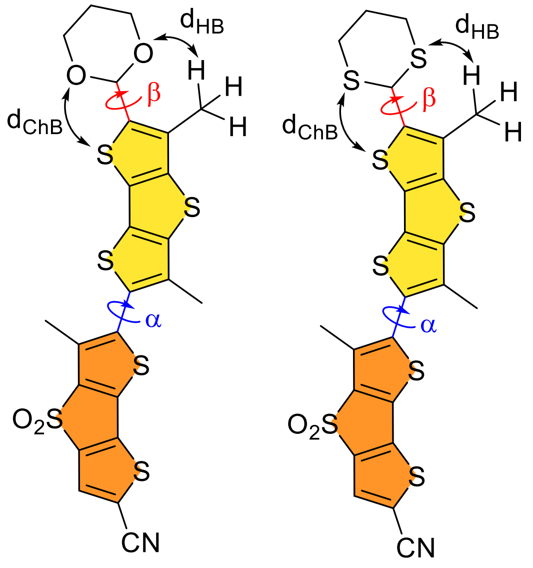

10 **13**

Scheme S7. Compounds **10** and **13** studied in this section.

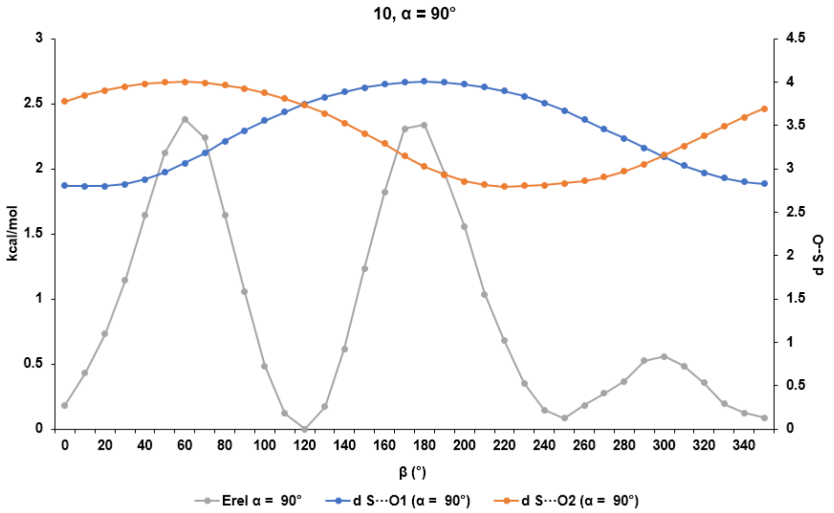

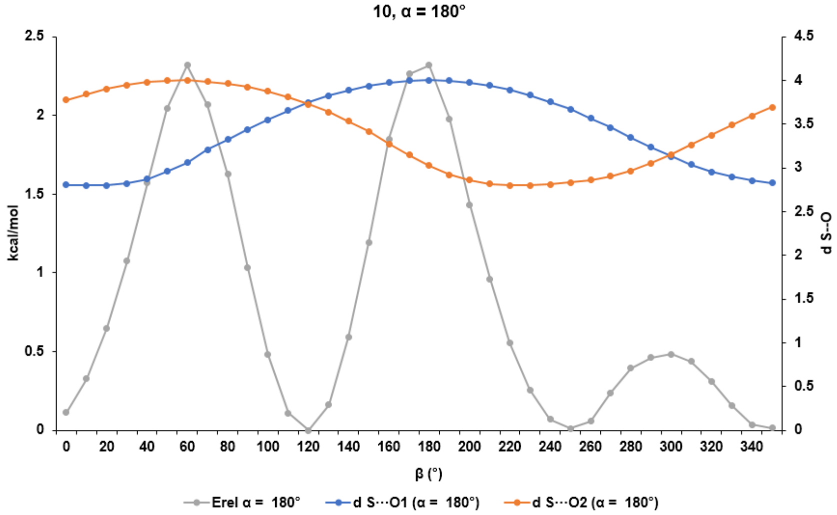

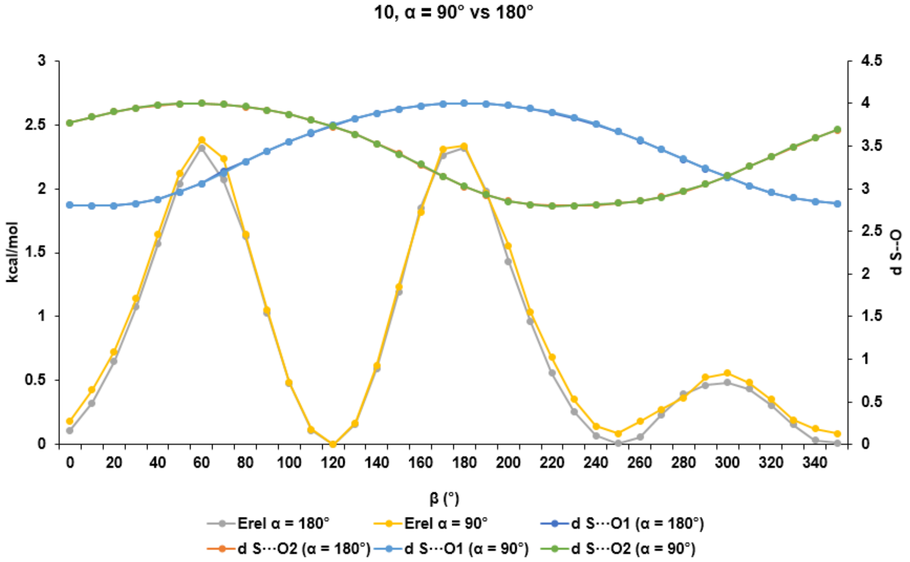

Figure S5. Energy profiles for β angle using α = 90°(top), α = 90°(middle) and both plots overlapped (bottom) for compound **10**.

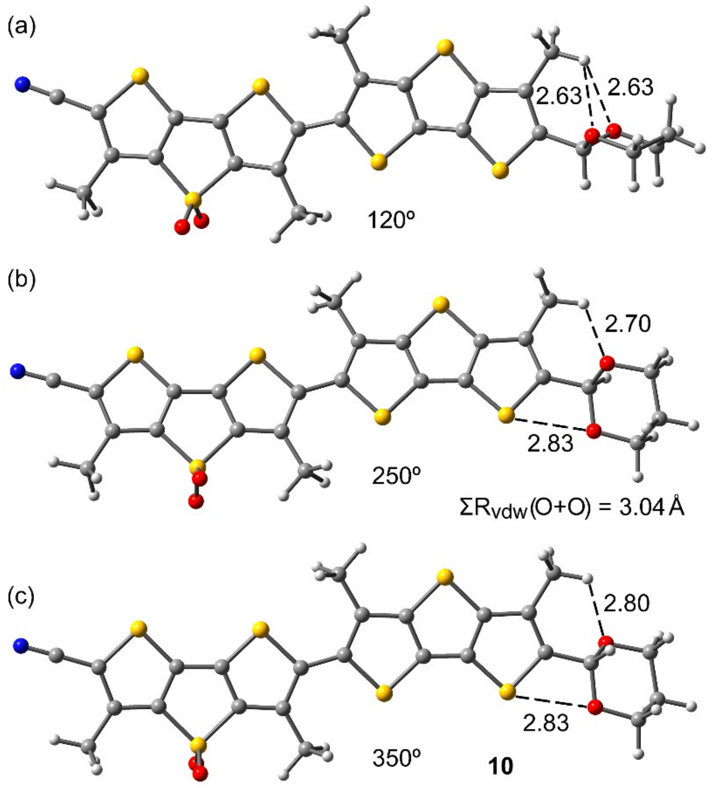

Figure S6. Geometries of the three minima (α = 180°) obtained for compound **10**. Distances in Å.

The same analysis was performed for compound **13** (Figure S7), showing some interesting differences with respect to **10**. Firstly, only two minima are found, one at 60°and the other one at 150° with a larger barrier connecting them (~6 kcal/mol). In the minimum at 60 degrees two ChBs are formed (Figure S8a) and the global minimum at 150° (Figure S8b) corresponds to the geometry with two CH···S H-bonds. The plot where both energy profiles are overlapped (Figure S7, bottom) shows that the α angle does not influence the β rotation, since both profiles are identical.

With respect to the S···S distances, they are also identical in both profiles. This behavior is also observed in compound **10** for the O···S ChB distances. Therefore, a shortening of the ChBs in the α = 180° rotamer with respect to the α = 90° is not observed. A likely explanation is that the ChB distances are already very short, 0.21 Å shorter than the ΣR_{vdW} for both the S···O and S···S contacts (Figure S6 and S8), due to the intramolecular nature of the interaction and that the donor and acceptor atoms are only separated by three covalent bonds.

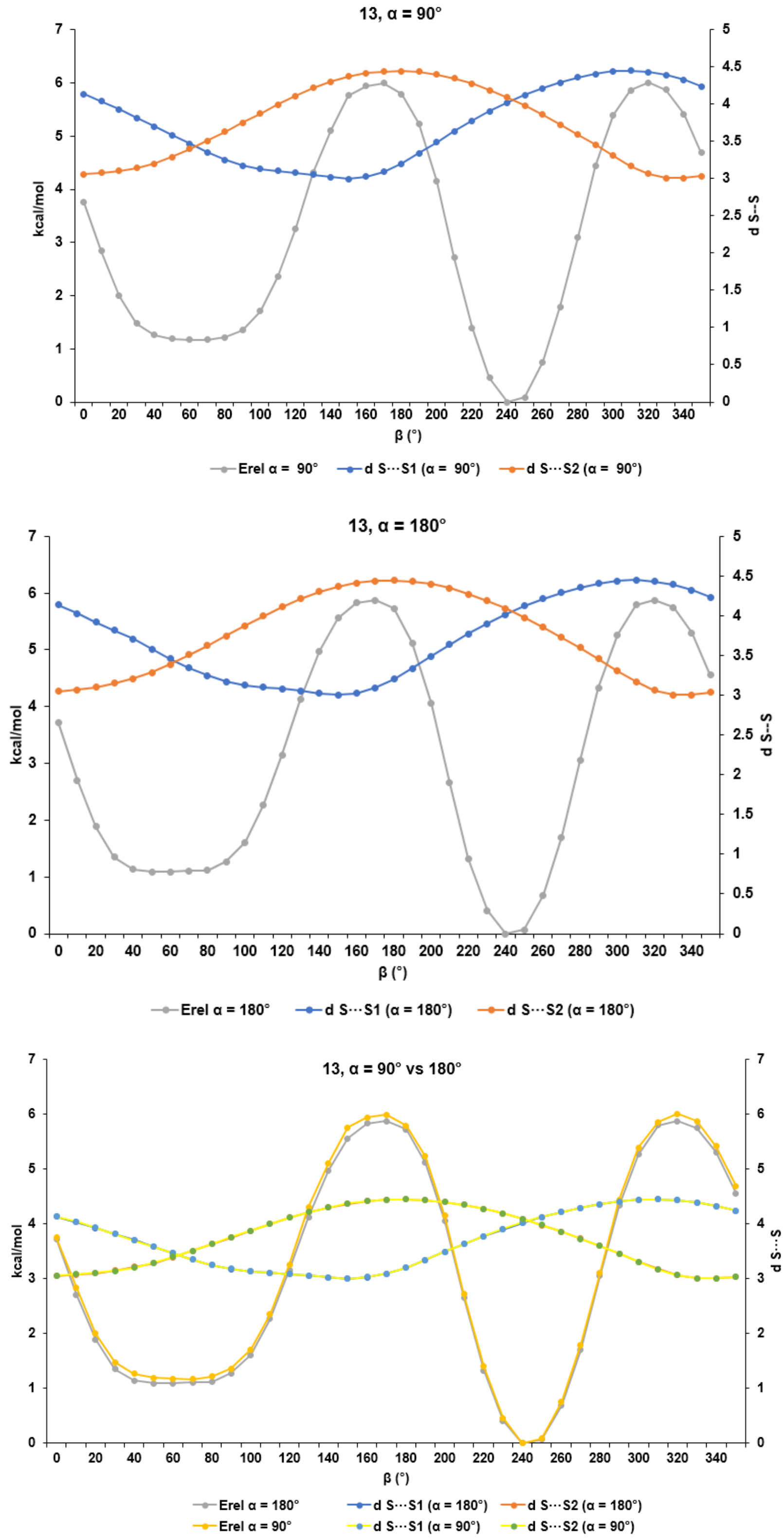

Figure S7. Energy profiles for β angle using α = 90°(top), α = 90° (top left) and both plots overlapped (bottom) for compound **2**.

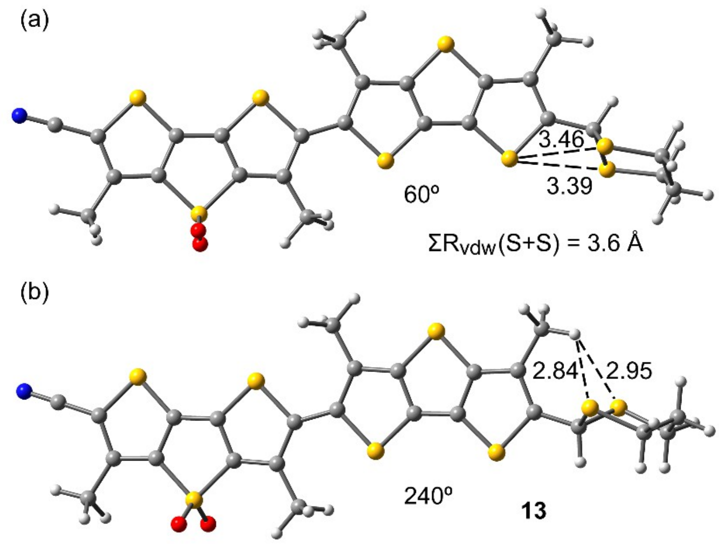

Figure S8. Geometries of the two minima (α = 180°) obtained for compound **13**. Distances in Å.

3.5. Cartesian Coordinates

7a-I

S	-0.6159754	2.1025545	4.4844455
S	0.3981343	-1.0532049	1.9635688
S	0.7778873	-1.6041477	5.8023761
C	-0.0988720	0.7937210	5.4975087
C	0.4872476	-0.6127170	7.1992442
C	0.2737319	-0.3366795	4.7762637
C	0.1372954	-0.1379395	3.3843271
C	0.0181633	0.6510999	6.8949363
C	-0.3323142	1.1325902	3.0794095
C	-0.4861982	1.3829176	1.6939556
C	0.7558892	-1.1798454	8.5050344
H	0.5546071	-0.5049856	9.3600078
C	-0.3268157	1.7362107	7.8564233
H	-1.3741076	2.0280978	7.7373065
H	-0.1727477	1.4450101	8.8930800
H	0.2808238	2.6237976	7.6586619
C	-0.9822057	2.6631663	1.1185629
H	-1.0192022	2.6196018	0.0303582
H	-1.9869285	2.8884295	1.4872412
H	-0.3350646	3.4954351	1.4090743
C	-0.1639915	0.1210939	-0.4789985
S	1.2282996	0.5131006	-1.4368416
C	-1.2304205	-0.2976169	-1.2464645
C	0.4101061	0.1249629	-2.8723638
C	-0.8724261	-0.2839861	-2.6081253
C	0.7305740	0.1265444	-4.2800329
S	-1.7371001	-0.6711737	-4.1024578
S	2.1030276	0.5103119	-5.1991077
C	-0.3113584	-0.2768668	-5.0789598
C	1.2624611	0.1232758	-6.6650999
C	-0.0404388	-0.2916008	-6.4560087
C	-0.1207411	0.2749805	0.9693198
C	-2.5642591	-0.6999461	-0.7215662
H	-2.7983267	-1.7246958	-1.0192052
H	-2.5915694	-0.6353483	0.3656729
H	-3.3457510	-0.0563948	-1.1331143
C	-1.0041334	-0.6891319	-7.5161529
H	-1.3627814	-1.7055545	-7.3368035
H	-1.8758564	-0.0303944	-7.4984511
H	-0.5491329	-0.6444147	-8.5048423
C	1.9122027	0.2458655	-7.9083311
N	2.4396703	0.3451253	-8.9296993

O	-2.0303466	-2.0729088	-4.1771660
O	-2.7865420	0.2781350	-4.3372754
O	1.1699374	-2.3018753	8.6872875

7a-II

S	-2.3476167	0.0065412	4.3979784
S	1.0789943	0.0134757	2.0450471
S	1.5455990	-0.0049595	5.9063229
C	-0.9904961	0.0004545	5.4785924
C	0.4475226	-0.0144378	7.2528280
C	0.2333716	0.0039942	4.8139546
C	0.0647997	0.0104540	3.4139032
C	-0.8848859	-0.0105178	6.8828475
C	-1.2705209	0.0119322	3.0388912
C	-1.4966084	0.0148318	1.6481908
C	1.0076834	-0.0245182	8.5884159
H	0.2637342	-0.0329082	9.4089273
C	-2.0672480	-0.0120081	7.7904196
H	-2.7229434	-0.8547447	7.5552837
H	-1.7913641	-0.0809208	8.8404593
H	-2.6517294	0.9019507	7.6520772
C	-2.8741778	0.0163384	1.0741869
H	-3.0613586	-0.8667423	0.4574591
H	-3.6155456	0.0178261	1.8743730
H	-3.0589971	0.8992855	0.4565944
C	-0.0645551	0.0137735	-0.4711715
S	-1.4243120	0.0150723	-1.5690083
C	1.1435174	0.0103695	-1.1749457
C	-0.4173924	0.0098995	-2.9304542
C	0.9007390	0.0083540	-2.5535507
C	-0.6113830	0.0043192	-4.3554138
S	1.9602702	0.0033530	-3.9780211
S	-1.9586960	0.0016773	-5.3873223
C	0.5647399	-0.0007255	-5.0685297
C	-0.9258594	-0.0066582	-6.7814077
C	0.4209793	-0.0071730	-6.4638343
C	-0.2916455	0.0147926	0.9540570
C	2.5264955	0.0081830	-0.6126383
H	3.2608593	0.0047237	-1.4177637
H	2.7118846	0.8920603	0.0021555
H	2.7075411	-0.8742477	0.0055125
C	1.5404510	-0.0137859	-7.4422209
H	2.1727417	0.8652373	-7.2963237
H	2.1689311	-0.8942657	-7.2887898
H	1.1726812	-0.0174085	-8.4675136
C	-1.4812398	-0.0129947	-8.0746657
N	-1.9318676	-0.0185295	-9.1370811
O	2.6785583	1.2405296	-4.0802905
O	2.6803071	-1.2335420	-4.0708740
O	2.1935569	-0.0238657	8.8289307

7b-I

S	-2.0528870	0.4247287	4.4887544
S	1.1709898	-0.1544600	1.9153190
S	1.8815640	-0.0826522	5.7499800
C	-0.6408279	0.2638592	5.4817527
C	0.8921964	0.1228998	7.1686041
C	0.5206480	0.0643783	4.7389675
C	0.2624595	0.0418678	3.3502958
C	-0.4477366	0.2999195	6.8767475
C	-1.0840206	0.2252269	3.0674701
C	-1.3987284	0.2085815	1.6863778
C	1.5679190	0.0912610	8.4559724
H	2.6661422	-0.0643079	8.3874965
C	-1.5506748	0.4976568	7.8570667
H	-2.2622434	-0.3312739	7.7908285
H	-1.1729609	0.5560961	8.8736629
H	-2.1015071	1.4133047	7.6225498
C	-2.7711687	0.3712464	1.1333767
H	-3.1862068	1.3461363	1.4042277
H	-2.7730076	0.2868532	0.0469004
H	-3.4423663	-0.3914277	1.5381165
C	-0.1609389	-0.0905544	-0.5061578
S	-0.0048151	1.3471782	-1.4644309
C	-0.1775414	-1.2358983	-1.2736575
C	0.0329639	0.4438715	-2.9011476

C	-0.0675954	-0.8983580	-2.6360257
C	0.1360463	0.7412493	-4.3100787
S	-0.0472809	-1.8455644	-4.1297154
S	0.2842702	2.1577170	-5.2307796
C	0.1100305	-0.3760143	-5.1089567
C	0.3094485	1.2335150	-6.6973544
C	0.2070126	-0.1300091	-6.4873867
C	-0.2611329	0.0086892	0.9436815
C	-0.3008091	-2.6238610	-0.7494943
H	-0.3993348	-2.6248764	0.3354935
H	-1.1722679	-3.1191056	-1.1844226
H	0.5771099	-3.2144517	-1.0223351
C	0.2008538	-1.1715281	-7.5481199
H	1.0368497	-1.8601719	-7.4036832
H	-0.7176714	-1.7605870	-7.4918030
H	0.2761124	-0.7292919	-8.5406471
C	0.4257834	1.8810888	-7.9422948
N	0.5213066	2.4053372	-8.9656683
O	1.1424808	-2.6416378	-4.2133646
O	-1.3219104	-2.4648779	-4.3522974
O	1.0351468	0.2176364	9.5338894

7b-II

S	-2.2272674	-0.0079473	4.4111736
S	1.1603054	-0.0062369	2.0006039
S	1.6769935	-0.0011004	5.8590748
C	-0.8541273	-0.0052540	5.4708236
C	0.6020879	-0.0012877	7.2296586
C	0.3581402	-0.0044917	4.7828951
C	0.1688242	-0.0054053	3.3853951
C	-0.7348211	-0.0029981	6.8732025
C	-1.1723195	-0.0072527	3.0338378
C	-1.4211709	-0.0099949	1.6467402
C	1.2120700	0.0024885	8.5494930
H	2.3232723	0.0104354	8.5355360
C	-1.8993892	-0.0063576	7.8012498
H	-2.4720747	-0.9308162	7.6792474
H	-1.5817374	0.0745403	8.8367975
H	-2.5714468	0.8229482	7.5625724
C	-2.8078496	-0.0132877	1.0951468
H	-3.0044515	0.8669312	0.4773057
H	-3.0029177	-0.8991318	0.4848094
H	-3.5362361	-0.0104560	1.9070835
C	-0.0239925	-0.0125392	-0.4961898
S	-1.4005661	-0.0161651	-1.5726911
C	1.1729715	-0.0123411	-1.2185029
C	-0.4147509	-0.0166767	-2.9497617
C	0.9090495	-0.0147867	-2.5930745
C	-0.6301663	-0.0149441	-4.3717444
S	1.9468939	-0.0121644	-4.0332212
S	-1.9925651	-0.0120888	-5.3837095
C	0.5352630	-0.0116831	-5.1023992
C	-0.9804541	-0.0056557	-6.7928690
C	0.3709038	-0.0059677	-6.4953459
C	-0.2282624	-0.0098585	0.9323992
C	2.5646486	-0.0088596	-0.6780040
H	2.7585136	-0.8891916	-0.0607421
H	3.2861495	-0.0117656	-1.4946608
H	2.7567147	0.8772809	-0.0684730
C	1.4760863	-0.0001302	-7.4898207
H	2.1096062	0.8778251	-7.3427659
H	2.1075426	-0.8816606	-7.3556370
H	1.0936839	0.0076647	-8.5096717
C	-1.5544262	0.0016501	-8.0779537
N	-2.0196738	0.0086906	-9.1340219
O	2.6635589	1.2257166	-4.1394516
O	2.6657054	-1.2484247	-4.1433297
O	0.6144225	-0.0018472	9.6007173

9-I

S	-1.6865249	1.7159841	4.1365685
S	0.6061241	-0.7756977	1.7177544
S	1.0555406	-1.0545442	5.5980155
C	-0.7154945	0.7459907	5.1933967
C	0.3488864	-0.2435143	6.9582863
C	0.0998872	-0.1410203	4.5106501

C	-0.0520901	-0.0292874	3.1091351
C	-0.5783645	0.7001224	6.6058306
C	-0.9894071	0.9321648	2.7588130
C	-1.1896048	1.0760505	1.3640811
C	0.7498162	-0.6766385	8.3239902
H	0.2040789	-1.5923259	8.6089311
O	2.1315926	-0.9225568	8.3517576
C	-1.3678059	1.5676087	7.5269032
H	-2.4215151	1.2731220	7.5344286
H	-1.0018957	1.5216658	8.5519085
H	-1.3252548	2.6101424	7.1996996
C	-2.1457000	2.0355439	0.7465407
H	-2.1340963	1.9627524	-0.3407048
H	-3.1649481	1.8416647	1.0927154
H	-1.8984839	3.0634560	1.0265882
C	-0.3031101	0.0381763	-0.7700800
S	0.8336988	0.9719155	-1.6908887
C	-1.0560583	-0.8016433	-1.5638117
C	0.3178934	0.2618605	-3.1434972
C	-0.6807403	-0.6510376	-2.9126333
C	0.6667818	0.3859436	-4.5385436
S	-1.2216450	-1.3964626	-4.4235085
S	1.7736245	1.3200321	-5.4211590
C	-0.0658857	-0.4341107	-5.3620398
C	1.2405107	0.5987157	-6.9052852
C	0.2373440	-0.3373866	-6.7288675
C	-0.3826350	0.2058265	0.6742558
C	-2.1140153	-1.7263244	-1.0721066
H	-1.8940770	-2.7534568	-1.3725691
H	-2.1905959	-1.6857486	0.0138599
H	-3.0827589	-1.4624412	-1.5037802
C	-0.4197561	-1.1177025	-7.8103739
H	-0.2816946	-2.1881363	-7.6391906
H	-1.4955644	-0.9262376	-7.8116239
H	-0.0146533	-0.8618991	-8.7885621
C	1.8198724	0.9846225	-8.1291202
N	2.2904391	1.2990595	-9.1347251
O	-0.8699126	-2.7865358	-4.4602971
O	-2.5677265	-1.0081751	-4.7330688
C	2.5589324	-1.4402763	9.5834910
H	3.6320269	-1.6132451	9.5085450
H	2.0587989	-2.3905293	9.8172791
H	0.4754018	0.0990111	9.0535373
H	2.3677369	-0.7356924	10.4046231

9-II

S	-2.5392992	-0.0170155	4.0379001
S	0.9427529	-0.1878040	1.7804986
S	1.2852489	-0.2251361	5.6719956
C	-1.2173881	-0.1055968	5.1548112
C	0.1529040	-0.1597283	6.9835777
C	0.0150789	-0.1750064	4.5254087
C	-0.1090949	-0.1456307	3.1194634
C	-1.1520194	-0.0925783	6.5719351
C	-1.4296017	-0.0642976	2.7050445
C	-1.6137082	-0.0337255	1.3083569
C	0.6783176	-0.2421203	8.3728777
H	0.9196360	-1.2881303	8.6273982
O	1.8314020	0.5506834	8.4868181
C	-2.3629463	-0.0282673	7.4403926
H	-2.9214277	-0.9682592	7.4028053
H	-2.1138887	0.1697062	8.4823689
H	-3.0357655	0.7631728	7.0989313
C	-2.9716390	0.0520907	0.6953746
H	-3.1852256	-0.8067368	0.0533313
H	-3.7354739	0.0753860	1.4738500
H	-3.0917067	0.9559635	0.0920790
C	-0.1238924	-0.0775624	-0.7699810
S	-1.4485172	0.0188410	-1.9067388
C	1.1025189	-0.1309207	-1.4403000
C	-0.4031233	-0.0059276	-3.2403091
C	0.9006431	-0.0895181	-2.8245926
C	-0.5514582	0.0527944	-4.6686447
S	2.0013148	-0.1095806	-4.2165169
S	-1.8629316	0.1652266	-5.7404713

C	0.6449443	0.0138834	-5.3469864
C	-0.7875223	0.1600229	-7.1032074
C	0.5461772	0.0735247	-6.7443659
C	-0.3924387	-0.0908754	0.6468976
C	2.4663314	-0.2158406	-0.8383449
H	3.2235554	-0.2377748	-1.6218625
H	2.6765069	0.6435902	-0.1974595
H	2.5846014	-1.1193273	-0.2357746
C	1.6958400	0.0503794	-7.6871176
H	2.3666205	0.8881285	-7.4823543
H	2.2737669	-0.8672692	-7.5537951
H	1.3626483	0.1101044	-8.7225353
C	-1.2993595	0.2407777	-8.4113563
N	-1.7138554	0.3069550	-9.4865890
O	2.8017395	1.0803957	-4.2473892
O	2.6444598	-1.3859348	-4.3410260
C	2.4375001	0.4354115	9.7470078
H	3.3233332	1.0695756	9.7406800
H	2.7399144	-0.6001087	9.9561979
H	1.7651861	0.7699190	10.5491756
H	-0.0953768	0.0881828	9.0809561

10-I

S	-2.1746724	1.4613328	3.3519056
S	0.6161423	-0.5924905	1.0466833
S	0.9322708	-0.8150737	4.9399858
C	-1.0963699	0.6724390	4.4549028
C	0.0226046	-0.1622641	6.2570817
C	-0.1030660	-0.0511820	3.8126746
C	-0.2184420	0.0280711	2.4048581
C	-1.0348888	0.6137871	5.8697029
C	-1.2919316	0.8132665	2.0100083
C	-1.4520535	0.9260001	0.6072828
C	0.4390794	-0.4549723	7.6583709
H	-0.4143128	-0.8624488	8.2373854
O	1.4549655	-1.4118361	7.6307550
O	0.8500958	0.7385419	8.2709123
C	-1.9857581	1.3178463	6.7757877
H	-3.0199421	1.1067162	6.4896109
H	-1.8451068	1.0274182	7.8157099
H	-1.8427182	2.4006240	6.7160076
C	-2.5346075	1.7040970	-0.0553156
H	-2.4545268	1.6505178	-1.1408847
H	-3.5174278	1.3226710	0.2359477
H	-2.4942393	2.7556043	0.2424711
C	-0.3230468	0.0467704	-1.4818042
S	0.6818735	1.1404490	-2.3791779
C	-0.9235344	-0.8958251	-2.2901162
C	0.3175811	0.3574918	-3.8401476
C	-0.5404535	-0.6929250	-3.6297346
C	0.6764590	0.5323774	-5.2271671
S	-0.9470489	-1.4959351	-5.1530856
S	1.6662140	1.6097710	-6.0852627
C	0.0858000	-0.3814154	-6.0655994
C	1.2772644	0.8200195	-7.5793419
C	0.4091199	-0.2458899	-7.4243320
C	-0.4775634	0.2106373	-0.0431577
C	-1.8460740	-1.9658437	-1.8205632
H	-1.4574671	-2.9509200	-2.0893746
H	-1.9734231	-1.9212927	-0.7395344
H	-2.8245186	-1.8624095	-2.2961598
C	-0.1028228	-1.1131080	-8.5181030
H	0.1783732	-2.1532535	-8.3359132
H	-1.1944020	-1.0738824	-8.5498454
H	0.2895477	-0.8060335	-9.4867433
C	1.8292747	1.2800717	-8.7900426
N	2.2774846	1.6531667	-9.7857457
O	-0.4144239	-2.8270978	-5.1944083
O	-2.3277791	-1.2850456	-5.4818390
C	1.8839267	-1.7581597	8.9348287
H	2.6918877	-2.4798308	8.8114201
H	1.0589224	-2.2543575	9.4694954
C	1.2240471	0.5142407	9.6189616
H	0.3470695	0.1742556	10.1938311
H	1.5374395	1.4796503	10.0173521

C	2.3299932	-0.5212420	9.6908342
H	2.5565560	-0.7713399	10.7310817
H	3.2345462	-0.1180052	9.2288697

10-II

S	-2.6982202	-0.2661034	3.2659252
S	0.8409550	-0.0639146	1.1006801
S	1.0941146	-0.1816571	4.9929536
C	-1.4021687	-0.2539951	4.4166933
C	-0.0704258	-0.2803569	6.2659428
C	-0.1515353	-0.1829255	3.8201340
C	-0.2434645	-0.1435565	2.4114939
C	-1.3675537	-0.3121896	5.8312351
C	-1.5547704	-0.1807737	1.9633745
C	-1.7053418	-0.1452966	0.5628222
C	0.3874665	-0.2938924	7.6845079
H	-0.0678305	-1.1460663	8.2286054
O	1.7748674	-0.4439267	7.7025622
O	-0.0277753	0.8927888	8.3068236
C	-2.5802500	-0.3770858	6.6951988
H	-3.2646213	-1.1537046	6.3427010
H	-2.3271428	-0.5824209	7.7343482
H	-3.1198324	0.5738931	6.6716659
C	-3.0493988	-0.1789894	-0.0850065
H	-3.1680744	-1.0496486	-0.7355264
H	-3.8319195	-0.2293324	0.6733948
H	-3.2353388	0.7139959	-0.6878575
C	-0.1650106	-0.0355901	-1.4759615
S	-1.4630492	-0.0519522	-2.6464557
C	1.0782873	0.0190544	-2.1143530
C	-0.3851098	0.0109531	-3.9527222
C	0.9097582	0.0437378	-3.5033774
C	-0.5003290	0.0308498	-5.3848394
S	2.0448342	0.0973668	-4.8669268
S	-1.7891006	0.0155721	-6.4894601
C	0.7125455	0.0755742	-6.0334034
C	-0.6826822	0.0698229	-7.8258780
C	0.6441112	0.0992137	-7.4337918
C	-0.4677058	-0.0807205	-0.0667756
C	2.4284118	0.0478369	-1.4773628
H	3.2047530	0.0893522	-2.2411521
H	2.5499054	0.9207951	-0.8320454
H	2.6055859	-0.8435050	-0.8711280
C	1.8146491	0.1477929	-8.3494716
H	2.4158108	1.0368819	-8.1449304
H	2.4560466	-0.7215692	-8.1865440
H	1.5026182	0.1651365	-9.3930563
C	-1.1665251	0.0815398	-9.1470574
N	-1.5585944	0.0922633	-10.2326611
O	2.7345016	1.3546856	-4.9107837
O	2.8053183	-1.1169719	-4.9398461
C	2.2808996	-0.4843889	9.0243697
H	3.3641933	-0.5699324	8.9365121
H	1.9050996	-1.3877989	9.5301077
C	0.3599571	0.9153094	9.6694537
H	-0.1517941	0.1041520	10.2123665
H	0.0122277	1.8664480	10.0735617
C	1.8636385	0.7572534	9.7889850
H	2.1601733	0.6758246	10.8383342
H	2.3556655	1.6332022	9.3587852

11-I

S	-0.7024551	2.5652186	3.0911405
S	0.3087584	-0.7675709	0.8123321
S	0.6779058	-1.0138108	4.7024393
C	-0.2068106	1.3305367	4.2010158
C	0.3657538	0.0670094	6.0206053
C	0.1614440	0.1548962	3.5667290
C	0.0441174	0.2508913	2.1607553
C	-0.0916100	1.2955459	5.6129931
C	-0.4138920	1.4972831	1.7584277
C	-0.5566568	1.6453806	0.3570500
C	0.5930943	-0.3730878	7.4353760
H	1.1761818	0.3985835	7.9507898
C	-0.4186933	2.4563746	6.4888070
H	-1.4900981	2.6739836	6.4592952

H	-0.1539262	2.2738772	7.5287760
H	0.1084257	3.3511179	6.1450800
C	-1.0382530	2.8845638	-0.3131160
H	-1.0699062	2.7632428	-1.3957377
H	-2.0425701	3.1447733	0.0328902
H	-0.3854311	3.7301981	-0.0793036
C	-0.2252629	0.2299554	-1.7175957
S	1.1788166	0.5480416	-2.6867696
C	-1.2841661	-0.2388471	-2.4668924
C	0.3750716	0.0633411	-4.1006028
C	-0.9121085	-0.3225313	-3.8223908
C	0.7097512	-0.0322587	-5.5012821
S	-1.7611900	-0.8118899	-5.2956641
S	2.0933515	0.2851638	-6.4292087
C	-0.3247087	-0.4863802	-6.2823846
C	1.2675073	-0.1984156	-7.8751922
C	-0.0387540	-0.5954007	-7.6520437
C	-0.1950308	0.4866606	-0.2844112
C	-2.6244500	-0.5979064	-1.9274471
H	-2.8578206	-1.6422259	-2.1477295
H	-2.6610604	-0.4495269	-0.8486639
H	-3.4003471	0.0134795	-2.3947836
C	-0.9913575	-1.0640166	-8.6928577
H	-1.3315145	-2.0765809	-8.4624110
H	-1.8752387	-0.4219612	-8.7107068
H	-0.5336301	-1.0621396	-9.6813154
C	1.9305193	-0.1608706	-9.1167357
N	2.4690272	-0.1310474	-10.1368698
O	-2.0536366	-2.2159873	-5.2785776
O	-2.8095926	0.1163672	-5.6083208
C	0.5243413	-3.2205938	7.3510147
H	0.2247622	-3.3071457	6.3074003
H	1.0830801	-4.1121441	7.6386544
H	-0.3609916	-3.1281887	7.9805729
C	-0.4817471	-0.6489975	9.9754568
H	0.0782794	0.2274986	10.3069903
H	-1.3845227	-0.7425782	10.5798476
H	0.1323224	-1.5422011	10.0974964
S	1.6473200	-1.8450379	7.6367911
S	-1.0199911	-0.4678018	8.2712904
**11-II**			
S	0.3880383	2.7311566	3.0279376
S	0.0592799	-0.7973974	0.8603370
S	0.1285189	-1.0454118	4.7573563
C	0.2950287	1.4387355	4.1789413
C	0.2252150	0.1126354	6.0417381
C	0.1837953	0.1930268	3.5802674
C	0.1737995	0.2831701	2.1716855
C	0.3208566	1.4077531	5.5941785
C	0.2756324	1.5913471	1.7243514
C	0.2636600	1.7424950	0.3234005
C	0.1836082	-0.3281867	7.4727087
H	0.9740249	0.1980552	8.0194909
C	0.4404463	2.6292055	6.4398114
H	-0.4575205	3.2473480	6.3544056
H	0.5685214	2.3881649	7.4937388
H	1.2908589	3.2355730	6.1148853
C	0.3651803	3.0830977	-0.3244223
H	1.2444629	3.1599138	-0.9695172
H	-0.5143630	3.3107346	-0.9328022
H	0.4482471	3.8628179	0.4338826
C	0.0896461	0.2089340	-1.7160315
S	0.1768675	1.5040562	-2.8866227
C	-0.0397066	-1.0289709	-2.3537905
C	0.0438908	0.4319290	-4.1923305
C	-0.0629984	-0.8587320	-3.7426429
C	0.0182402	0.5486712	-5.6245223
S	-0.1989867	-1.9876196	-5.1052700
S	0.0938051	1.8347409	-6.7297132
C	-0.1060146	-0.6591101	-6.2720308
C	-0.0459814	0.7341840	-8.0646290
C	-0.1481204	-0.5886479	-7.6718815
C	0.1475674	0.5089672	-0.3066506
C	-0.1448586	-2.3753411	-1.7168017

H	-0.2306555	-3.1480109	-2.4805624
H	0.7355110	-2.6031712	-1.1113029
H	-1.0231263	-2.4471074	-1.0711565
C	-0.2825447	-1.7538806	-8.5857160
H	0.5580648	-2.4384448	-8.4490517
H	-1.1940847	-2.3101041	-8.3543168
H	-0.3152962	-1.4400741	-9.6283460
C	-0.0505312	1.2190455	-9.3855360
N	-0.0563523	1.6126336	-10.4705949
O	0.9648192	-2.8223492	-5.1869655
O	-1.4978660	-2.5962174	-5.1390025
C	-0.8929838	-2.9701503	7.3927281
H	-1.0256273	-3.1265655	6.3229486
H	-0.7985720	-3.9363298	7.8892076
H	-1.7560299	-2.4392589	7.7981057
C	-1.0979035	-0.0980062	9.9241485
H	-0.2785009	0.5197749	10.2964041
H	-2.0130527	0.1695159	10.4533586
H	-0.8711874	-1.1504614	10.0980184
S	0.6163526	-2.0705279	7.7804854
S	-1.4037370	0.2108240	8.1814034

12-I

S	-0.8392656	2.6817936	3.0805597
S	0.3253093	-0.6385078	0.8582696
S	0.6709170	-0.8233803	4.7515038
C	-0.2960416	1.4865851	4.2108648
C	0.3133881	0.2701468	6.0476571
C	0.1215781	0.3166540	3.5971062
C	0.0105125	0.3884332	2.1895210
C	-0.1898822	1.4732467	5.6251751
C	-0.5013306	1.6066452	1.7655361
C	-0.6501096	1.7237951	0.3618342
C	-0.6088403	2.6102945	6.4936324
H	-1.6810656	2.8000566	6.3888909
H	-0.4112190	2.4197207	7.5479336
H	-0.0866511	3.5278691	6.2074643
C	-1.1839384	2.9295809	-0.3294455
H	-1.2149865	2.7864938	-1.4094011
H	-2.1964243	3.1562681	0.0164664
H	-0.5645121	3.8049523	-0.1149578
C	-0.2476862	0.2885916	-1.6882144
S	1.1416587	0.6627238	-2.6584757
C	-1.2687017	-0.2697106	-2.4285483
C	0.3786525	0.0906305	-4.0623997
C	-0.8827772	-0.3695345	-3.7789100
C	0.7257507	-0.0190602	-5.4591479
S	-1.6827060	-0.9709723	-5.2379148
S	2.0843715	0.3768978	-6.3939598
C	-0.2715096	-0.5644398	-6.2304136
C	1.2977897	-0.1962424	-7.8291600
C	0.0230739	-0.6811006	-7.5974676
C	-0.2385105	0.5708774	-0.2594505
C	-2.5871493	-0.6978329	-1.8854721
H	-2.7548340	-1.7596820	-2.0806861
H	-2.6386342	-0.5256867	-0.8108378
H	-3.3968105	-0.1470704	-2.3706275
C	-0.8940781	-1.2352343	-8.6280206
H	-1.1646166	-2.2638363	-8.3772483
H	-1.8196707	-0.6553581	-8.6579176
H	-0.4369960	-1.2217954	-9.6167015
C	1.9580114	-0.1375628	-9.0713489
N	2.4941442	-0.0911261	-10.0920989
O	-1.8599765	-2.3929025	-5.1728427
O	-2.8012254	-0.1431517	-5.5868802
C	-0.5751971	-2.1348119	9.0389193
C	0.4765541	-0.1702586	7.4635135
C	0.4672363	-2.8000173	8.1683749
H	-0.1612081	-1.8616621	10.0112663
H	-1.4459525	-2.7788264	9.1889728
H	0.8538055	0.6647956	8.0564232
H	0.0137567	-3.2422106	7.2799015
H	1.0201184	-3.5706121	8.7111692
S	1.6653782	-1.5319118	7.7046739
S	-1.1505845	-0.6785606	8.1460564

12-II

S	-2.8402208	0.3880536	2.9922397
S	0.7194578	0.0244563	0.8833141
S	0.9156545	0.1248225	4.7804148
C	-1.5653692	0.3042175	4.1630778
C	-0.2663306	0.2487225	6.0422579
C	-0.3111082	0.1783055	3.5863504
C	-0.3806183	0.1556368	2.1768317
C	-1.5544702	0.3451344	5.5793978
C	-1.6813416	0.2566703	1.7079621
C	-1.8109967	0.2305366	0.3052882
C	-2.7883609	0.4408314	6.4109723
H	-3.4146579	-0.4450598	6.2722620
H	-2.5646701	0.5207720	7.4743100
H	-3.3843629	1.3104299	6.1195911
C	-3.1412683	0.3290600	-0.3639438
H	-3.3601323	-0.5536687	-0.9707979
H	-3.9326809	0.4168627	0.3815702
H	-3.2074005	1.2047138	-1.0151673
C	-0.2466943	0.0405190	-1.7089025
S	-1.5239380	0.1209645	-2.8992619
C	1.0015382	-0.0832364	-2.3275462
C	-0.4308921	-0.0022057	-4.1885690
C	0.8532039	-0.1053177	-3.7189374
C	-0.5231498	0.0026172	-5.6226556
S	2.0057041	-0.2064775	-5.0651008
S	-1.7894071	0.1072989	-6.7483355
C	0.6968338	-0.0943200	-6.2518476
C	-0.6636814	0.0206293	-8.0666762
C	0.6523798	-0.0873325	-7.6532622
C	-0.5682715	0.1058844	-0.3046488
C	2.3388163	-0.1757845	-1.6696938
H	3.1233959	-0.2628996	-2.4211020
H	2.5519149	0.7109639	-1.0681868
H	2.4065229	-1.0480783	-1.0155949
C	1.8357146	-0.1781388	-8.5491453
H	2.5201413	0.6506096	-8.3531019
H	2.3839677	-1.1034563	-8.3561892
H	1.5432629	-0.1536364	-9.5982118
C	-1.1234865	0.0653128	-9.3957042
N	-1.4958759	0.1018301	-10.4876024
O	2.8344112	0.9635559	-5.1066554
O	2.6228851	-1.5007177	-5.1186870
C	1.3545132	-1.5983426	9.1481643
C	0.1526710	0.1524948	7.4698607
C	2.4856340	-0.9433517	8.3865410
H	1.1555442	-1.0778055	10.0870884
H	1.5670430	-2.6491909	9.3617445
H	-0.4682874	0.8204825	8.0694672
H	2.8102034	-1.5694139	7.5540001
H	3.3415511	-0.7317554	9.0321264
S	-0.0861301	-1.5613915	8.0680708
S	1.8848555	0.6493040	7.7801405

13-I

S	-2.7711958	0.7178901	2.8402711
S	0.6094484	-0.2473528	0.6134612
S	0.8883385	-0.3530188	4.5097678
C	-1.5134084	0.3464479	3.9721542
C	-0.2208243	-0.0534897	5.8150105
C	-0.3190547	0.0099629	3.3558779
C	-0.4201998	0.0476572	1.9463789
C	-1.4714660	0.3150236	5.3890132
C	-1.6890369	0.4155663	1.5223338
C	-1.8452231	0.4650022	0.1152592
C	0.2553075	-0.1803348	7.2162673
H	-0.6012789	-0.1040640	7.8902112
C	-2.6445872	0.6597639	6.2406537
H	-3.5213392	0.0810679	5.9370809
H	-2.4638748	0.4654098	7.2969220
H	-2.8988318	1.7184326	6.1345782
C	-3.1123428	0.8237808	-0.5790461
H	-2.9959128	0.7841772	-1.6618907
H	-3.9157038	0.1381540	-0.2948560
H	-3.4333625	1.8320018	-0.3028614

C	-0.4277012	0.0224495	-1.9385200
S	0.1611518	1.3931353	-2.8247724
C	-0.6249781	-1.0787146	-2.7454561
C	0.1507661	0.5200940	-4.2801030
C	-0.2869400	-0.7643097	-4.0754845
C	0.4745868	0.7997659	-5.6585434
S	-0.3147409	-1.6758790	-5.5912726
S	1.0451904	2.1528612	-6.5069838
C	0.2794585	-0.2715961	-6.4960224
C	1.0176623	1.2647388	-7.9959930
C	0.5817926	-0.0396644	-7.8467157
C	-0.6688431	0.1264260	-0.5062946
C	-1.1265126	-2.4024643	-2.2844007
H	-2.0539451	-2.6604810	-2.8019026
H	-0.4001631	-3.1866392	-2.5105372
H	-1.3126732	-2.3947227	-1.2110449
C	0.4505657	-1.0396996	-8.9389211
H	1.0628203	-1.9179807	-8.7204561
H	-0.5854761	-1.3783473	-9.0161208
H	0.7581050	-0.6246358	-9.8978820
C	1.4154796	1.8819283	-9.1973778
N	1.7405655	2.3826599	-10.1848641
O	0.6673303	-2.7214582	-5.5803850
O	-1.6630095	-1.9876103	-5.9699969
C	1.4474327	-1.6827366	9.2046638
H	1.9167677	-2.6401019	9.4411449
H	0.5302549	-1.6165994	9.8000526
C	1.7451422	0.8301684	9.3160508
H	0.8295483	0.9271357	9.9098393
H	2.4208932	1.6278681	9.6320230
C	2.3802852	-0.5311283	9.5347423
H	2.6625690	-0.6127290	10.5913513
H	3.2942462	-0.6124879	8.9408657
S	1.0025913	-1.8176244	7.4585499
S	1.3712352	1.2080566	7.5893595

13-II

S	-2.9234925	0.0114731	2.7340662
S	0.6903343	0.0119746	0.6863313
S	0.8064990	0.0133615	4.5842489
C	-1.6666844	0.0130976	3.9270355
C	-0.4040649	0.0161376	5.8325302
C	-0.3971340	0.0118398	3.3706441
C	-0.4397467	0.0111036	1.9599909
C	-1.6864070	0.0161311	5.3435929
C	-1.7361263	0.0101651	1.4688997
C	-1.8384759	0.0087155	0.0634065
C	0.0231275	0.0150520	7.2549030
H	-0.8644110	0.0295702	7.8912915
C	-2.9502266	0.0174586	6.1333435
H	-3.5405033	-0.8768933	5.9145277
H	-2.7705544	0.0449591	7.2069996
H	-3.5631293	0.8851192	5.8734475
C	-3.1601817	0.0069275	-0.6297996
H	-3.2892649	-0.8758334	-1.2617247
H	-3.9695165	0.0052480	0.1017516
H	-3.2920329	0.8898386	-1.2609897
C	-0.2260440	0.0094396	-1.9224942
S	-1.4825091	0.0069477	-3.1373645
C	1.0398049	0.0116732	-2.5170547
C	-0.3582994	0.0073814	-4.4052468
C	0.9206414	0.0104342	-3.9114760
C	-0.4233770	0.0027594	-5.8407644
S	2.1035256	0.0088189	-5.2351834
S	-1.6731113	-0.0050655	-6.9891701
C	0.8119065	0.0030327	-6.4467512
C	-0.5201210	-0.0082745	-8.2865162
C	0.7926335	-0.0032797	-7.8488998
C	-0.5785728	0.0094650	-0.5239037
C	2.3672016	0.0147247	-1.8333032
H	3.1706860	0.0160707	-2.5696923
H	2.4924487	0.8982131	-1.2031625
H	2.4963165	-0.8676056	-1.2023240
C	1.9950335	-0.0044984	-8.7236566
H	2.6054961	0.8796303	-8.5252388

H	2.6136722	-0.8797388	-8.5117320
H	1.7197956	-0.0137490	-9.7776565
C	-0.9579276	-0.0156582	-9.6237560
N	-1.3123771	-0.0218567	-10.7221974
O	2.8283671	1.2462689	-5.2723225
O	2.8325326	-1.2263059	-5.2648433
C	1.2705679	-1.2641200	9.3636710
H	1.8228012	-2.1557534	9.6685320
H	0.3247197	-1.2646803	9.9160975
C	1.3081340	1.2690245	9.3558183
H	0.3627821	1.3009202	9.9082220
H	1.8865611	2.1457653	9.6552255
C	2.0662334	-0.0080698	9.6707362
H	2.3065665	-0.0082352	10.7407535
H	3.0099677	-0.0237972	9.1197750
S	0.9711566	1.5276938	7.5994684
S	0.9267141	-1.5228285	7.6086518

14-I

S	-2.8412297	0.1635539	2.6048098
S	0.6633469	0.1328278	0.3602547
S	0.9825668	0.2502920	4.2561866
C	-1.5240399	0.1930359	3.7310151
C	-0.1599687	0.2722139	5.5634343
C	-0.2845788	0.1851376	3.1090662
C	-0.3999554	0.1606687	1.6997103
C	-1.4665137	0.2397022	5.1449261
C	-1.7230224	0.1467024	1.2822950
C	-1.8947325	0.1163184	-0.1231031
C	-2.6693126	0.2001566	6.0237284
H	-3.0125712	-0.8292117	6.1636524
H	-2.4699071	0.6168691	7.0116891
H	-3.4908457	0.7676107	5.5801466
C	-3.2161938	0.0860444	-0.8081087
H	-3.1007674	0.0646570	-1.8914403
H	-3.7869082	-0.7969356	-0.5062015
H	-3.8118918	0.9635271	-0.5411968
C	-0.4275848	0.0467420	-2.1862012
S	-0.2530589	1.5093146	-3.1037472
C	-0.3392616	-1.0808332	-2.9757250
C	-0.0561680	0.6401128	-4.5479615
C	-0.1290074	-0.7109696	-4.3180371
C	0.1650234	0.9721448	-5.9349168
S	0.0548284	-1.6225871	-5.8236193
S	0.3519574	2.4135735	-6.8088729
C	0.2513007	-0.1265579	-6.7550295
C	0.5479873	1.5249615	-8.2848545
C	0.4719178	0.1544460	-8.1122055
C	-0.6745846	0.1108219	-0.7524578
C	-0.4528151	-2.4823209	-2.4865786
H	0.4455803	-3.0497591	-2.7407134
H	-0.5898088	-2.5064504	-1.4059864
H	-1.2991691	-2.9850293	-2.9610975
C	0.6042293	-0.8623436	-9.1886447
H	1.4424778	-1.5295545	-8.9742673
H	-0.2965277	-1.4790733	-9.2367525
H	0.7643361	-0.3953783	-10.1595860
C	0.7617770	2.2044217	-9.4993013
N	0.9375017	2.7555566	-10.4977504
O	1.2722081	-2.3808924	-5.8213474
O	-1.1736768	-2.2762761	-6.1734604
C	0.3177105	0.2338895	6.9731525
H	-0.4607942	0.6580058	7.6120528
C	1.9512473	0.4845423	9.8547775
C	1.3625269	-0.8905500	10.1443173
C	0.1831748	-1.3115902	9.2858742
C	2.6948836	0.5843615	8.5231789
H	1.1757019	1.2560051	9.9088540
H	2.1536071	-1.6423684	10.0525992
H	-0.6588020	-0.6216001	9.4078030
H	3.1041656	-0.3863975	8.2349243
H	2.6608926	0.7130750	10.6559931
H	1.0318377	-0.9166672	11.1895467
H	-0.1703212	-2.2961220	9.6027383
H	3.5380904	1.2752142	8.6102312

S	0.4893062	-1.4947860	7.5120526
S	1.8069590	1.2798549	7.1182493

14-II

S	-2.6477688	-1.0125798	2.5133945
S	0.6562138	0.3881659	0.4205430
S	0.7804845	0.5264493	4.3175151
C	-1.4886872	-0.4878506	3.6913696
C	-0.3237927	0.0695998	5.5763273
C	-0.3239252	0.0039818	3.1196036
C	-0.3735796	-0.0354622	1.7093526
C	-1.5015959	-0.4565097	5.1050979
C	-1.5645020	-0.5629916	1.2342921
C	-1.6635634	-0.6378211	-0.1690290
C	-2.6200763	-0.9811123	5.9380397
H	-2.5278390	-2.0627962	6.0730485
H	-2.6390060	-0.5274426	6.9294049
H	-3.5829372	-0.7849465	5.4605755
C	-2.8784164	-1.1815062	-0.8444351
H	-3.3521855	-0.4395784	-1.4931583
H	-2.6485514	-2.0588214	-1.4553465
H	-3.6176713	-1.4850261	-0.1023097
C	-0.1956420	-0.0413053	-2.1763449
S	-1.3534145	-0.5742974	-3.3726886
C	0.9604171	0.4523656	-2.7895843
C	-0.3296482	-0.1566055	-4.6570126
C	0.8433456	0.3708796	-4.1817680
C	-0.3907323	-0.2311439	-6.0909074
S	1.9200648	0.8105654	-5.5223865
S	-1.5320857	-0.7792672	-7.2215554
C	0.7401105	0.2443389	-6.7140207
C	-0.4742118	-0.3645471	-8.5341169
C	0.7259985	0.1818325	-8.1146046
C	-0.5109625	-0.1488900	-0.7733045
C	2.1809686	0.9986504	-2.1248631
H	2.9150820	1.2982859	-2.8721732
H	1.9488897	1.8753196	-1.5154428
H	2.6516963	0.2556977	-1.4765959
C	1.8305399	0.6295051	-9.0036002
H	2.0432978	1.6881353	-8.8360467
H	2.7444205	0.0755038	-8.7753871
H	1.5815165	0.4813470	-10.0535913
C	-0.8700190	-0.5951297	-9.8644707
N	-1.1896427	-0.7828546	-10.9575971
O	2.0769163	2.2343630	-5.6034034
O	3.0890891	-0.0209347	-5.5308118
C	0.0824416	0.1759242	7.0047797
H	-0.8185090	0.2918358	7.6124447
C	1.4726326	0.9132608	9.9445677
C	1.3748715	-0.5854226	10.2009238
C	0.4248208	-1.3595762	9.3044241
C	2.1597782	1.2856300	8.6313241
H	0.4823880	1.3784189	9.9928198
H	2.3741289	-1.0265799	10.1224440
H	-0.6017560	-0.9911997	9.4067711
H	2.8776055	0.5169851	8.3378379
H	2.0519188	1.3492262	10.7646726
H	1.0491188	-0.7424306	11.2361860
H	0.4112819	-2.4113530	9.6015549
H	2.7176158	2.2193119	8.7461308
S	0.8207620	-1.3976512	7.5390236
S	1.1100574	1.6703171	7.2184201

4. Thioacetal Flipper Generations *in situ*

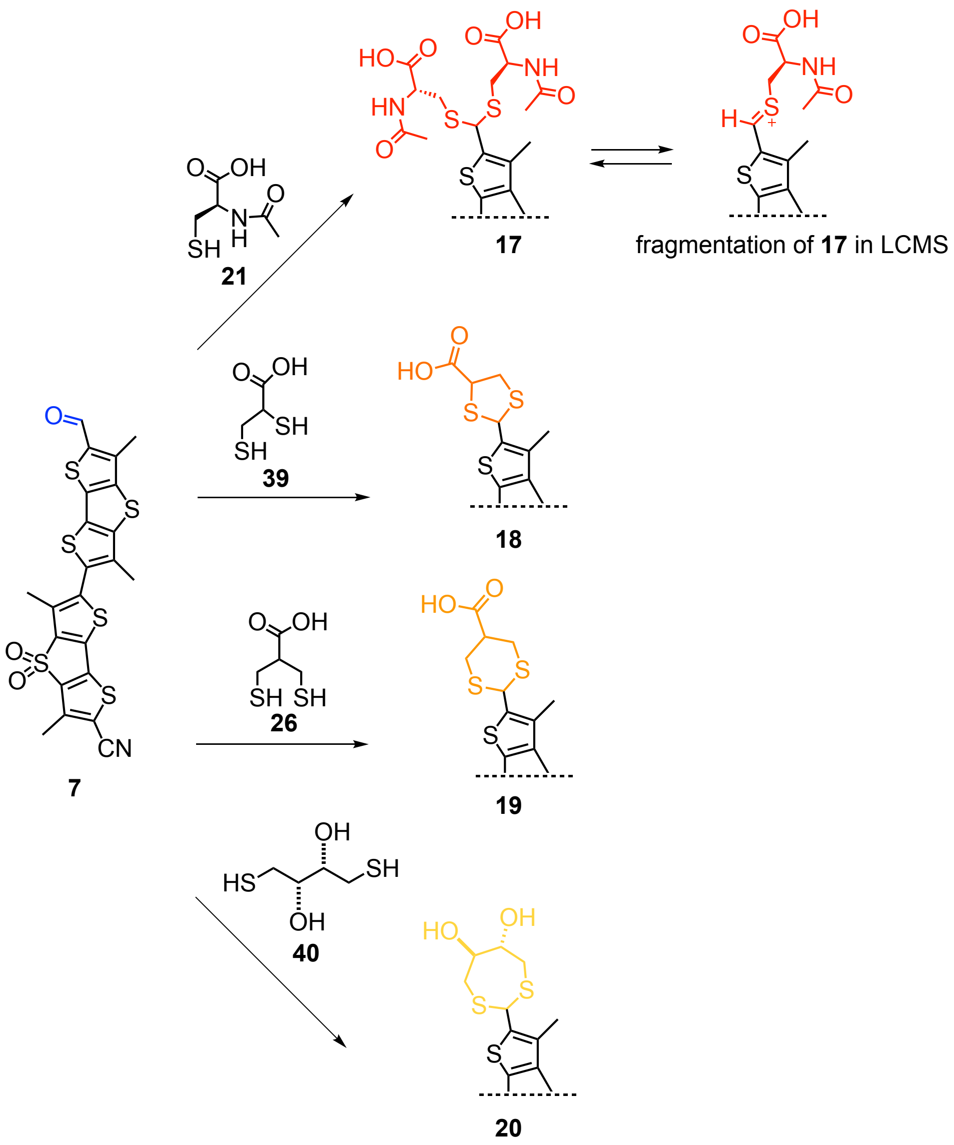

Scheme S8. Structure of the flipper probes formed *in situ*.

General Methods. To a solution of **7** (0.2 mM, 300 µL, 0.06 µmol) in CH_2Cl_2 were added HCl (4 M, 24 µL in dioxane, 96 µmol) and a solution of head group (*N*-acetyl-L-Cys **21**, 2,3-dimercaptopropanoic acid **39**, reduced asparagusic acid **26**, or DTT **40** in DMF; 6 µL of 1 M, 6 µmol) at 25 °C. Absorption spectra of the mixture (l = 0.1 cm) were measured every 1 ~ 5 min, as indicated, using the corresponding mixture without **7** as blank. LC-MS of the reaction mixture (Figure S11-S14) indicated the complete consumption of **7** and the exclusive formation of the desired product. For

FLIM imaging in GUVs and HK cells, the reaction was run in the way as described above with **7** (1.0 mM, 300 µL, 0.3 µmol) in CH_2Cl_2. Then, the reaction mixture was diluted with brine and CH_2Cl_2. The organic phase was dried over Na_2SO_4 and filtered through a cotton plug in a pipette. The filtrate was concentrated, and the residue was dissolved in DMSO (0.5 mL for **17**, **19**, **20**, and 1.0 mL for **18**) for the FLIM measurements in GUVs and HK cells. Assuming the quantitative product formation, the estimated concentration of the probe is 0.6 mM (**17, 19**, and **20**) or 0.3 mM (**18**).

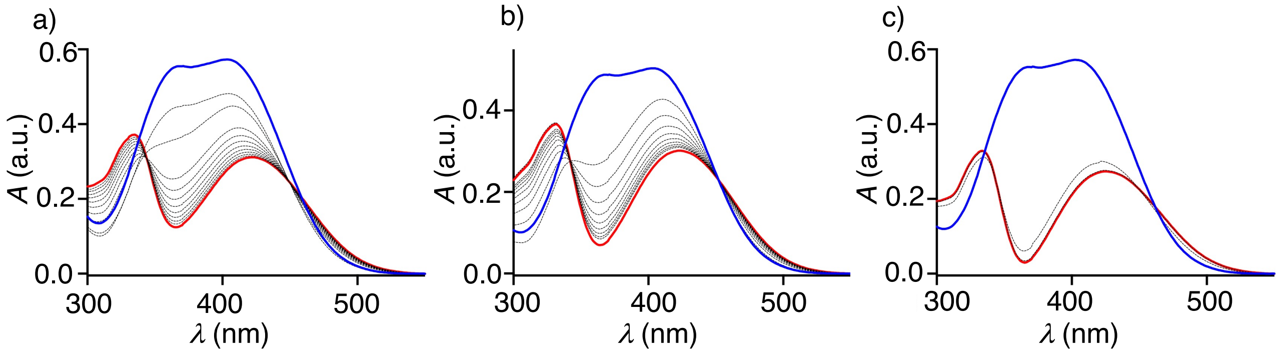

Figure S9. Absorption spectra of **7** in CH_2Cl_2 before (blue line) and after addition of a) 2,3-dimercaptopropanoic acid (**39**, 5-50 min: black dashed line, 60 min: red line), b) reduced asparagusic acid (**26**, 1-20 min: black dashed line, 30 min: red line) or c) DTT (**40**, 1-5 min: black dashed line, 10 min: red line) and 4 M HCl in dioxane.

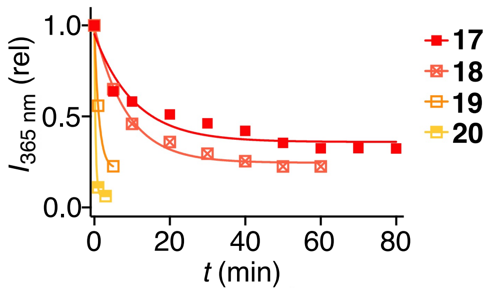

Figure S10. Kinetics of the conversion of **7** to **17** (red), **18** (light red), **19** (orange) and **20** (yellow) *in situ*. Data points were fit to the exponential decay function.

*Reaction of 7 with **41** in situ.* Compound **41** was prepared according to the procedures reported in ref. S19. A solution of **41** (1 M, 30 μL) in DMF was mixed with THPP (1 M, 60 μL) in DMF and kept for 10 min (Scheme S9). The resulting solution of **42** was added to a solution of **7** (1.0 mM, 300 μL) in CH_2Cl_2 along with HCl (4 M, 120 μL in dioxane) at 25 °C. After 48 h, the same workup was done as the general methods described, and the reaction mixture was dissolved in DMSO (0.5 mL) for FLIM imaging in GUVs and HK cells. The formation of **24** *in situ* was proved based on LCMS (Figure S15), which showed the presence of residual **7** (Figure S16). Assuming the quantitative formation of **24**, the concentration was estimated to be 0.5 mM. The presence of **7** was found not to affect the FLIM imaging (Figure S27).

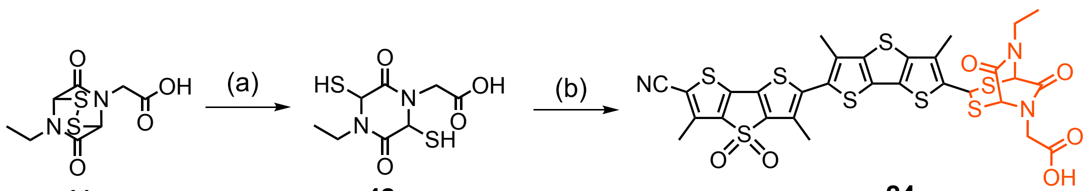

Scheme S9. Formation of **24** *in situ*. a) THPP, DMF, rt, 10 min; b) **7**, 4 M HCl in dioxane, CH_2Cl_2, rt, 48 h (incomplete conversion).

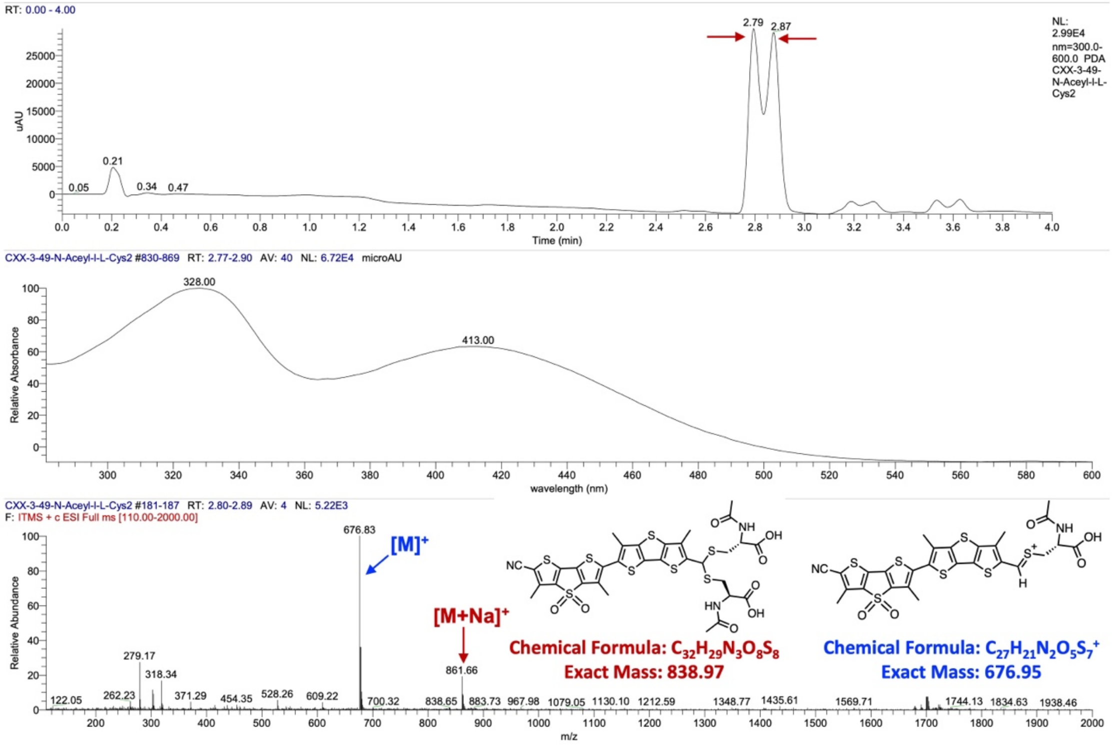

Figure S11. LCMS of flipper **17** made *in situ*.

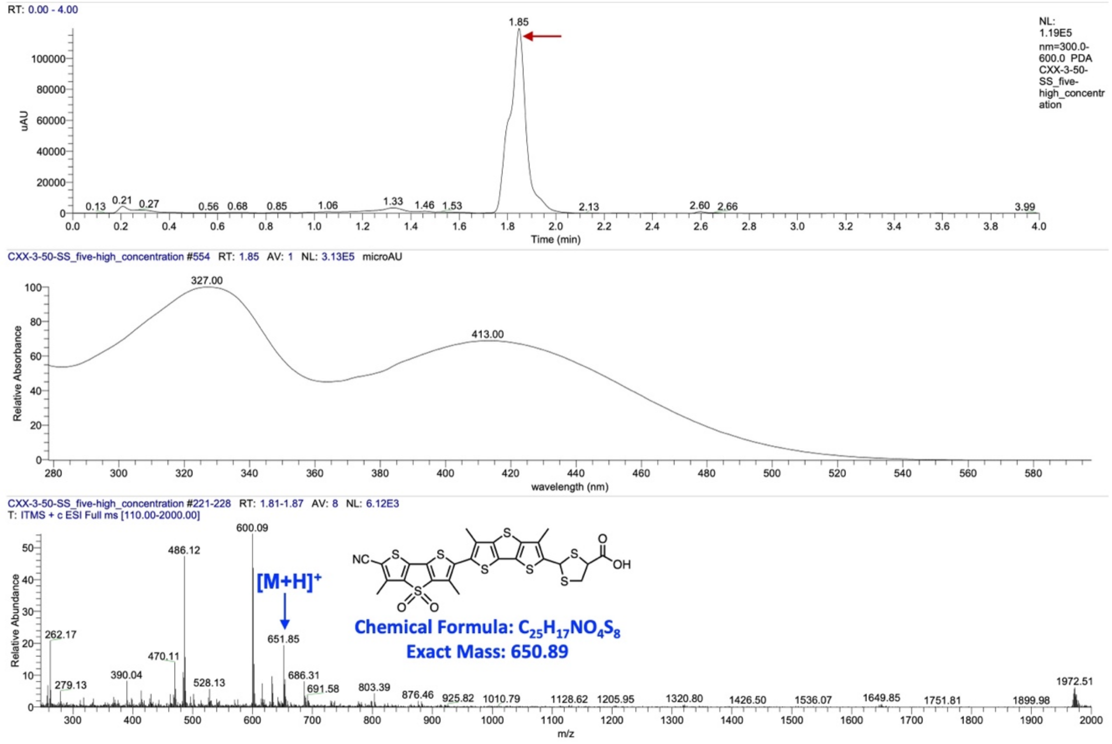

Figure S12. LCMS of flipper **18** made *in situ*.

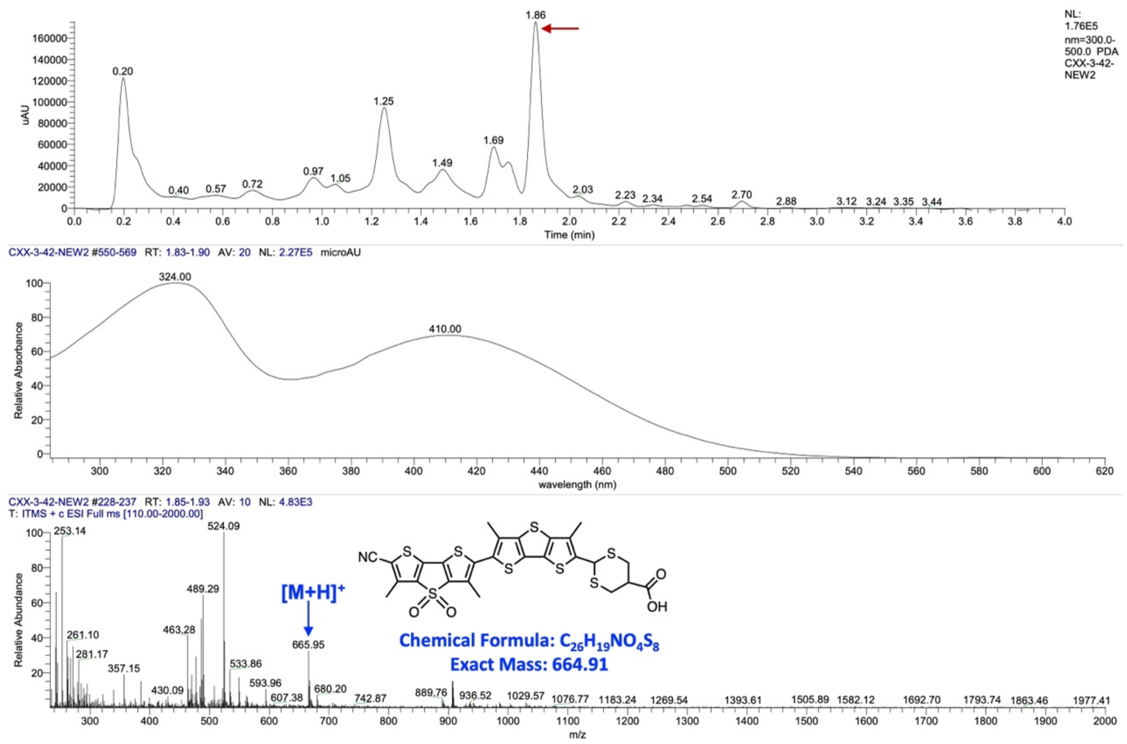

Figure S13. LCMS of flipper **19** made *in situ*.

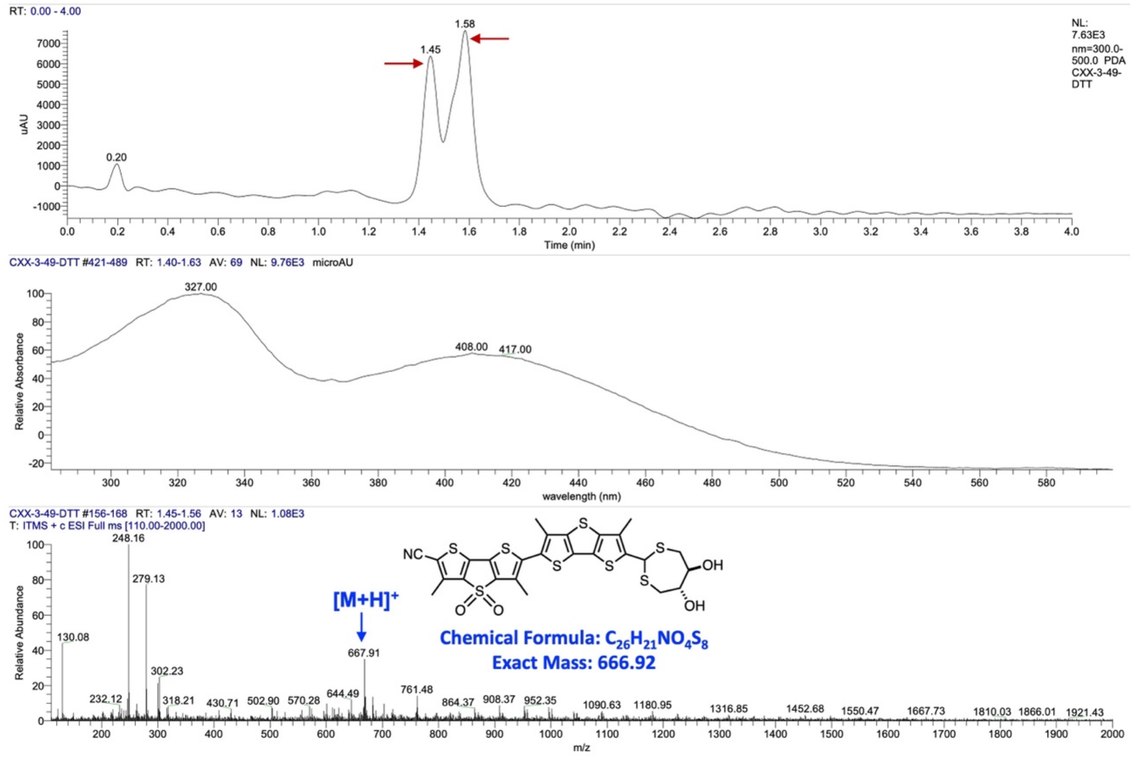

Figure S14. LCMS of flipper **20** made *in situ*.

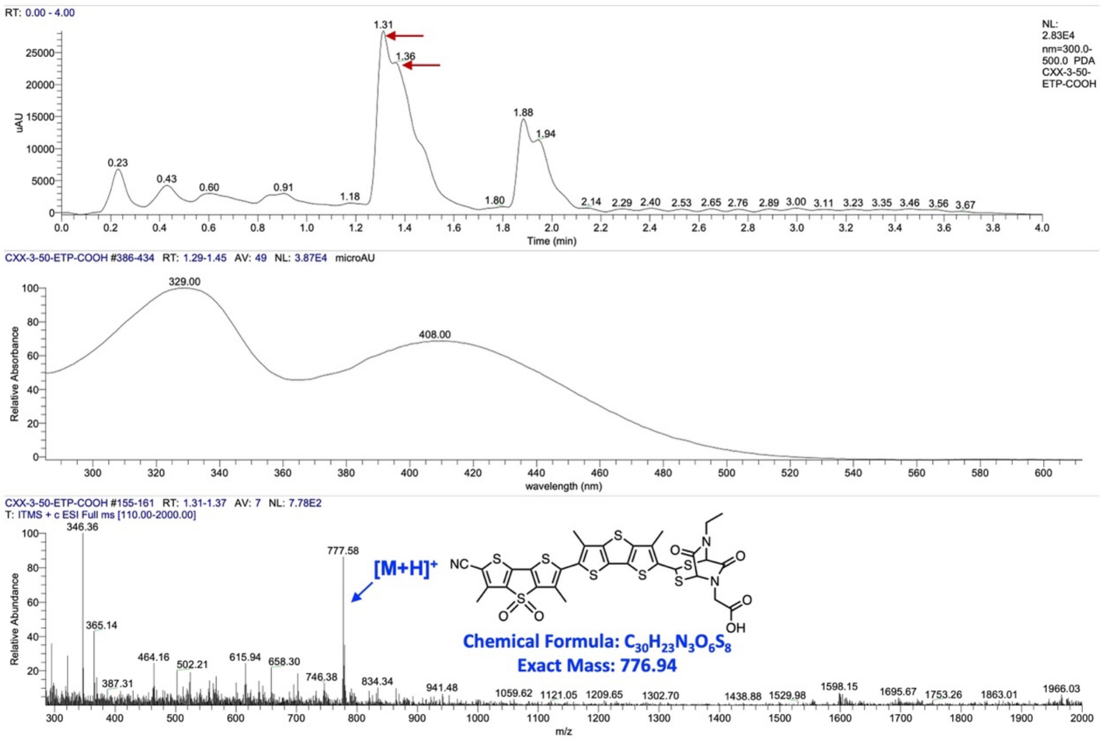

Figure S15. LCMS of flipper **24** made *in situ*.

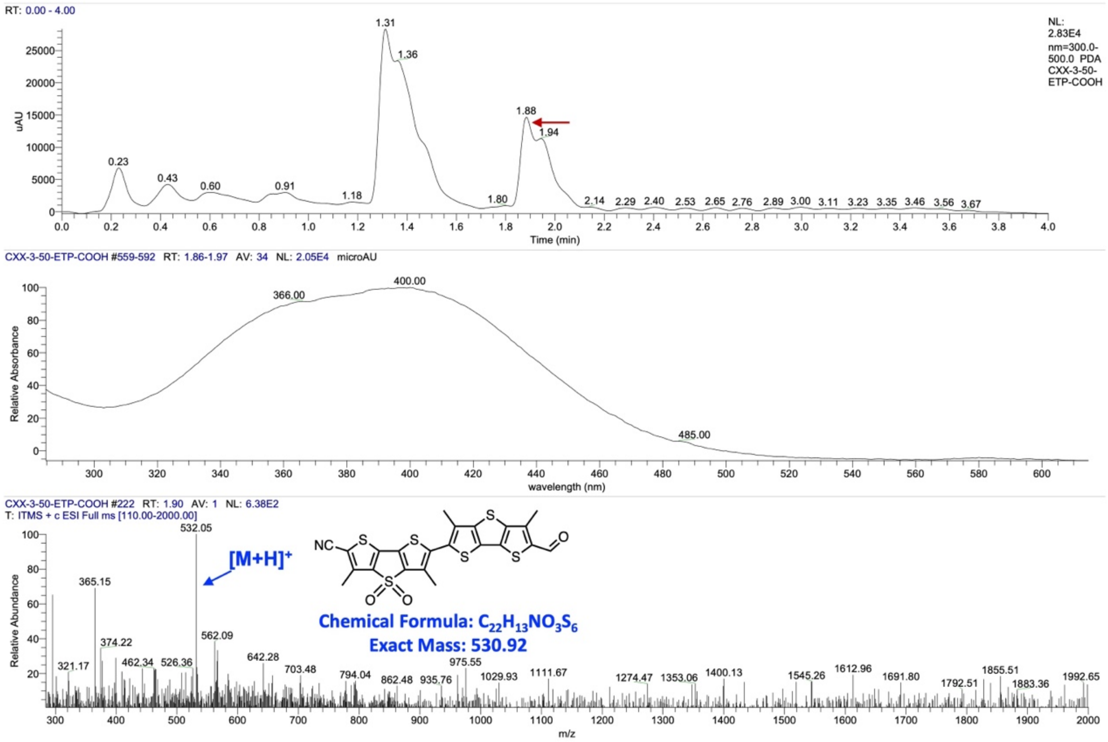

Figure S16. LCMS of **7** in flipper **24** made *in situ*.

Comment: Isometric peak splitting in LCMS originated from solubility limitations (partial aggregation), partial de/protonation, conformers and diastereomers in cyclic thio/acetals, etc.

5. Fluorescence Spectroscopy in LUVs

Large unilamellar vesicles (LUVs) stock solutions were prepared according to previously reported procedures.[S20, S21]

DOPC (1,2-dioleoyl-sn-glycero-3-phosphocholine) LUVs. A lipid film was prepared by evaporating a DOPC (23 mg, 31 μmol) solution in $CHCl_3$/ MeOH 9:1 (1 mL), then the flask was put under a vacuum overnight. The resulting film was hydrated with a buffer (1.0 mL, 10 mM Tris/Tris·HCl, 100 mM NaCl, pH 7.4) for 30 min at rt, subjected to freeze-thaw cycles (10×, liquid N_2, 55 °C water bath) and extruded (15×) through a polycarbonate membrane (pore size, 100 nm) using a Mini-extruder.

SM/CL (sphingomyelin/cholesterol) LUVs were prepared similarly using SM (15 mg, 21 μmol) and CL (3.5 mg, 9 μmol). Hydration (with 1 mL of the buffer) and extrusion were performed at 65 ºC.

DPPC (1,2-dipalmitoyl-sn-glycero-3-phosphocholine) LUVs were prepared similarly using DPPC (22 mg in MeOH/$CHCl_3$ 1:1, 30 μmol). Hydration (with 1 mL of the buffer) and extrusion were performed at 55 ºC.

General Methods. To a stirred Tris buffer solution (2.0 mL, 10 mM Tris/Tris·HCl, 100 mM NaCl, pH 7.4) were added different LUVs (5 μL of 30 mM lipid, 75 μM) at 50 °C. The probe (**22**, **19**, **16**, **6**, **23**, or **4**, 2 μL of a 0.1 mM stock solution in DMSO) was added to the diluted suspensions of LUVs solution (DOPC, SM/CL and DPPC), then the excitation (λ_{em} = 630 nm) and emission spectra (λ_{ex} = 412 nm) were recorded at 50 °C with regular intervals until the fluorescence intensity stabilized. Then, the temperature was lowered to 25 °C, and excitation (λ_{em} = 630 nm) and emission (λ_{ex} = 412 nm) spectra were measured at 25 °C with regular intervals until the fluorescence intensity stabilized.

The same mixture without probe was used as background. Excitation spectra obtained in ordered membranes were deconvoluted to 4 Gaussian peaks using fityk 1.3.1.

Concentration dependence in LUVs. The samples were prepared as described in the general procedure and kept for 30 min at 50 °C after the addition of different concentrations of probes (**22**, **19**, **16**, **23**, or **4**, 2 µL of a 0.02, 0.04, 0.08 and 0.1 mM stock solution in DMSO). Then, the temperature was lowered to 25 °C, and the excitation spectra (λ_{em} = 630 nm) were recorded 30 min after the temperature reached to 25 °C. There was a good linear relationship between probe concentration and maximum excitation intensity, and the shape of the excitation spectra was independent on probe concentration in this range.

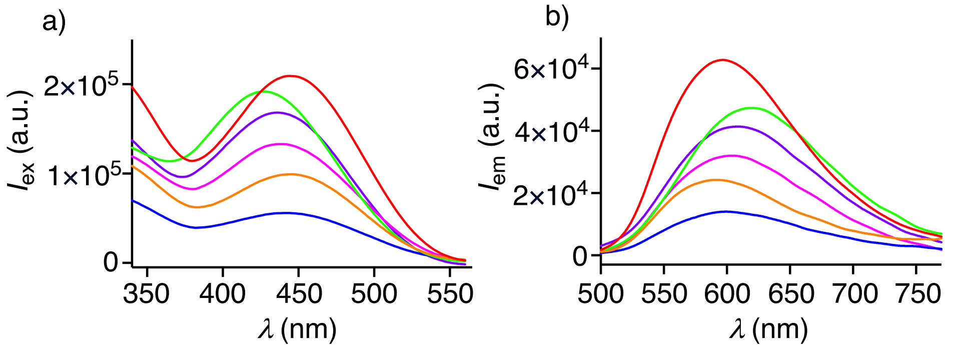

Figure S17. a) Excitation (λ_{em} = 630 nm) and b) emission (λ_{ex} = 412 nm) spectra of flipper probes in DOPC LUVs: **22** (100 nM, red line), **19** (100 nM, orange line), **16** (100 nM, green line), **6** (80 nM, blue line), **23** (100 nM, magenta line) and **4** (100 nM, purple line).

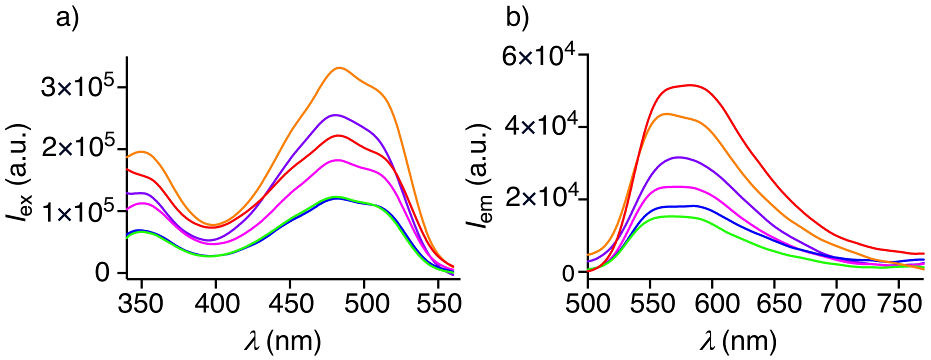

Figure S18. a) Excitation (λ_{em} = 630 nm) and b) emission (λ_{ex} = 412 nm) spectra of flipper probes in SMCL LUVs: **22** (100 nM, red line), **19** (100 nM, orange line), **16** (100 nM, green line), **6** (100 nM, blue line), **23** (100 nM, magenta line) and **4** (100 nM, purple line).

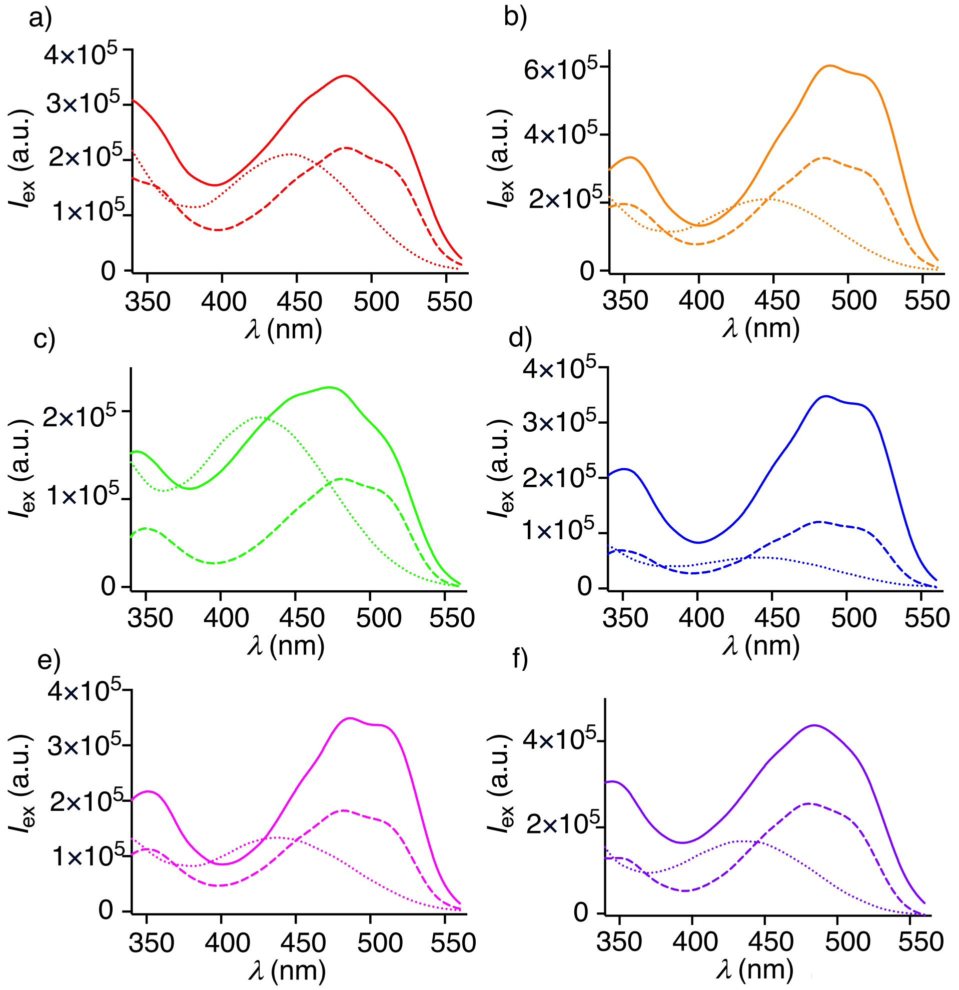

Figure S19. Excitation spectra of flipper probes (λ_{em} = 630 nm, 100 nM) a) **22**, b) **19**, c) **16**, d) **6**, e) **23**, f) **4** in DOPC LUVs (dotted line), SMCL LUVs (dashed line) and DPPC LUVs (solid line) at 25 °C.

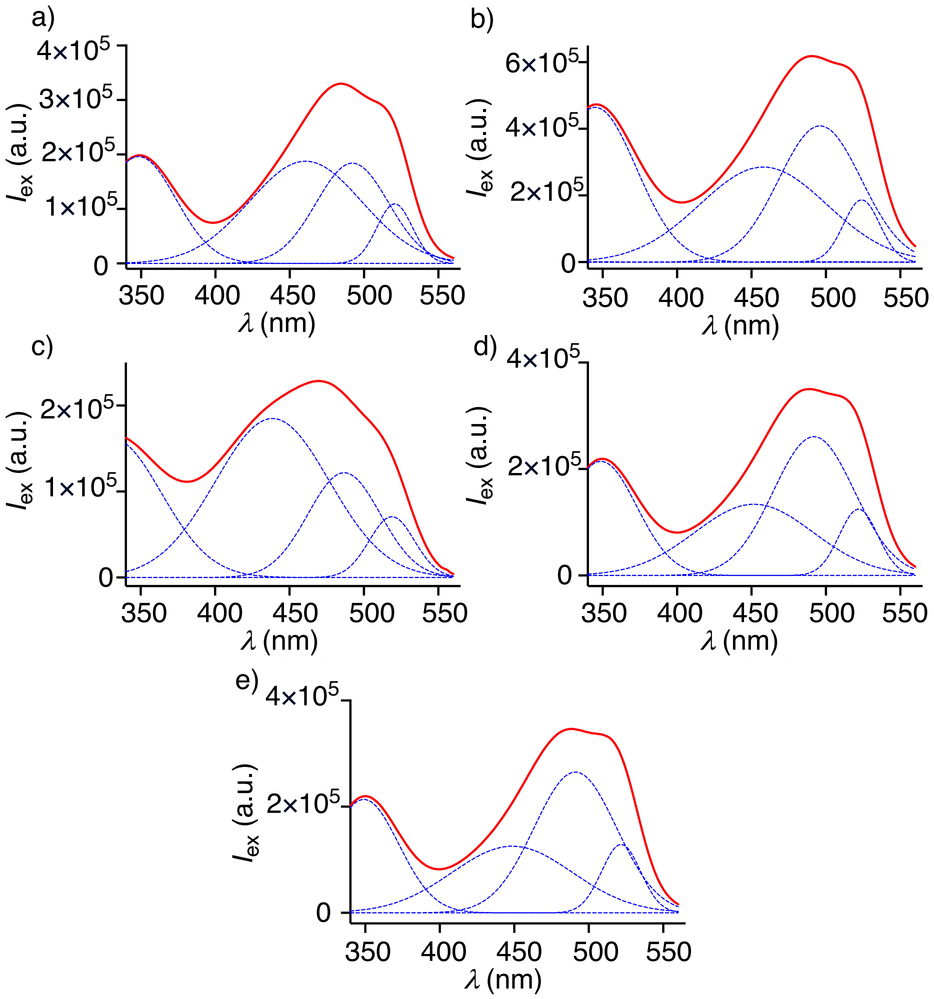

Figure S20. Deconvolution of the excitation spectra of flipper probes (λ_{em} = 630 nm, 100 nM) a) **22**, b) **19**, c) **16**, d) **6** and e) **23** in DPPC LUVs at 25 °C.

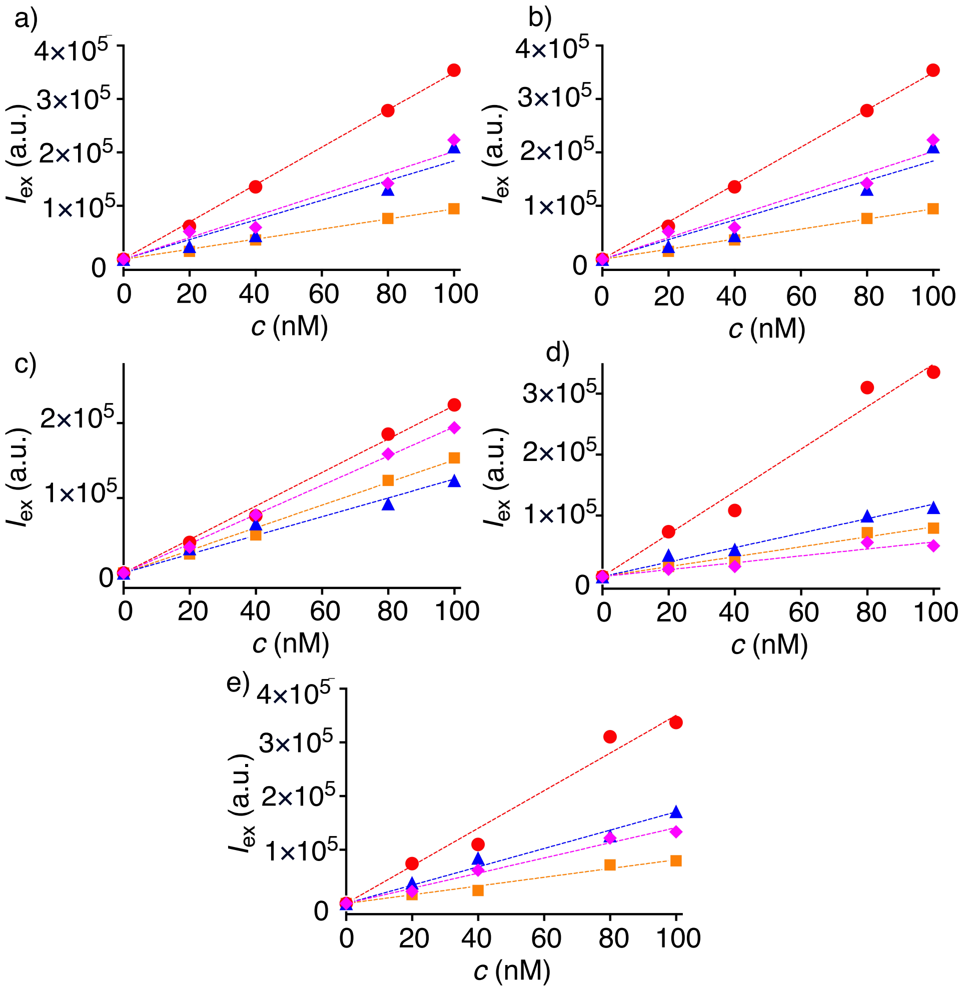

Figure S21. Dependence of maximum excitation intensities (λ_{em} = 630 nm) on concentrations of compounds a) **22,** b) **19**, c) **16**, d) **6**, e) **23**, in SM/CL LUVs at 25 °C (magenta diamonds), DOPC LUVs at 25 °C (blue triangle), DPPC LUVs at 25 °C (red circle) and DPPC LUVs at 50 °C (orange square).

6. Stability of Thio/Acetal Flipper

General Methods. To a stirred Tris buffer solution (2.0 mL, 10 mM Tris/Tris·HCl, 100 mM NaCl, pH 7.0) or sodium acetate buffer (2.0 mL, 10 mM NaOAc/HOAc, 100 mM NaCl, pH 5.2) were added SM/CL LUVs (5 μL of 30 mM lipid, 75 μM) at 25 °C. Then, the probe (**19** or **16**, 2 μL of a 0.1 mM stock solution in DMSO) was added. The emission spectra were recorded every 1 h upon excitation at λ_{ex} = 412 nm.

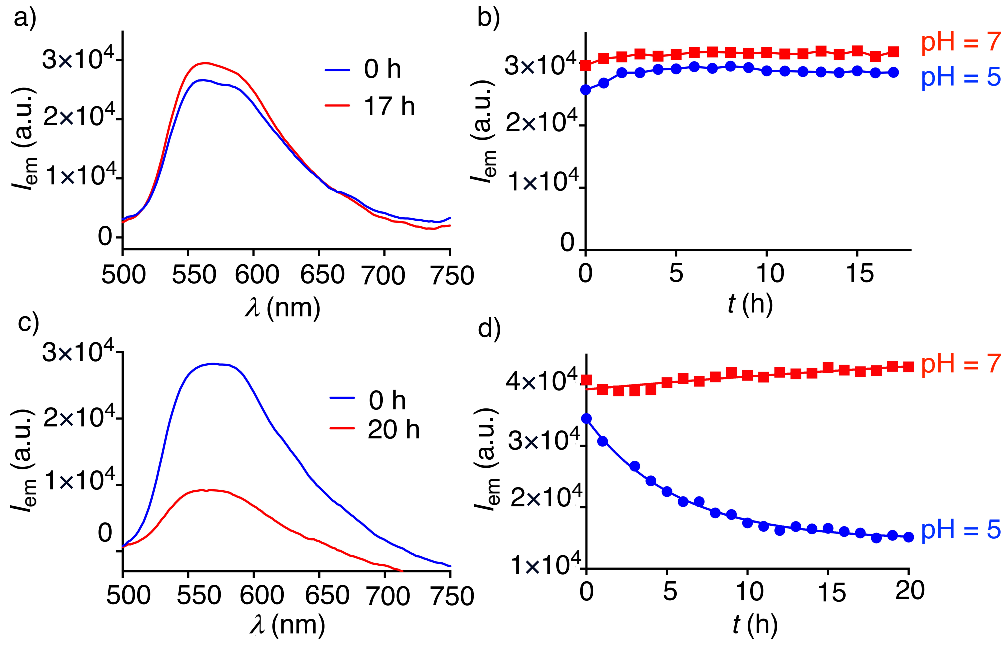

Figure S22. a) Time-dependent emission spectra of **19** in SM/CL LUVs at pH = 5.2; b) Emission intensity at 572 nm of **19** under pH = 7.0 (red square) and pH = 5.2 (blue circle); c) Time-dependent emission spectra of **16** in SM/CL LUVs at pH = 5.2; d) Emission intensity at 574 nm of **16** under pH = 7.0 (red square) and pH = 5.2 (blue circle). Data points were fit to the exponential decay function.

7. Fluorescence Lifetime Imaging Microscopy (FLIM)

GUVs preparation. GUVs were prepared by electroformation method using a Nanion Vesicle Prep Pro following reported procedures.[S20]

DOPC GUVs. A thin lipid film was prepared by evaporating a solution of DOPC (10 mM) in $CHCl_3$ (20 µL) on the conductive side of an ITO electrode and further drying under vacuum for 2 h. An o-ring covered in silicon grease was placed around the film, and 250 µL of an aqueous sucrose solution (250 mM) was added to the film. The second ITO electrode was placed on top of the first one, conductive side toward the joint and the sucrose solution. The electrodes were placed in the electroformation device and were exposed to an electric field of 1.2 V and 10 Hz for 2 h at 25 °C. This resulting stock solution of GUVs was used without further purification, after eventual dilution in Tris buffer (10 mM Tris/Tris·HCl, 100 mM NaCl, pH 7.4) for the microscopy experiments.

SM/CL GUVs. A thin lipid film was prepared by evaporating a preheated (55 °C) solution of SM/CL (7:3; 10 mM) in $CHCl_3$ with one drop of EtOH (20 µL) on the conductive side of a preheated ITO electrode (55 °C) and further drying under vacuum at 55 °C for 2 h. An o-ring covered in silicon grease was placed around the film, and 250 µL of an aqueous sucrose solution (250 mM) was added onto the film. The second ITO electrode was placed on top of the first one, conductive side toward the joint and the sucrose solution. The electrodes were placed in the electroformation device and were exposed to an electric field of 1.2 V and 10 Hz for 2 h at 55 °C. This resulting stock solution of GUVs was used without further purification, after eventual dilution in Tris buffer (10 mM Tris/ Tris·HCl, 100 mM NaCl, pH 7.4) for the microscopy experiments.

Phase separated GUVs were prepared analogously to SM/CL GUVs using a solution of DOPC/SM/CL (58:25:17) in chloroform with one drop of EtOH.

FLIM of GUVs. 10 μL of stock solutions of GUVs were added to 190 μL of Tris buffer followed by the addition of the probes **6, 16-20, 22-24** stock solutions in DMSO (0.2 or 0.4 μL of 1 mM: **6**, **16**, **19**, **22**, **23**, 0.6 mM: **17**, **19**, **20**, 0.3 mM: **18** and 0.5 mM: **24**) to reach a corresponding concentration (1 μM: **6**, **16**, **19**, **23**, 2 μM: **22**, 0.6 μM: **17**, **18**, **19**, **20**, 0.5 μM: **24**). The obtained mixture was placed on a 35 mm glass bottom dish (Mattek Corporation, P35G-1.5-14-C) and left for 15 min at rt before imaging. Laser at 480 nm (white light laser) was used and the fluorescence was collected between 550 and 650 nm, or laser at 489 nm was used and the fluorescence was collected between 570 and 616 nm. FLIM images were analysed by using Leica Application Suite Software LASX FLIM 4.5.0 Stellaris or SymPhoTime 64 software from PicoQuant. The presented are the longer lifetimes τ_1 calculated from a biexponential fit of the signal coming from GUVs (selected as ROI by "painting"). For each experiment, three pictures were acquired, and the reported values represent the mean ± standard deviation.

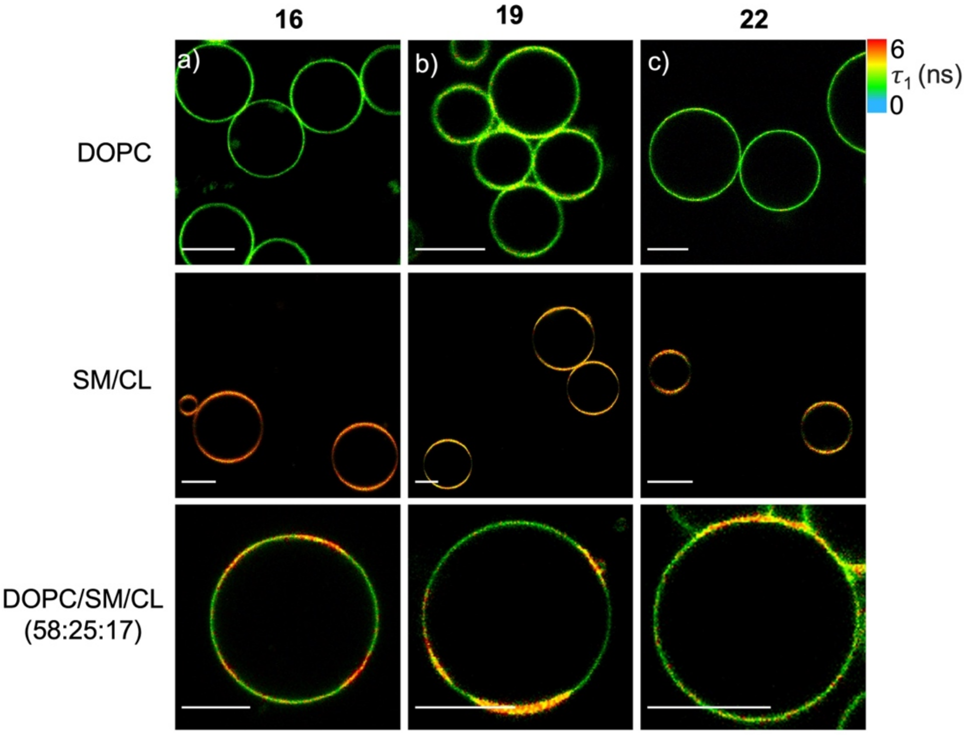

Figure S23. FLIM images of a) **16** (1 μM), b) **19** (1 μM), c) **22** (2 μM), in DOPC, SM/CL (7:3), and in phase separated DOPC/SM/CL (58:25:17) GUVs, scale bar: 10 μm, laser at 489 nm.

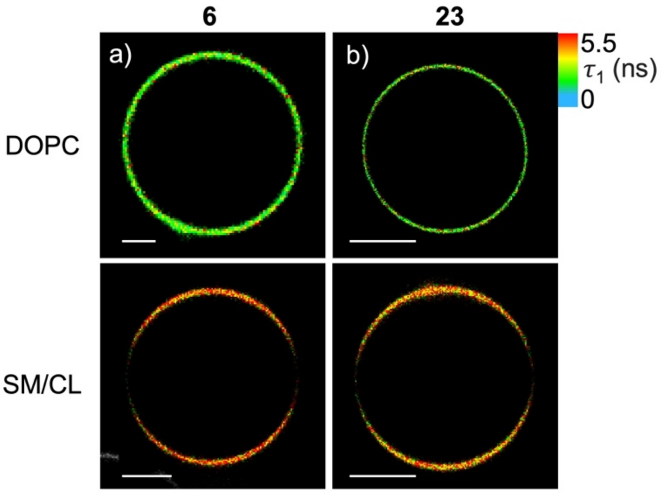

Figure S24. FLIM images of a) **6** (1 μM), b) **23** (1 μM), in DOPC and SM/CL (7:3) GUVs, scale bar: 2 μm, laser at 489 nm.

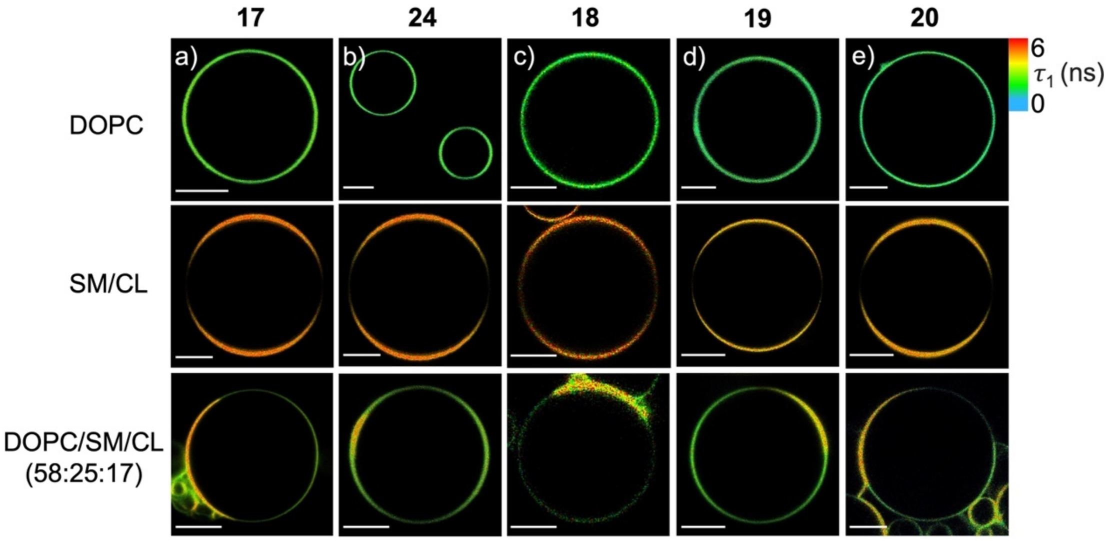

Figure S25. FLIM images of a) **17** (0.6 μM), b) **24** (0.5 μM), c) **18** (0.6 μM), d) **19** (0.6 μM), e) **20** (0.6 μM) generated *in situ* in DOPC, SM/CL (7:3), and in phase separated DOPC/SM/CL (58:25:17) GUVs, scale bar: 5 μm, **18**: laser at 489 nm, **17**, **24**, **19** and **20**: laser at 480 nm.

Cell preparation. As described in reference S20, HeLa Kyoto cells were cultured in 25 cm^2 cell culture flasks with a vent cap and grew in FluoroBrite DMEM (high D-Glucose, without phenol red) medium containing 10% fetal bovine serum (FBS), 1% Penicillin/Streptomycin (PS) and 1% Glutamine. 25 cm^2 tissue culture flask was kept under 5% CO_2 in a humidified atmosphere at 37 °C. The cells were detached by adding 1.0 mL of TrypLE Express at 37 °C for 3 min, followed by the addition of 1 mL of DMEM (same as above). For the microscopy experiment the cells were seeded at 8 × 10^4 cells/mL in 35 mm glass bottom dishes and kept at 37 °C at 5% CO_2 overnight.

FLIM measurements in HeLa Kyoto cells. The cells were washed (3 x 1 mL) with PBS buffer and incubated with DMEM medium containing the corresponding probe (0.6 μM for **18**, 1 μM for other probes, 1 mL) for 10 minutes at 37 °C at 5 % CO_2. The images were acquired without exchanging the incubation medium or additional washing. The hypertonic shock was achieved by adding 1 mL of 1 M (0.5 M for **6**) sucrose medium containing the corresponding probe (0.6 μM for **18**, 1 μM for other probes) in the dish containing 1 mL isotonic medium for 30 min. For the analysis,

SymPhoTime 64 software (PicoQuant) was used to fit fluorescent decay data (at least 6 cells per picture, 4 cells for **23**) to a dual exponential deconvolution model where the lifetime τ was extracted. The lifetimes τ_1 and τ_{av} are both average of at least 6 cells and the reported values represent the mean ± standard deviation. ROIs were selected by "painting" for the plasma membrane only measurements.

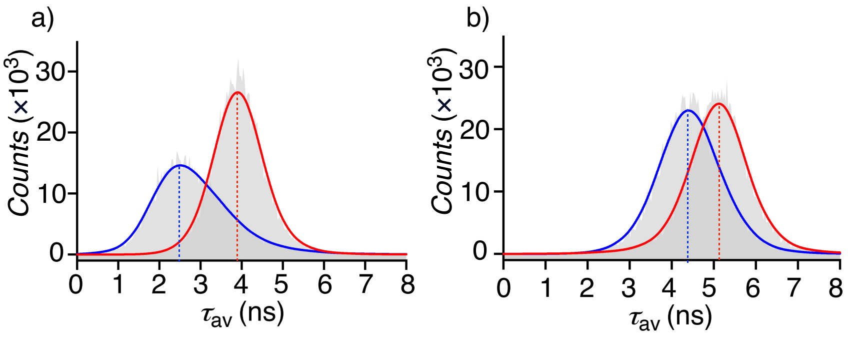

Figure S26. Corresponding τ_{av} histograms of a) **6** and b) **4** before (red) and after hypertonic shock (blue).

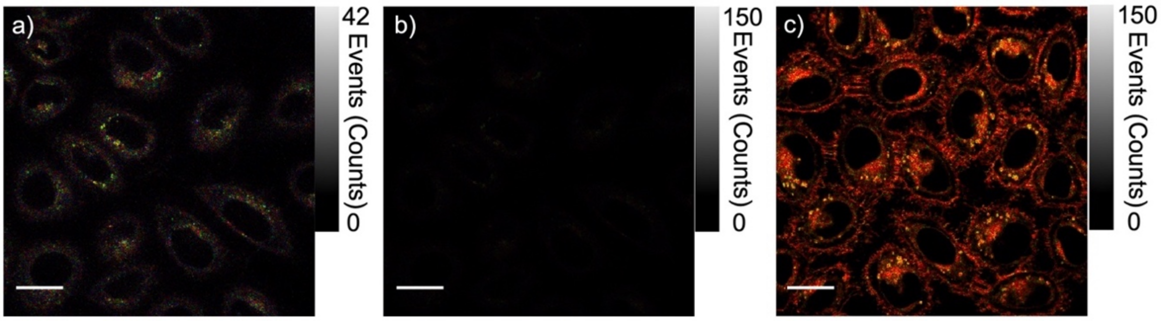

Figure S27. FLIM images of **7** (a, b; at different intensity scale) and **24** (c) in HK cells, scale bar: 20 µm. *Comment:* since fluorescence counts of compound **7** in FLIM images were much lower than those of **24**, remaining **7** with *in-situ* generated **24** (Figure S15 and S16) should not contribute to lifetime images.

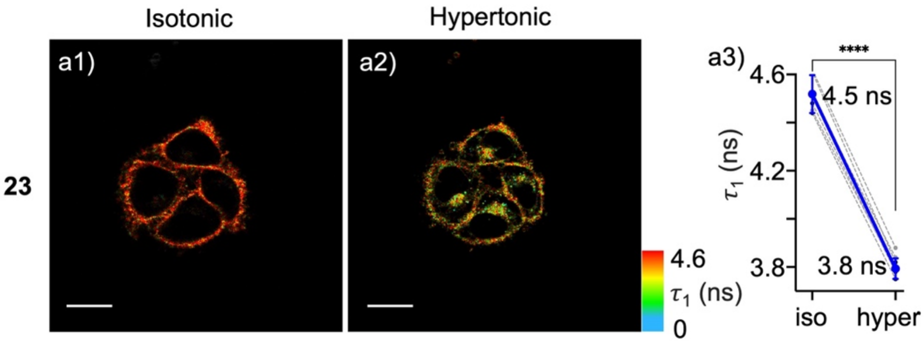

Figure S28. FLIM images of **23** in HK cells before (a1) and after (a2) hypertonic shock, with the corresponding fluorescence lifetimes of plasma membrane under isotonic (iso) and hypertonic (hyper) conditions (a3), 1 dot = 1 measurement; solid thick blue line, mean value; whiskers, standard deviation; dashed thin grey lines, measurements with the same cell; statistical significance determined with the one tailed paired student's *t*-test, ****: $p < 0.0001$, scale bar: 20 µm.

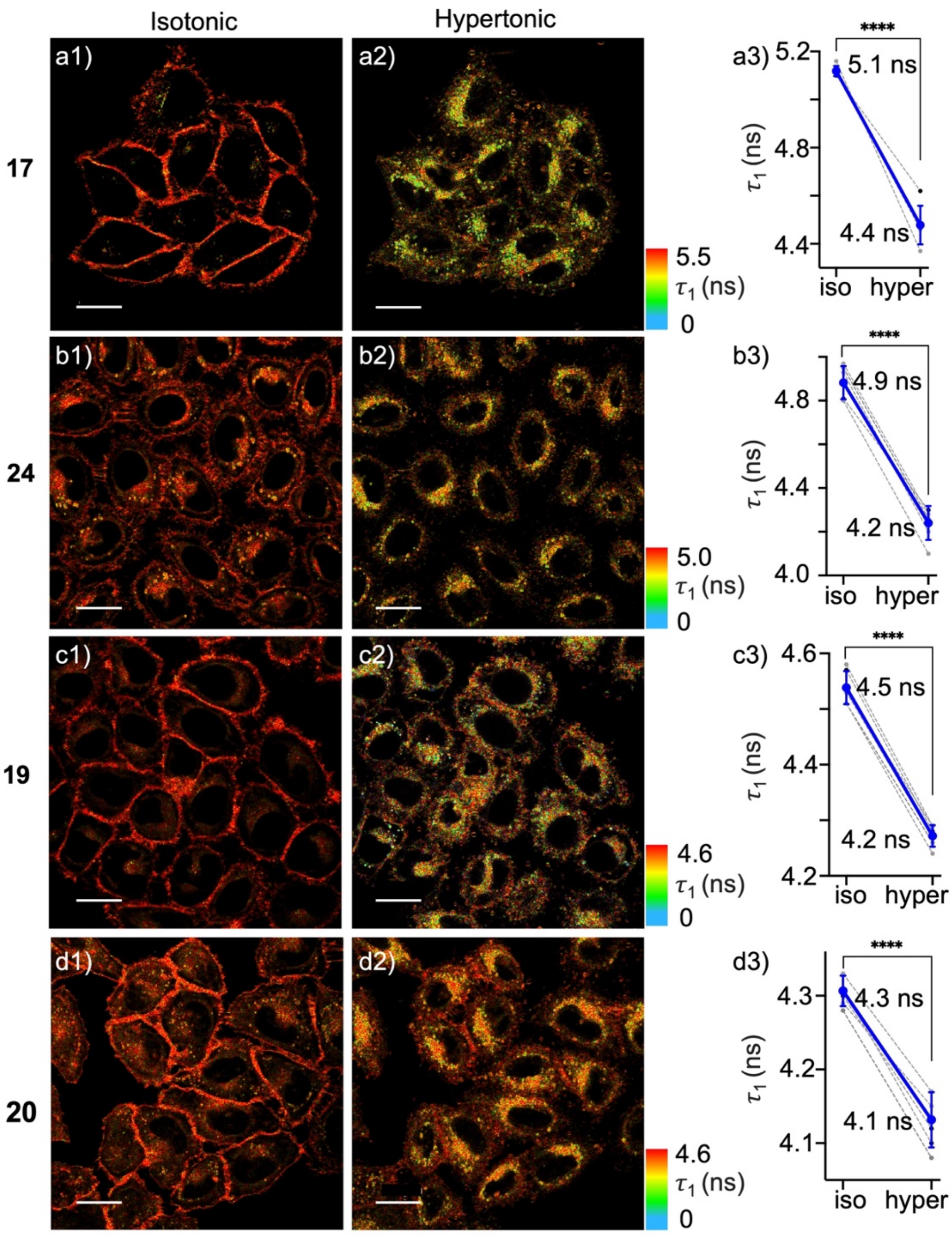

Figure S29. FLIM images of compounds generated *in situ* in HK cells before (a1: **17**, b1: **24**, c1: **19**, d1: **20**) and after (a2: **17**, b2: **24**, c2: **19**, d2: **20**) hypertonic shock, with the corresponding fluorescence lifetimes of plasma membrane under isotonic (iso) and hypertonic (hyper) conditions (a3: **17**, b3: **24**, c3: **19**, d3: **20**), 1 dot = 1 measurement; solid thick blue line, mean value; whiskers, standard deviation; dashed thin gray lines, measurements with the same cell; statistical significance determined with the one tailed paired student's *t*-test, ****: $p < 0.0001$, scale bar: 20 µm

8. Supporting References

(S1) López-Andarias, J.; Eblighatian, K.; Pasquer, Q. T. L.; Assies, L.; Sakai, N.; Hoogendoorn, S.; Matile, S. Photocleavable Fluorescent Membrane Tension Probes: Fast Release with Spatiotemporal Control in Inner Leaflets of Plasma Membrane, Nuclear Envelope, and Secretory Pathway. *Angew. Chem. Int. Ed.* **2022**, *61*, e202113163.

(S2) Macchione, M.; Tsemperouli, M.; Goujon, A.; Mallia, A. R.; Sakai, N.; Sugihara, K.; Matile, S. Mechanosensitive Oligodithienothiophenes: Transmembrane Anion Transport Along Chalcogen-Bonding Cascades. *Helv. Chim. Acta* **2018**, *101*, e1800014.

(S3) Dal Molin, M.; Verolet, Q.; Colom, A.; Letrun, R.; Derivery, E.; Gonzalez-Gaitan, M.; Vauthey, E.; Roux, A.; Sakai, N.; Matile, S. Fluorescent Flippers for Mechanosensitive Membrane Probes. *J. Am. Chem. Soc.* **2015**, *137*, 568–571.

(S4) Singh, R.; Whitesides, G. M. Comparisons of Rate Constants for Thiolate-Disulfide Interchange in Water and in Polar Aprotic Solvents Using Dynamic Proton NMR Line Shape Analysis. *J. Am. Chem. Soc.* **1990**, *112*, 1190–1197.

(S5) Danehy, J. P.; Elia, V. J. Alkaline Decomposition of Organic Disulfides. VI. Further Examples of Elimination Reactions (1,2-Dithiolanecarboxylic Acids) and of Nucleophilic Substitution. *J. Org. Chem.* **1972**, *37*, 369–373.

(S6) Inagaki, F.; Momose, M.; Maruyama, N.; Matsuura, K.; Matsunaga, T.; Mukai, C. Activation of Disulfide Bond Cleavage Triggered by Hydrophobization and Lipophilization of Functionalized Dihydroasparagusic Acid. *Org. Biomol. Chem.* **2018**, *16*, 4320–4324.

(S7) Zhou, S.-W.; Tong, F.; Chen, M.; Gu, R.; Shi, C.-Y.; Yu, C.-Y.; Zhang, Q.; Qu, D.-H. Self-Evolution of High Mechanical Strength Dry-Network Polythiourethane Thermosets into Neat Macroscopic Hollow Structures. *Angew. Chem. Int. Ed.* **2022**, *61*, e202117195.

(S8) Liehl, E.; Unger, F. M. 1,2-Dithiolane Derivatives, Process for Their Production

Pharmaceutical Compositions Containing Them and Their Use. GB2148296A, May 30, 1985

(S9) Cheng, Y.; Pham, A.-T.; Kato, T.; Lim, B.; Moreau, D.; López-Andarias, J.; Zong, L.; Sakai, N.; Matile, S. Inhibitors of Thiol-Mediated Uptake. *Chem. Sci.* **2021**, *12*, 626-631.

(S10) Adamo, C.; Barone, V. Toward Reliable Density Functional Methods without Adjustable Parameters: The PBE0 Model. *J. Chem. Phys.* **1999**, *110*, 6158–6170.

(S11) Grimme, S.; Antony, J.; Ehrlich, S.; Krieg, H. A Consistent and Accurate Ab Initio Parametrization of Density Functional Dispersion Correction (DFT-D) for the 94 Elements H-Pu. *J. Chem. Phys.* **2010**, *132*, 154104.

(S12) Weigend, F.; Ahlrichs, R. Balanced Basis Sets of Split Valence, Triple Zeta Valence and Quadruple Zeta Valence Quality for H to Rn: Design and Assessment of Accuracy. *Phys. Chem. Chem. Phys.* **2005**, *7*, 3297–3305.

(S13) Ahlrichs, R.; Bär, M.; Häser, M.; Horn, H.; Kölmel, C. Electronic Structure Calculations on Workstation Computers: The Program System Turbomole. *Chem. Phys. Lett.* **1989**, *162*, 165–169.

(S14) Glendening, E. D.; Landis, C. R.; Weinhold, F. Natural Bond Orbital Methods. *WIREs Comput. Mol. Sci.* **2012**, *2*, 1–42.

(S15) Glendening, E. D.; Landis, C. R.; Weinhold, F. NBO 7.0: New Vistas in Localized and Delocalized Chemical Bonding Theory. *J. Comput. Chem.* **2019**, *40*, 2234–2241.

(S16) Johnson, E. R.; Keinan, S.; Mori-Sánchez, P.; Contreras-García, J.; Cohen, A. J.; Yang, W. Revealing Noncovalent Interactions. *J. Am. Chem. Soc.* **2010**, *132*, 6498–6506.

(S17) Contreras-García, J.; Johnson, E. R.; Keinan, S.; Chaudret, R.; Piquemal, J.-P.; Beratan, D. N.; Yang, W. NCIPLOT: A Program for Plotting Noncovalent Interaction Regions. *J. Chem. Theory Comput.* **2011**, *7*, 625–632.

(S18) Humphrey, W.; Dalke, A.; Schulten, K. VMD: Visual Molecular Dynamics. *J. Mol. Graph.*

1996, *14*, 33–38.

(S19) Zong, L.; Bartolami, E.; Abegg, D.; Adibekian, A.; Sakai, N.; Matile, S. Epidithiodiketopiperazines: Strain-Promoted Thiol-Mediated Cellular Uptake at the Highest Tension. *ACS Cent. Sci.* **2017**, *3*, 449–453.

(S20) García-Calvo, J.; Maillard, J.; Fureraj, I.; Strakova, K.; Colom, A.; Mercier, V.; Roux, A.; Vauthey, E.; Sakai, N.; Fürstenberg, A.; Matile, S. Fluorescent Membrane Tension Probes for Super-Resolution Microscopy: Combining Mechanosensitive Cascade Switching with Dynamic-Covalent Ketone Chemistry. *J. Am. Chem. Soc.* **2020**, *142*, 12034–12038.

(S21) López-Andarias, J.; Straková, K.; Martinent, R.; Jiménez-Rojo, N.; Riezman, H.; Sakai, N.; Matile, S. Genetically Encoded Supramolecular Targeting of Fluorescent Membrane Tension Probes within Live Cells: Precisely Localized Controlled Release by External Chemical Stimulation. *JACS Au* **2021**, *1*, 221–232.

The original data can be found at: https://doi.org/10.5281/zenodo.8207278

9. NMR Spectra

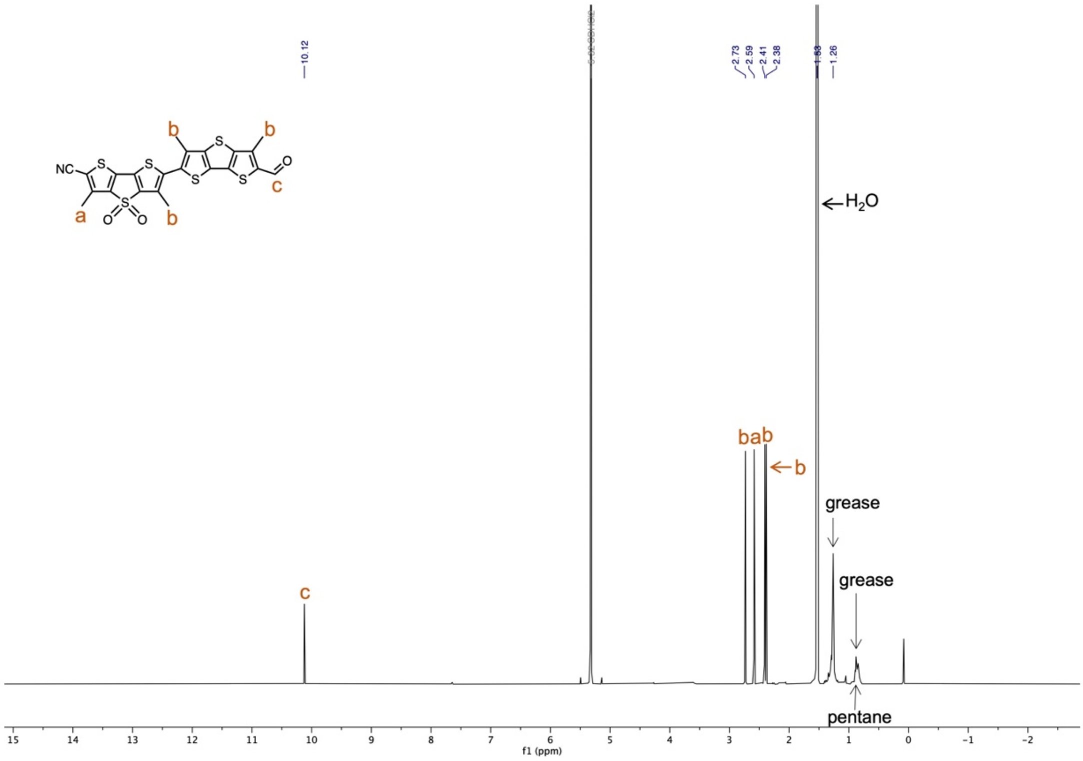

Figure S30. 500 MHz ^{1}H NMR spectrum of **7** in CD_2Cl_2.

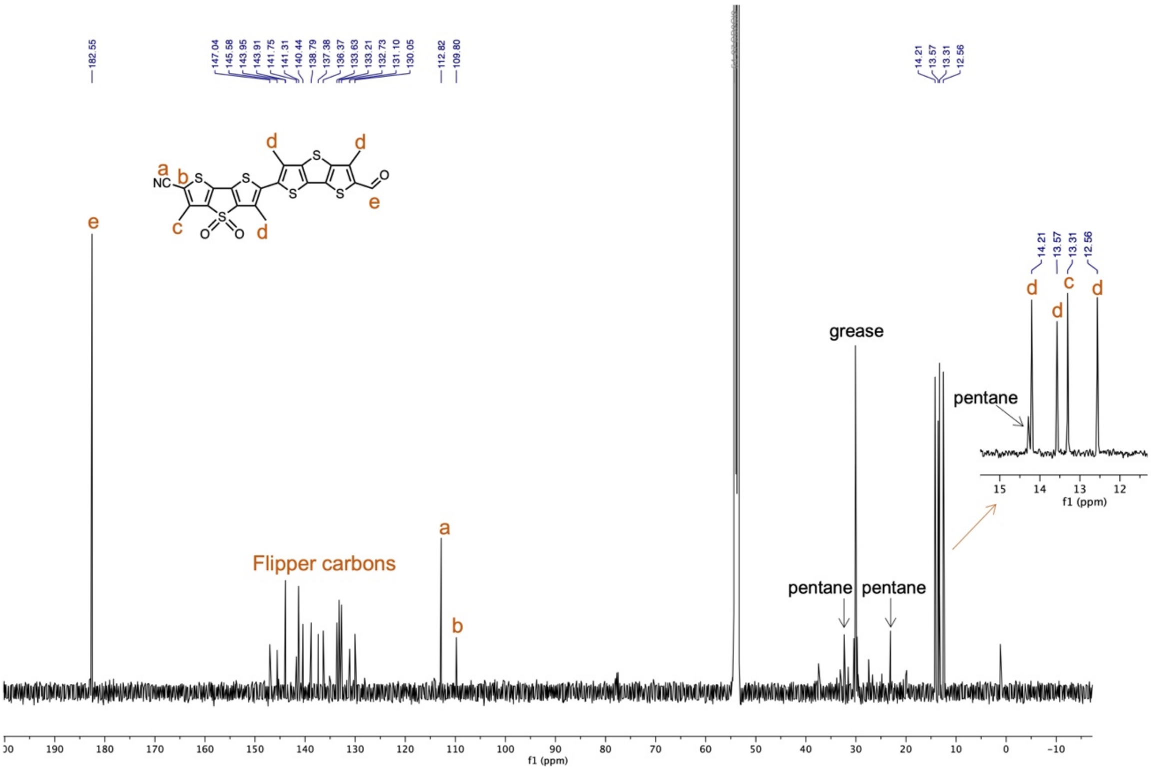

Figure S31. 126 MHz ^{13}C NMR spectrum of **7** in CD_2Cl_2.

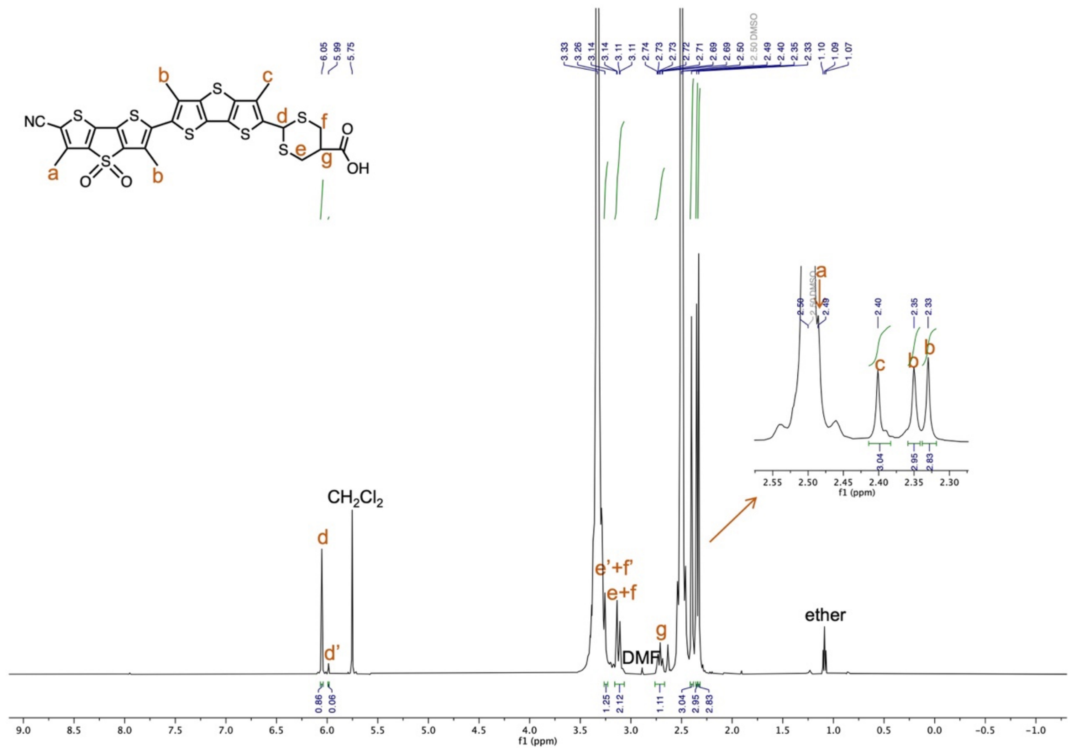

Figure S32. 500 MHz ^{1}H NMR spectrum of **19** in DMSO-d_6.

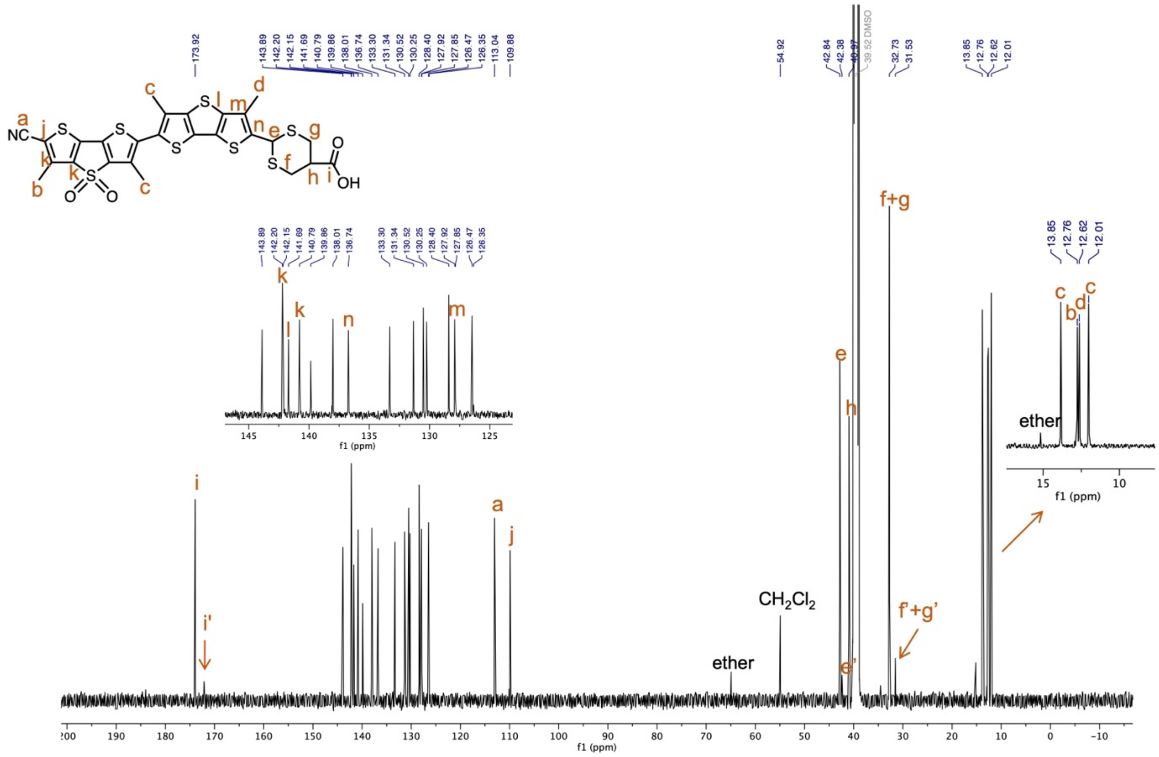

Figure S33. 126 MHz ^{13}C NMR spectrum of **19** in DMSO-d_6.

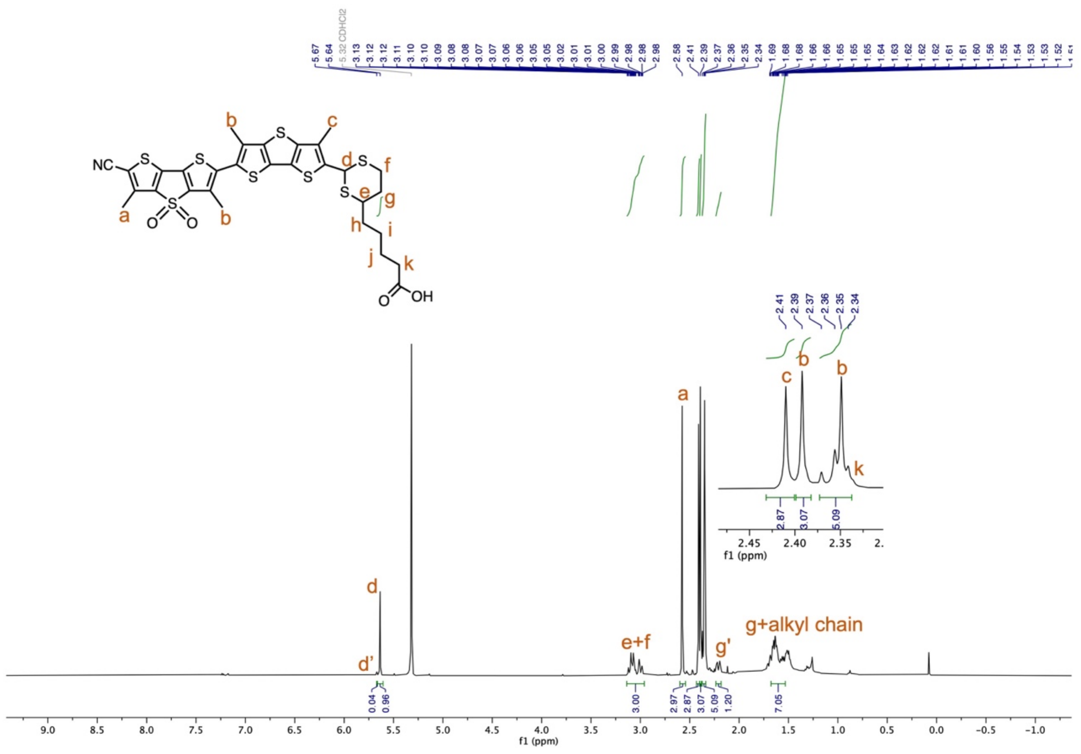

Figure S34. 500 MHz ^{1}H NMR spectrum of **22** in CD_2Cl_2.

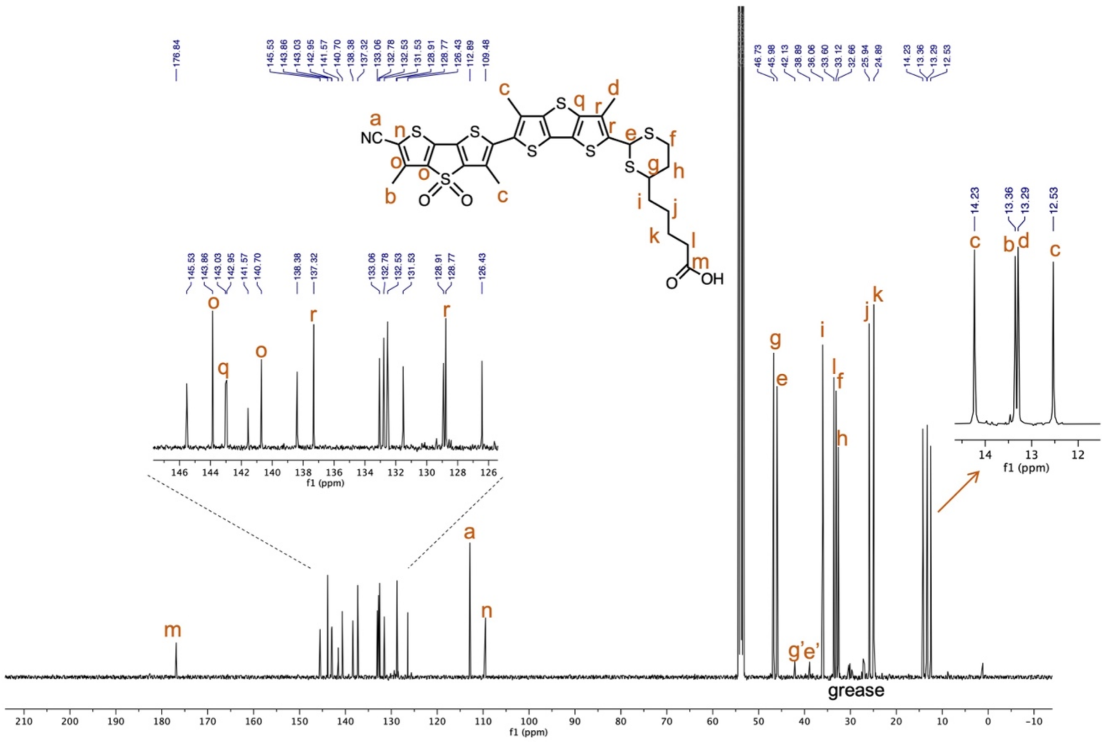

Figure S35. 126 MHz ^{13}C NMR spectrum of **22** in CD_2Cl_2.

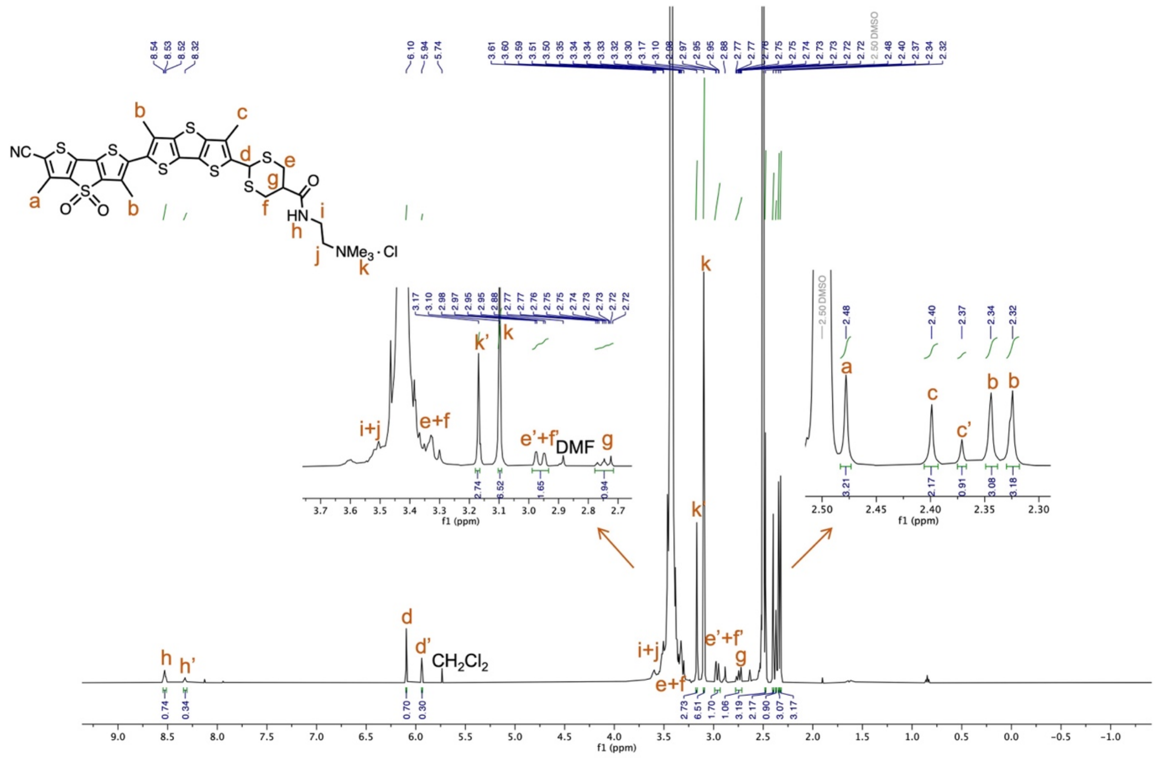

Figure S36. 500 MHz ^{1}H NMR spectrum of **6** in DMSO-d_6.

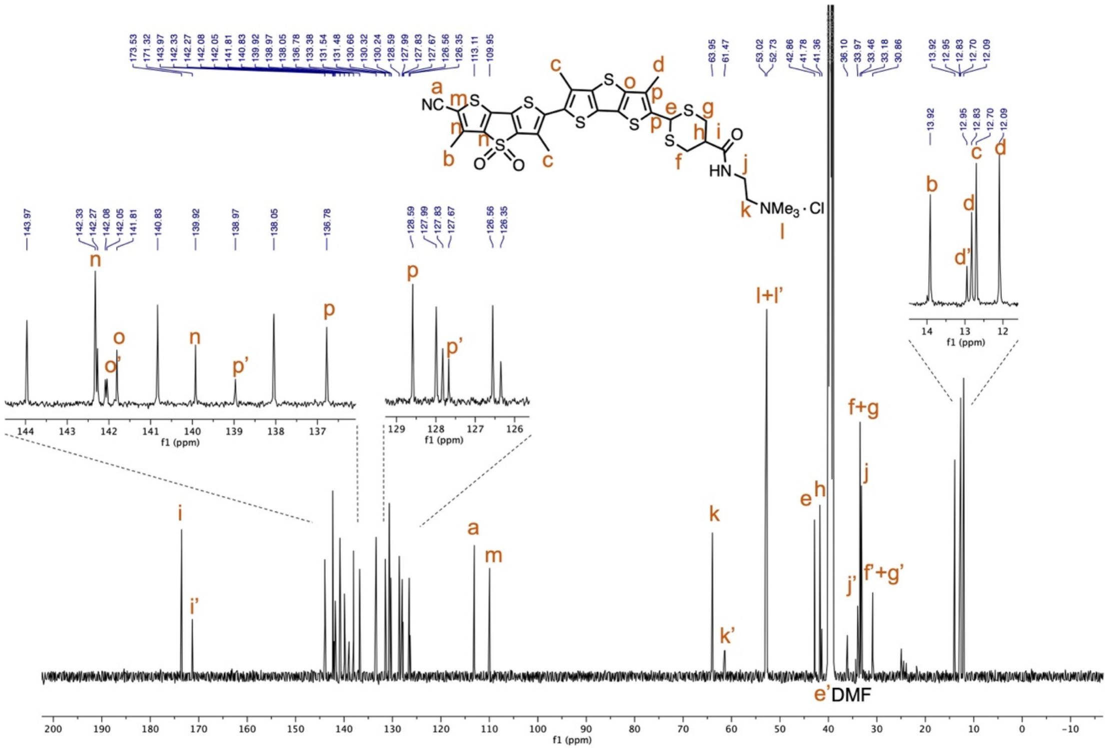

Figure S37. 126 MHz ^{13}C NMR spectrum of **6** in DMSO-d_6.

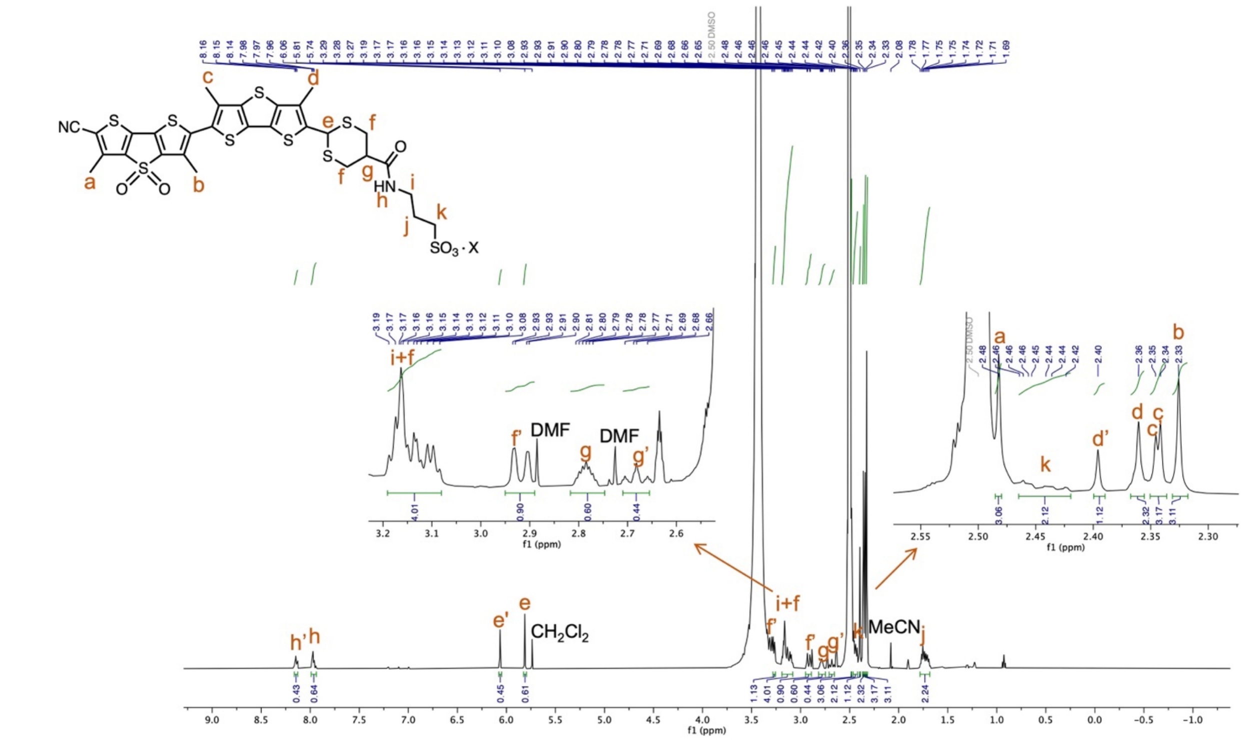

Figure S38. 500 MHz ^{1}H spectrum of **23** in DMSO-d_6.

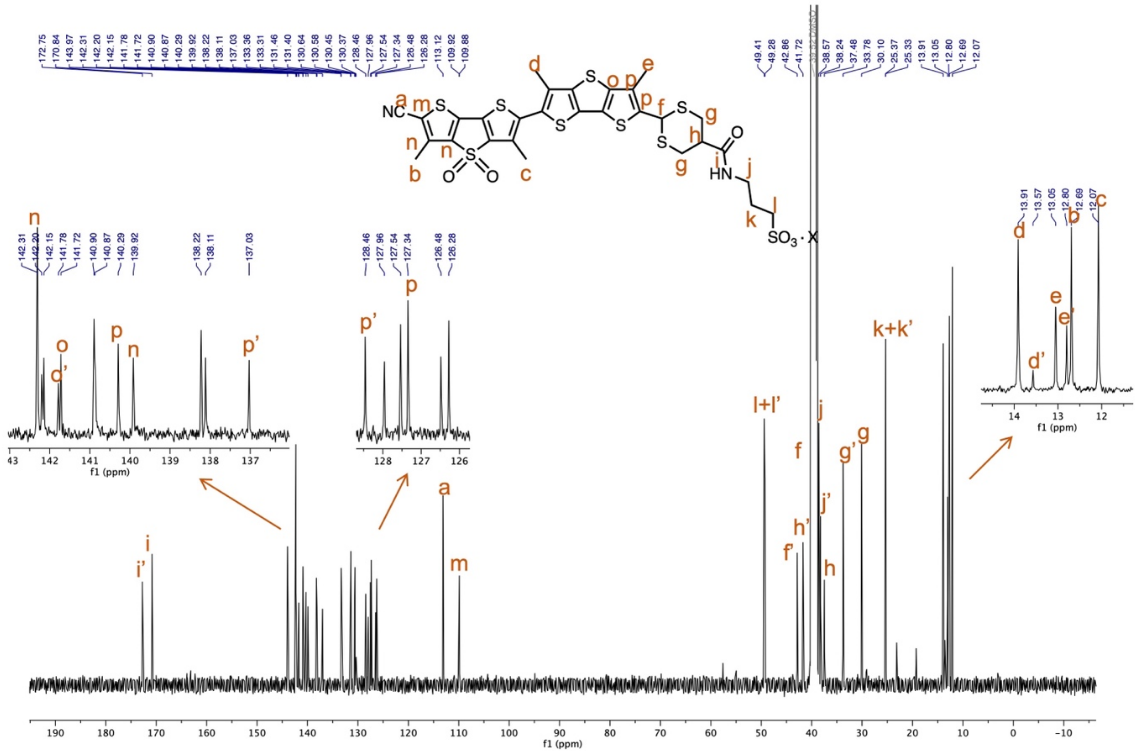

Figure S39. 126 MHz ^{13}C NMR spectrum of **23** in DMSO-d_6.

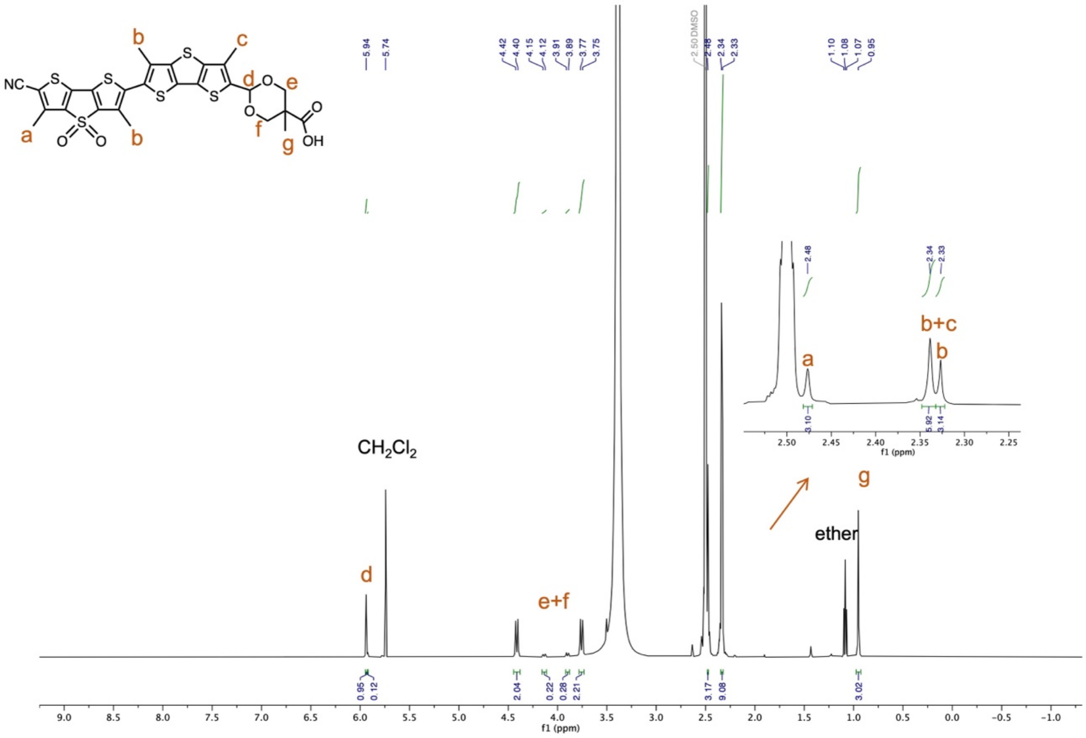

Figure S40. 500 MHz ^{1}H NMR spectrum of **16** in DMSO-d_6.

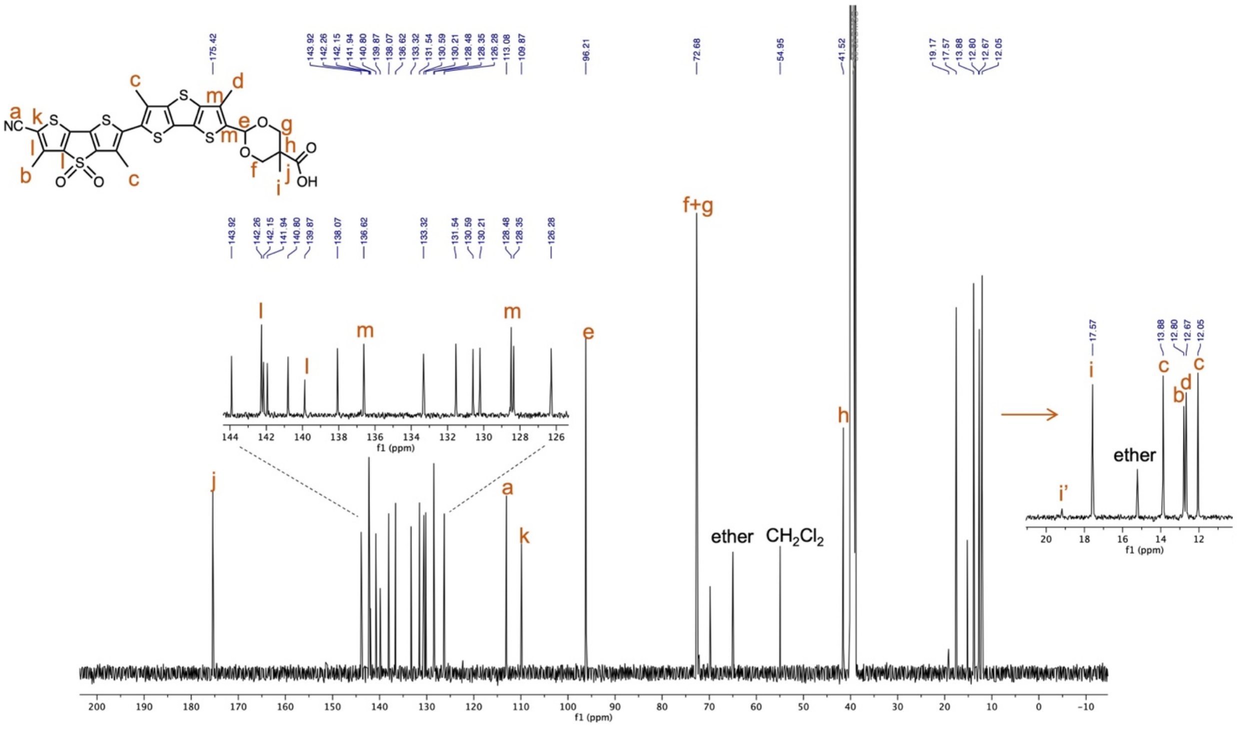

Figure S41. 126 MHz ^{13}C NMR spectrum of **16** in DMSO-d_6.
